# Peroxisomal defects in microglial cells induce a disease-associated microglial signature

**DOI:** 10.3389/fnmol.2023.1170313

**Published:** 2023-04-17

**Authors:** Quentin Raas, Ali Tawbeh, Mounia Tahri-Joutey, Catherine Gondcaille, Céline Keime, Romain Kaiser, Doriane Trompier, Boubker Nasser, Valerio Leoni, Emma Bellanger, Maud Boussand, Yannick Hamon, Alexandre Benani, Francesca Di Cara, Caroline Truntzer, Mustapha Cherkaoui-Malki, Pierre Andreoletti, Stéphane Savary

**Affiliations:** ^1^Laboratoire Bio-PeroxIL EA7270, University of Bourgogne, Dijon, France; ^2^Laboratory of Biochemistry, Neurosciences, Natural Resources and Environment, Faculty of Sciences and Techniques, University Hassan I, Settat, Morocco; ^3^Plateforme GenomEast, IGBMC, CNRS UMR 7104, Inserm U1258, University of Strasbourg, Illkirch, France; ^4^Laboratory of Clinical Biochemistry, Hospital of Desio, ASST-Brianza and Department of Medicine and Surgery, University of Milano-Bicocca, Monza, Italy; ^5^Aix Marseille Univ, CNRS, INSERM, CIML, Marseille, France; ^6^Centre des Sciences du Goût et de l’Alimentation, CNRS, INRAE, Institut Agro Dijon, University of Bourgogne Franche-Comté, Dijon, France; ^7^Department of Microbiology and Immunology, IWK Health Centre, Dalhousie University, Halifax, NS, Canada; ^8^Platform of Transfer in Biological Oncology, Georges François Leclerc Cancer Center–Unicancer, Dijon, France

**Keywords:** peroxisome, adrenoleukodystrophy (X-ALD), microglia, lysosome, lipid metabolism, autophagy

## Abstract

Microglial cells ensure essential roles in brain homeostasis. In pathological condition, microglia adopt a common signature, called disease-associated microglial (DAM) signature, characterized by the loss of homeostatic genes and the induction of disease-associated genes. In X-linked adrenoleukodystrophy (X-ALD), the most common peroxisomal disease, microglial defect has been shown to precede myelin degradation and may actively contribute to the neurodegenerative process. We previously established BV-2 microglial cell models bearing mutations in peroxisomal genes that recapitulate some of the hallmarks of the peroxisomal β-oxidation defects such as very long-chain fatty acid (VLCFA) accumulation. In these cell lines, we used RNA-sequencing and identified large-scale reprogramming for genes involved in lipid metabolism, immune response, cell signaling, lysosome and autophagy, as well as a DAM-like signature. We highlighted cholesterol accumulation in plasma membranes and observed autophagy patterns in the cell mutants. We confirmed the upregulation or downregulation at the protein level for a few selected genes that mostly corroborated our observations and clearly demonstrated increased expression and secretion of DAM proteins in the BV-2 mutant cells. In conclusion, the peroxisomal defects in microglial cells not only impact on VLCFA metabolism but also force microglial cells to adopt a pathological phenotype likely representing a key contributor to the pathogenesis of peroxisomal disorders.

## Introduction

Microglial cells are small stellate cells that play an essential role in brain homeostasis (Wright-Jin and Gutmann, 2019). They are involved in synaptogenesis, neurotransmission and neurogenesis, which gives them the ability to control the activity of neuronal circuits. Moreover, they regulate blood brain barrier permeability and can sense metabolic circulating signals from the periphery supporting their role in neuroendocrine functions. As the main immune cells in the brain, they can perform phagocytosis, antigen presentation, and secrete chemoattractant substances and inflammatory mediators in response to injury and infection.

Dysfunction of microglia and/or modification of the microglial homeostatic state clearly contributes to the pathogenesis of some brain diseases (Sirkis et al., 2021; Paolicelli et al., 2022), including peroxisomal leukodystrophies such as X-linked adrenoleukodystrophy (X-ALD, MIM 300100). X-ALD, the most frequent peroxisomal disorder, is associated with mutations in the *ABCD1* gene and is characterized by an impaired peroxisomal β-oxidation pathway and very long-chain fatty acid (VLCFA) accumulation in plasma and tissues of patients (Mosser et al., 1993; Trompier and Savary, 2013; Kemp et al., 2016). Functional replacement of microglia by monocyte/macrophage cells using allogeneic hematopoietic stem cell transplantation and cell-based gene therapy has proven to be therapeutically effective for X-ALD patients (Cartier et al., 2009; Eichler et al., 2017; Weinhofer et al., 2018). Metabolic defects in microglia are indeed suggested to be key contributors to the pathology of X-ALD. Moreover, it has also been proposed that a primary microglial dysfunction might directly initiate neurodegenerative processes since microglial alteration precedes myelin breakdown in X-ALD (Gong et al., 2017; Bergner et al., 2019).

Using the CRISPR/Cas9 gene editing technology, we recently established novel cell models to study the impact of peroxisomal defects in BV-2 murine microglial cells (Raas et al., 2019a,b). The BV-2 cells were chosen because they maintain transcriptomic signature resembling that of primary cells and many microglial features such as phagocytosis and the ability to respond to inflammatory stimulation (Henn et al., 2009; Das et al., 2015). Despite thousands of articles using BV-2 cells as a model, the cell line is not fully representative of primary cells especially upon LPS treatment with a lower reactivity than what is observed with primary cells (Das et al., 2016). Moreover, primary microglia present a spatial and temporal heterogeneity particularly under pathological status (Masuda et al., 2019). While fully aware of the limitations of the model, we decided to use the BV-2 cell line and targeted the *Abcd1* and *Abcd2* genes, which encode for partially redundant peroxisomal ABC transporters of CoA esters of VLCFAs (Mosser et al., 1993; Lombard-Platet et al., 1996; Genin et al., 2011; Tawbeh et al., 2021), and the Acyl-CoA oxidase 1 (*Acox1*) gene, which controls the first step of peroxisomal β-oxidation and whose defect is associated with a rare leukodystrophy (MIM 264470; Fournier et al., 1994; Nohammer et al., 2000; Ferdinandusse et al., 2007; Vamecq et al., 2018). Peroxisomal ABC transporters show overlap in their substrate specificity, with ABCD1 being more specialized in the transport of saturated and monounsaturated VLCFAs while ABCD2 would show an extended specificity to polyunsaturated fatty acids (PUFAs; Mosser et al., 1993; Lombard-Platet et al., 1996; Genin et al., 2011; Tawbeh et al., 2021). Both transporters provide the substrates of ACOX1 for further degradation. Since BV-2 cells express both *Abcd1* and *Abcd2* genes, VLCFA accumulation was only observed in the *Abcd1^−/−^Abcd2^−/−^* and *Acox1^−/−^* microglial cells, as expected (Raas et al., 2019a,b). Lipid droplets and lipid inclusions likely due to cholesteryl esters of VLCFA, some of the hallmarks of X-ALD, were also observed, especially in the *Abcd1^−/−^Abcd2^−/−^* genotype (Raas et al., 2019a,b).

To further clarify how peroxisomal defects impact microglia, we used next generation sequencing (NGS)-based approach to obtain an accurate and unbiased vision of the differentially expressed genes (DEGs) in microglial cells with specific peroxisomal gene mutations. RNA-sequencing (RNA-seq) indicated the induction of specific transcriptomic programs in the mutant genotypes. Nevertheless, intersectional analysis revealed a common transcriptomic program in the four genetic mutants. Gene ontology analysis permitted to highlight deregulated gene clusters involved in immune system process, signaling pathways, lipid metabolism, lysosome, and autophagy. We also demonstrated the induction of a disease-associated microglial (DAM) signature in microglia cells bearing one or several peroxisomal gene mutations. A DAM signature is a transcriptomic response shared by most common neurodegenerative diseases (Keren-Shaul et al., 2017; Butovsky and Weiner, 2018; Dubbelaar et al., 2018). We confirmed these transcriptional modifications at the protein level for several dysregulated genes and explored some functional consequences on plasma membrane (cholesterol accumulation), lysosomal alterations and autophagy. Moreover, we observed an increased secretion of five selected proteins belonging to the DAM signature, which prompted us to launch a pilot study in X-ALD adult patients.

## Materials and methods

### Resource availability

The data discussed in this publication have been deposited in NCBI’s Gene Expression Omnibus (Edgar et al., 2002) and are accessible through GEO Series accession number GSE200022.^1^

The data supporting the findings and the excel files containing the gene lists used to create the figures are available from the corresponding author (SS) upon request.

### Cell culture

Mouse microglial BV-2 cell line was purchased from Banca-Biologica e Cell Factory (catalog no. ATL03001). Single or double mutant BV-2 cells, deficient for the peroxisomal proteins Abcd1, Abcd2 or Acox1 (*Abcd1^−/−^, Abcd2^−/−^, Abcd1^−/−^/Abcd2^−/−^,* and *Acox1^−/−^*), were obtained by CRISPR/Cas9 editing (Raas et al., 2019a,b). Sanger sequencing confirmed the absence of CRISPR/Cas9-induced mutations in the two genomic sites with the highest predicted likelihood of off-target binding strongly, suggesting the absence of off-target events. WT and mutant BV-2 cells were grown in DMEM supplemented with 10% heat-inactivated FBS (Corning), 100 U/mL penicillin and 100 μg/mL streptomycin (Gibco). Cultures were maintained at 37°C in a humidified atmosphere containing 5% CO_2_. For RNA-seq experiments, as BV-2 WT and mutant cells were included as controls in a study involving treatments (study which will be the subject of another article), a treatment with 0.01% ethanol and 0.5 mM α-cyclodextrin corresponding to the vehicle was applied.

### Blood and plasma samples

Frozen plasma samples from male X-ALD patients were obtained from the X-ALD Biobank of Dijon (agreement for research usage CRB.FC-MAD 12236.4, Centre de Ressources Biologiques (CRB) Ferdinand Cabanne, Dijon, France). There is no restriction on the use of these human samples to identify putative biomarkers. Control plasma samples were obtained after centrifugation (1,300 g for 10 min) from fresh blood samples obtained from male healthy donors (agreement for the transfer of blood products for non-therapeutic use between Etablissement français du sang Bourgogne Franche-Comté, France and University of Bourgogne DECO-21-0051). Analyzes were carried out in samples from 3 patients with arrested cALD (ages 36, 16, and 30 years, with onset ages of 21, 10, and 14 years, respectively for patients cALD1, cALD2 and cALD3), 3 AMN patients (ages 43, 42, and 44 years, with onset ages of 33, 25, and 36 years, respectively for AMN1, AMN2, and AMN3), and 10 healthy donors aged 40 to 45 years. Last examination of cALD patients revealed a severe dementia (cALD1), a mildly abnormal gait and moderate dementia (cALD2) and a near-normal situation (cALD3). Their C26:0 plasma level at the date of sampling was 2.40 μmol/L (cALD1), 3.39 μmol/L (cALD2), 5.96 μmol/L (cALD3; normal value < 1.45 μmol/L). Patients cALD1 and 2 received Lorenzo’s oil.

### RNA-sequencing

Total RNA was extracted from 3 independent batches of BV-2 cells for each genotype (WT, *Abcd1^−/−^, Abcd2^−/−^, Abcd1^−/−^Abcd2^−/−^,* and *Acox1^−/−^*) using RNeasy kit (Qiagen). RNA sequencing libraries were prepared using the Illumina TruSeq Stranded mRNA LT Sample Preparation Kit and sequencing was performed on Illumina HiSeq4000 sequencer as single-end 1×50 base reads. Reads were preprocessed using cutadapt (Martin, 2011) version 1.10 in order to remove adapter and low-quality sequences (Phred quality score below 20) and reads shorter than 40 bases were discarded for further analysis. Reads mapping to rRNA sequences were also discarded [this mapping was performed using bowtie (Langmead and Salzberg, 2012) version 2.2.8]. Reads were then mapped onto the mm10 assembly of Mus musculus genome using STAR (Dobin et al., 2013) version 2.5.3a. Gene expression was quantified using htseq-count (Anders et al., 2015) version 0.6.1p1 and annotations from Ensembl version 91. Differential gene expression analyses were performed using R 3.3.2 and DESeq2 version 1.16.1 Bioconductor library (Love et al., 2014). *p* values were adjusted for multiple testing using the Benjamini and Hochberg method (Benjamini and Hochberg, 1995).

### Differential gene expression analysis and bioinformatic

Venn diagrams were obtained using Venny 2.1.^2^ MA-Plot and Volcano plot were obtained using the VolcaNoseR web app.^3^ Gene ontology analysis and gene set enrichment analysis were performed using the DAVID resources^4^ (Huang et al., 2007; Huang da et al., 2009) for association with KEGG pathways and the Enrichr platform^5^ (Kuleshov et al., 2016) for association with Wikipathways 2019 Mouse and Elsevier pathway collection. Significance level of 0.05 was used when not correcting for multiple testing. A cutoff of 0.05 for adjusted *p-*value was chosen for multiple testing correction using the method from Benjamini and Hochberg (BH). DEGs were selected from genes presenting an adjusted *p*-value lower than 0.05 and an absolute log2 FC higher than 0.58. Expression-based heatmaps and hierarchical clustering were performed with Heatmapper^6^ (Babicki et al., 2016) using average linking and Pearson distances.

### Lipid analysis

The levels of cholesterol precursors were measured by gas chromatography–mass spectrometry (GC–MS) from cellular homogenates, prepared from pellets of 10^7^ cells suspended in water (100 μL) and mixed with structural homologous internal standards as previously described (Raas et al., 2019a). Peak integration was performed manually. Metabolites were recognized by retention time and fragmentation patterns, and quantified from total-ion count against internal standards using standard curves for the measured sterols (Leoni et al., 2017).

### Western blotting

Cell lysate proteins (30 μg) were separated by SDS-PAGE and transferred to PVDF membranes. Membranes were first blocked in 5% fat-free milk in PBST (Phosphate buffer saline (PBS), 0.1% Tween 20) and then probed with primary antibodies in 1% fat-free milk in PBST. The following antibodies were used with the indicated dilution: anti-APOE (1:2,000, Abcam ab183597), anti-ATG13 (1:1,000, Abcam ab201467), anti-ATP6V1B2 (1 μg/mL, Abcam ab73404), anti-ATP6V0D2 (1:1,000, Sigma-Merck SAB2103221), anti-CTSB (1:500, Sigma-Merck IM27L), anti-CTSK (1:1,000, Santa Cruz sc-48353), anti-CD36 (1:1,000, R&D Systems AF2519), anti-DHCR24 (1:500, Cell Signaling 2033), anti-GPNMB (1:5,000, Abcam ab188222), anti-GRN (1:1,000, Abcam ab187070), anti-LAMP-2 (1:250, Santa Cruz sc-20,004), anti-LAMTOR4 (1:500, Cell Signaling 12,284), anti-LC3 (1:1,000, Sigma-Merck L8918), LGALS3 (1:1,000, Abcam ab190167), anti-mTOR (1:1,000, Cell Signaling 2,983), anti-p-mTOR(Ser2448; 1:1,000, Cell Signaling 5,536), anti-P62 (1:1,000, Abcam ab56416), anti-SPP1 (1:3,000, R&D Systems AF808), Ulk1 (1:1,000, Cell Signaling 8054) and p-Ulk1(Ser757; 1:1,000, Cell Signaling 14,202). Membranes were washed in PBST and incubated with the appropriate HRP-conjugated secondary antibodies (1,5,000) in 1% fat-free milk in PBST. After 5 washes, immunoreactivity was revealed by incubating membranes with the HRP SuperSignal West Femto Maximum Sensitivity Substrate (ThermoFisher Scientific) and the signal was detected by the Chemidoc XRS system (Bio-Rad). Densitometric analysis was performed using the Image Lab software (Bio-Rad) on membranes obtained from 3 biological replicates for each genotype. Proteins amount loading control and normalization was achieved by probing the membranes with α-tubulin (1,4,000, Sigma-Merck T5168) or β-actin (1,10,000, Sigma-Merck A5441) antibodies.

### Cytometry

BV2 WT and mutant cells were recovered by trypsinization and resuspended in DMEM-Ham F-12 (1:1) supplemented with 1% Nutridoma–SP (Roche 11011375,001; lipid free synthetic serum complement) at a concentration of 10^6^ cells/mL. Filipin III (Merck F4767) binding kinetics (256 s) were performed on a LSR II UV flow cytometer (Becton Dickinson) equipped with a 355 nm UV laser line. Emitted fluorescence was filtered by a 450 nm+/−25 nm dichroic mirror. Filipin III (final concentration of 1 μg/mL) was added to the cells after a 60 s-recording of the natural fluorescence baseline. Cells were maintained in a 37°C water bath throughout the experiment.

### Electron microscopy

Transmission electron microscopy was used to visualize WT and mutant BV-2 cells cultured for 24 h. Cell sample preparation and diaminobenzidine staining to visualize peroxisomes were performed as previously described (Raas et al., 2019a).

### ELISA

BV2 cells were cultured in 6-well plates for 48 h. Cell culture supernatants were subjected to enzyme-linked immunosorbent assay (ELISA) using commercially available ELISA kits to detect cathepsin b (CTSB; Abcam ab119585), osteoactivin (GPNMB; Invitrogen EM59RB), progranulin (GRN; Invitrogen EMGRN), galectin-3 (LGALS3; Invitrogen EMLGALS3) and osteopontin (SPP1; Invitrogen EMSPP1) according to manufacturers’ instructions. Samples were diluted to meet the concentration range of the standard curve. At least three biological replicates for each sample were tested in triplicates and each experiment was repeated at least two times. Osteopontin was quantified in plasma samples (control, AMN, cALD) using Human osteopontin ELISA kit (Invitrogen BMS2066) according to manufacturers’ instructions. Measurements were performed from 1:50 dilution of plasma samples in triplicates and the experiment was repeated twice. Absorbance measurements were performed on TECAN M200 Infinite Pro microplate reader.

### Statistical analyses

The different statistical tests which were applied for densitometric analyses of western blotting experiments and ELISA quantifications are indicated in the figure legends.

## Results

### Genotype comparison

**Figure 1:**
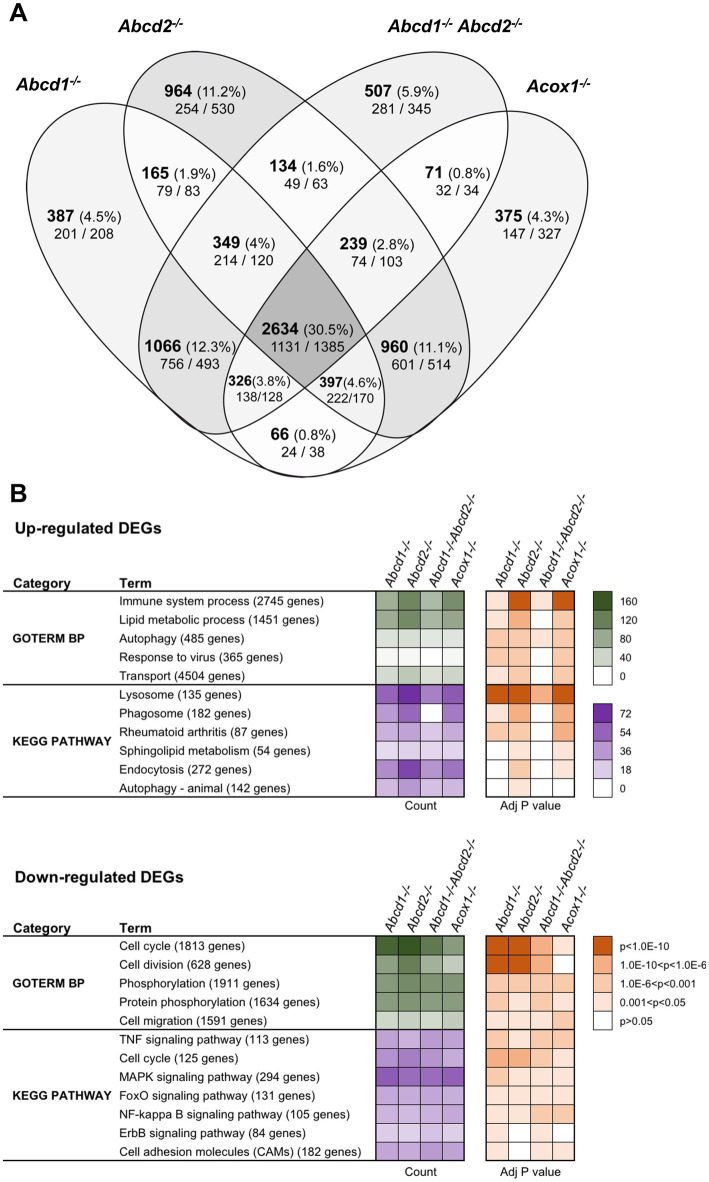
Differentially expressed genes (DEGs) between mutant and WT cells. **(A)** Comparative analysis of the sets of DEGs using Venn diagram (*n* = 3 for each genotype). The significant genes (number and percentage) were selected using a cut off adjusted *p*-value (DESeq2 Wald test with Benjamini and Hochberg *p*-value adjustment) lower than 0.05 and an absolute log2 FC higher than 0.58 (higher than 1.5-fold change in both directions) and are indicated in each group [*Abcd1^−/−^* (5,390 DEGs: 2765 up, 2,625 down), *Abcd2^−/−^* (5,842 DEGs: 2894 up, 2,948 down), *Abcd1^−/−^Abcd2^−/−^* (5,326 DEGs: 2675 up, 2,651 down), and *Acox1^−/−^* (5,068 DEGs, 2,369 up, 2,699 down)]. Numbers below correspond to the genes found in each group when the analysis was performed exclusively on upregulated genes (left) or downregulated genes (right). **(B)** Gene set enrichment analysis in mutant BV-2 cells presenting a selection of the main gene ontology biological process and KEGG pathway terms found in the 4 mutant genotypes. Functional annotation was obtained using DAVID online tools from upregulated and downregulated differentially expressed genes, respectively, in each mutant genotype. Gene count (green for GO Biological process and magenta for KEGG pathway) and Benjamini-corrected *p*-values are mentioned in color-coded form and available in a source data file. The number of genes contained in each term is indicated.

Using a cut off adjusted *p*-value lower than 0.05 and an absolute log2 fold change (log2 FC) higher than 0.58 (1.5-fold induction or repression), we found more than 5,000 genes significantly and differentially expressed for each genotype as compared to the WT cells (Figure 1A). Of these DEGs, a total of 2,634 genes representing about one-third of the total DEGs were shared by each mutant genotype. In the 200 most expressed genes in WT cells, 27 were found differentially expressed in the 4 mutant cell lines. Of note, most of these genes were found associated either with lysosomes (induction of *Ctsb*, *Ctsk*, *Ctsz*, *Lamp1*, *Atp6v1b2*, *Atp6v1c1*, *Grn*, *Psap*) or with the DAM signature (induction of *Tyrobp* (DAP12), *ApoE*, *Gpnmb*, *Spp1*, *Grn*, *Lgals3* and repression of *Isyna1*). Analysis performed exclusively on the upregulated genes revealed 1,131 genes shared by all mutant cells. Counterpart analysis on the repressed genes revealed 1,385 DEGs.

Single genotypes analysis revealed unique gene expression profile for each genotype. The comparison of genotypes two by two surprisingly indicates a greater overlapping between *Abcd2^−/−^* and *Acox1^−/−^* cells with 4,230 intersecting DEGs comprising 960 genes DEGs exclusive to these two genotypes (Figure 1A). When we compared the transcriptional profiles of *Abcd1^−/−^* and *Abcd1^−/−^Abcd2^−/−^* cells, we observed 4,105 intersecting DEGs including 1,066 DEGs exclusive to these two genotypes. When we look at the transcriptional profiles of mutant cells presenting VLCFA accumulation and supposed to have the most important cellular dysfunction, i.e., *Abcd1^−/−^Abcd2^−/−^* and *Acox1^−/−^* (Raas et al., 2019a,b), they showed 3,270 DEGs including 3,025 genes differentially expressed in the same direction (1,375 upregulated and 1,650 downregulated) and 245 genes with a mirror regulation. Only 71 genes were found differentially expressed exclusively in these two genotypes (Figure 1A).

To characterize the enriched biological processes and pathways in our screen, the DEGs were subjected to a functional enrichment analysis using the DAVID online toolkit. A selection of the most enriched terms with Benjamini adjusted *p*-values (false discovery rate) below 0.05 is presented in Figure 1B. From the gene set enrichment analysis restricted to upregulated genes in mutant cells compared to WT cells, “Immune system process” (GO:0002376) was the most significant enriched GO terms in the four deficient genotypes and was followed by “Lipid metabolic process” (GO:0006629) and “Autophagy” (GO:0006914). When analyzing downregulated DEGs, “cell cycle” (GO:0007049), “Cell division” (GO:0051301), and “Phosphorylation” (GO:0016310) were found most enriched. Enrichment analysis restricted either to upregulated or downregulated genes uncovered significant involvement in Kyoto Encyclopedia of Genes and Genomes (KEGG) pathways (Figure 1B). From the analysis of the upregulated gene sets, “Lysosome” (mmu04142), “Phagosome” (mmu04145), and “Autophagy - animal” (mmu04140) ranked first. Interestingly, “Phagosome” was not found in the *Abcd1^−/−^Abcd2^−/−^* cells illustrating genotype specificities. On the other hand, the analysis of the downregulated DEGs demonstrated a significant enrichment in many signaling pathways related to inflammation control (Figure 1B).

We extended our analysis to Wikipathways 2019 Mouse and Elsevier Pathway collection in order to perform statistical analyses that highlight the biological processes significantly altered as a consequence of gene expression changes. From the 1,131 upregulated genes found in the intersection of the 4 mutant genotypes, “TYROBP causal network” (WP3625; Zhang B. et al., 2013) was the first and most significantly enriched pathway (Adjusted *p*-value = 0.0101). From the repressed DEGs and gene set enrichment analysis using the Elsevier Pathway collection, “Lipoxins and Resolvins in Inflammation Resolution” (Adjusted *p*-value = 0.0253) was the most significant hit.

### Lipid related genes

Peroxisomal defects *in cellulo* and *in vivo* are known to affect lipid metabolism in an extensive manner (Lodhi and Semenkovich, 2014). With their ability to interact with diverse organelles, peroxisomes affect both anabolic and catabolic pathways. It leads to the accumulation or defects of various lipid metabolites, which serve as metabolic precursors, signaling molecules impacting membrane and cellular functions, and regulators of transcription factors including nuclear receptors. In the BV-2 microglial cell models, saturated VLCFA accumulation was observed in the *Abcd1*^−/−^*Abcd2*^−/−^ cells and *Acox1*^−/−^ mutants along with increase in lipid droplets (Raas et al., 2019a,b). Depending on the genotypes, we noticed some modifications of the levels of monounsaturated and polyunsaturated long-chain fatty acids. In addition, we observed cholesterol accumulation in the *Abcd1*^−/−^ and *Abcd1*^−/−^*Abcd2*^−/−^ cells as well as modifications of oxysterols levels. Whorled lipid inclusions, which likely result from the accumulation of cholesteryl esters of VLCFA, were observed in the *Abcd1*^−/−^*Abcd2*^−/−^ cells. The high rank of the GO term “Lipid metabolic process” in our functional enrichment analysis is in agreement with the observed modifications impacting lipids and reflects a wide reprogramming of lipid metabolism.

**Figure 2:**
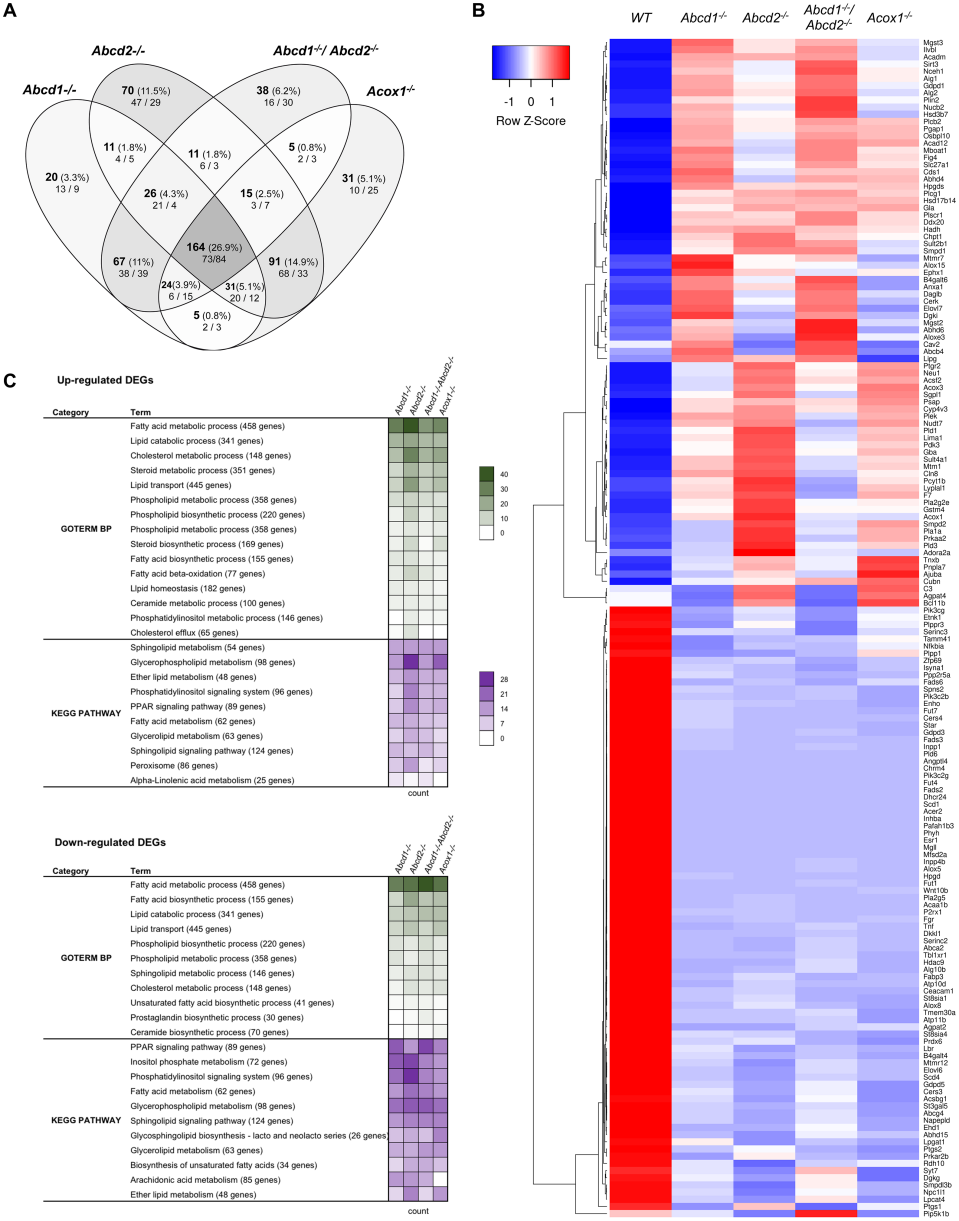
Reprogramming of lipid metabolism in mutant cells. **(A)** Comparative analysis of the sets of DEGs using Venn diagram (*n* = 3 for each genotype). From the 1,190 genes associated with the GO term “Lipid metabolic process” and expressed in BV-2 cells, the significant genes (number and percentage) were selected using a cut off adjusted *p*-value (DESeq2 Wald test with Benjamini and Hochberg *p*-value adjustment) lower than 0.05 and an absolute log2 FC higher than 0.58 (higher than 1.5-fold change in both directions) and are indicated in each group [*Abcd1^−/−^* (348 DEGs: 177 up, 171 down), *Abcd2^−/−^* (419 DEGs: 242 up, 177 down), *Abcd1^−/−^Abcd2^−/−^* (350 DEGs: 165 up, 185 down), and *Acox1^−/−^* (366 DEGs: 184 up, 182 down)]. Numbers below correspond to the genes found in each group when the analysis was performed exclusively on upregulated genes (left) or downregulated genes (right). **(B)** Expression-based heatmap showing hierarchical clustering (left) using average linking and Pearson distances based on the 164 DEGs of the intersection between the 4 mutant genotypes (right). Each point corresponds to the Row Z-score collected from the means of the reads normalized and divided by median of transcripts length in kb from 3 independent cell samples for each genotype. **(C)** Biological processes and gene ontology pathways related to lipid metabolism corresponding to over and under-expressed genes in mutant BV-2 cells. Functional annotation was obtained using DAVID online tools from upregulated and downregulated DEGs in each mutant genotype. A selection of the most represented terms is presented with their number of associated genes mentioned in color-coded form (green for GO Biological process and magenta for KEGG pathway) and available in a source data file. The number of genes contained in each term is indicated.

From the 1,190 genes associated with lipid metabolism and expressed in BV-2 cells, a total of 609 genes were found differentially expressed with the chosen cut off (348 for *Abcd1*^−/−^, 419 for *Abcd2*^−/−^, 350 for *Abcd1*^−/−^*Abcd2*^−/−^, and 366 for *Acox1*^−/−^ cells; Figure 2A). Like in the global analysis, Venn diagram demonstrated that the most important group of DEGs (164 genes) belong to the intersection of the 4 mutant genotypes (Figure 2A). The heatmap analysis of these genes indicates a greater overlapping between *Abcd2*^−/−^ and *Acox1*^−/−^ cells with 91 DEGs in common, and between *Abcd1*^−/−^ and *Abcd1*^−/−^*Abcd2*^−/−^ cells with 67 shared DEGs (Figure 2B). MA Plot showed that hits concern both low- and high-expressed genes (e.g., *ApoE*) while Volcano plot illustrated an equilibrated repartition of DEGs with the most significantly and differentially expressed genes being downregulated (Supplementary Figure 1). A majority of the genes belonging to these pathways were indeed moderately induced or repressed but several genes such as *Fads2*, *Scd1*, *Acer2*, *Dhcr24* were found drastically downregulated in every mutant genotype. GO and KEGG pathway enrichment demonstrated an overall modification of the expression of genes associated with lipid metabolism widely impacting fatty acids catabolism and biosynthesis, membrane lipid metabolism, lipid transport, cholesterol metabolism, ether-lipid metabolism, and lipid-related signaling pathways (Figure 2C).

#### Fatty acid metabolism

We found many genes belonging to both fatty acid biosynthesis and oxidation pathways (Figures 2B,C; Supplementary Figure 1). Both peroxisomal and mitochondrial catabolic pathways were impacted. Among the peroxisomal β-oxidation genes, the most striking observation was a strong repression of *Acaa1b* (3-ketoacyl-CoA thiolase B). Genes controlling mitochondrial β-oxidation *Cpt2* (carnitine palmitoyltransferase 2)*, Acads* and *Acadm* (short and medium fatty acyl-CoA dehydrogenase encoding genes respectively), *Hadh* (hydroxyacyl-CoA dehydrogenase, which plays an essential role in the mitochondrial β-oxidation of short chain FA) were also among the hits (Figure 2B; Supplementary Figure 1). Since VLCFA accumulation is not exclusively related to the β-oxidation defect and may also be associated with an increase in *de novo* synthesis, we looked at the expression of the *Elovl* genes encoding FA elongases (Jump, 2009; Kihara, 2012). *Elovl6* (elongation of saturated and monounsaturated fatty acids C16:0-CoA or shorter) was found repressed in all mutant cells while *Elovl4* (elongation of extremely long saturated FAs and PUFAs > C26) and *Elovl7* (elongation of C16–C20-CoA) were induced except in *Acox1*^−/−^ cells. *Elovl7* was among the most significantly upregulated DEGs in both *Abcd1*^−/−^ and *Abcd1*^−/−^*Abcd2*^−/−^ cells (Figure 2B; Supplementary Figure 1). *Elovl1*, which encodes for the main enzyme responsible for C26:0 synthesis (Ofman et al., 2010), was found very weakly but significantly induced in all mutant genotypes. Moreover, among the four 3-hydroxyacyl-CoA dehydratase encoding genes involved in VLCFA synthesis (Ikeda et al., 2008), *Hacd4* was found upregulated and *Hacd3* weakly repressed in every mutant genotype (Supplementary Figure 1). Concerning the acyl-CoA synthetase encoding genes which participate both in biosynthesis and oxidation pathways, *Acsbg1* was found repressed, especially in *Abcd2^−/−^ and Acox1^−/−^* cells (Figure 2B; Supplementary Figure 1). On the contrary, the genes encoding acyl-CoA synthetase genes for medium-chain (*Acsf2* and *Acsf3*), long-chain (*Acsl1*), and very long-chain (*Slc27a1*), were found upregulated (Figure 2B; Supplementary Figure 1).

Unsaturated fatty acid metabolism was in the enriched pathways of mutant cells in accordance with the observed variations in the levels of these fatty acids (Raas et al., 2019a,b). Four stearoyl-CoA desaturases encoded by *Scd* genes catalyze the rate-limiting step in the production of MUFAs from C16:0 and C18:0. *Scd2*, the most expressed gene (10-fold more than *Scd1*), remained unchanged regardless of the genotype. *Scd1* expression was considerably reduced in every mutant genotype (Figure 2B; Supplementary Figure 1). *Scd3* and *Scd4*, although poorly expressed in BV-2 cells, were also repressed in every mutant genotype (Figure 2B; Supplementary Figure 1). The high expression level of *Scd2* and the substrate specificity shared with *Scd1* may explain why total MUFA levels have remained almost unchanged. Concerning PUFAs, the levels of AA and DHA remained unchanged in the *Abcd2*^−/−^ and *Acox1*^−/−^ cells but were increased in *Abcd1*^−/−^ and *Abcd1*^−/−^*Abcd2*^−/−^ cells (Raas et al., 2019a,b). *Fads2* encodes for a Δ6-desaturase which is the rate-limiting enzyme of the long-chain PUFA biosynthesis such as arachidonic acid (AA, C20:4, n-6) and eicosapentaenoic acid (EPA, 20:5, n-3) and which controls the last step of desaturation for docosahexaenoic acid (DHA, 22:6, n-3) and docosapentaenoic acid (DPA, 22:5, n-6) synthesis. Paradoxically, *Fads2* expression was hugely decreased in the 4 mutant cells (Figure 2B; Supplementary Figure 1). *Fads3* and *Fads6* genes, which are weakly expressed, were also found repressed in every mutant genotype while *Fads1* (the gene encoding a Δ5-desaturase forming AA and EPA) remained unchanged (Figure 2B; Supplementary Figure 1). Mobilization of fatty acids or other lipid derivatives from membrane lipids or triacylglycerols represents a very abundant source of signaling molecules which play a major role in the control of inflammation, especially in the brain (Joensuu et al., 2020). A large number of genes encoding lipases and phospholipases was observed among the hits, most of them being upregulated. A few ones such as *Mgll*, an important regulator of neuroinflammation in the brain, were strongly repressed (Figure 2B; Supplementary Figure 1). *Mgll* encodes for the monoglyceride lipase which controls hydrolysis of the endocannabinoid 2-arachidonoylglycerol in the brain generating AA and glycerol (Grabner et al., 2016). Interestingly, the entire pathway leading to produce endocannabinoid arachidonoyl ethanolamine (anandamide), which plays an important role in microglia and brain homeostasis (Tanaka et al., 2020), was found particularly deregulated (induction of *Abhd6, Camk2a*, *Daglb*, *Faah*, and repression of *Mgll*, *Dagla*, *Alox8*, *Napepld*; Figure 2B; Supplementary Figure 1). Since inhibition of FAAH was shown to be neuroprotective (Tanaka et al., 2020), the observed alterations may contribute to neurodegenerative process in peroxisomal disorders.

Many other phospholipase encoding genes were found among the DEGs indicating that the production of several signaling lipids such as diacylglycerol, inositol-trisphosphate, N-acylethanolamine, or phosphatidic acid is likely affected. Among the *Pla2* genes which control the release of unsaturated fatty acids such as AA, a precursor of inflammatory mediators, we observed a repression of *Pla2g4c* and *Pla2g5*, as well as an induction of *Pla2g2e* in every mutant genotype (Figure 2B; Supplementary Figure 1). *Pla2g2d* and *Pla2g7* were induced in *Abcd1*^−/−^, *Abcd2*^−/−^, and *Abcd1*^−/−^*Abcd2*^−/−^ cells (Supplementary Figure 1). Interestingly, genes involved in AA or DHA metabolism were found deregulated (induction of *Aloxe3* and *Alox15*, repression of *Alox5* and *Alox8*; Figure 2B; Supplementary Figure 1). Among the genes associated with leukotriene metabolism, *Ltc4s, Mgts2 and Mgts3* were induced while *Lta4h* remained unchanged. Genes involved in the synthesis of the prostaglandins were also among the hits. Finally, *Tbxa1s*, which is responsible for thromboxane synthesis was repressed in *Abcd1*^−/−^ and *Abcd1*^−/−^*Abcd2*^−/−^ cells (Supplementary Figure 1). In summary, our transcriptional analysis suggests a large modification of the metabolic pathways controlling the levels of pro-inflammatory eicosanoids (leukotrienes and prostaglandins) and anti-inflammatory docosanoids (resolvins) in the mutant microglial cells.

#### Lipid droplets

Lipid droplets play a major role in fatty acid homeostasis and participate actively to the inter-organelle exchanges associated with lipids (Renne and Hariri, 2021). From the preliminary observations in the BV-2 mutant cells showing lipid droplets and lipid inclusions (Raas et al., 2019a,b), modification of the transcriptome associated with lipid droplets was expected. About 40 genes related to lipid droplets were indeed found differentially expressed, a majority being upregulated. Lipid droplets are now considered to be highly dynamic organelles consisting of a core of triglycerides and cholesteryl-esters surrounded by a monolayer of phospholipids and proteins such as perilipins. Among the main actors regulating the generation and the maturation of lipid droplets, we noticed the induction of the perilipin encoding genes, *Plin2* and *Plin3* (Figure 2B; Supplementary Figure 1). The *Rsad2* gene, which encodes for Viperin was the most induced gene of this group. Viperin is localized in the lipid droplets and participates in the antiviral response and in the regulation of lipid synthesis (Hinson and Cresswell, 2009). Concerning the lipid composition of the lipid droplets, the increased labeling of neutral lipids observed by Oil Red O staining in mutant cells (Raas et al., 2019a,b) is likely due to cholesteryl-esters and not triglycerides. The *Soat1* and *Soat2* genes encoding sterol-O-acyltransferase-2 that produces cholesteryl esters from cholesterol and LCFA or VLCFA were indeed upregulated. Interestingly, *Bscl2*, which encodes for Seipin, was found significantly repressed but only in *Abcd1*^−/−^ and *Abcd1*^−/−^*Abcd2*^−/−^ cells and could be associated to the ultrastructure differences concerning lipid droplets observed in the mutant cells (Supplementary Figure 1). Seipin was indeed shown to facilitate contacts between lipid droplets and endoplasmic reticulum and its deficiency was shown to result in lipid droplet size heterogeneity (Salo et al., 2019).

#### Glycerophospholipids and sphingolipids

Pathway analysis of DEGs revealed a strong representation of genes associated with membrane lipids (glycerophospholipids, plasmalogens and sphingolipids). This was particularly true for upregulated DEGs. *De novo* synthesis of phospholipids from glycerol-3-phosphate to form phosphatidylcholine, phosphatidylethanolamine and phosphatidylinositol and their remodeling consisting of various exchanges of fatty acids are essential for membrane functions (Yamashita et al., 2014). In general, saturated fatty acids are present at the sn-1 position while unsaturated occupy the sn-2 position. Besides their role associated with membrane properties, phospholipids also act as reservoirs for bioactive lipids (eicosanoids, docosanoids, lysophospholipids, anandamides) thanks to the activity of various phospholipases, transacylases or acyltransferases. Many genes of the glycerophospholipid metabolism were found differentially upregulated in the 4 mutant genotypes (Figure 2B; Supplementary Figure 1). We observed a repression of *Agpat2* and *Plpp1* and upregulation of *Cds*1 in the mutant cells, genes associated with the formation of phosphatidic acid (*Agpat2*) and then its conversion to diacylglycerol to yield phosphatidylcholine and phosphatidylethanolamine (*Plpp1*) or cytidine diphosphate diacylglycerol to yield phosphatidylinositol and cardiolipin (*Cds*1). Genes encoding phospholipases D which control conversion of phosphatidylcholine or phosphatidylethanolamine into phosphatidic acid, were induced. Genes encoding diacylglycerol kinases phosphorylating diacylglycerol to phosphatidic acid, were differentially expressed (induction of *Dgki* and repression of *Dgka* and *Dgkg*). The phosphatidylcholine pathway appears to be induced since we observed upregulation of *Pld1*, *Pcyt1b*, *Chpt1*, and *Lpca*t2 (Figure 2B; Supplementary Figure 1). Remodeling of glycerophospholipids and lysophospholipids is also apparently widely affected and differs between the genotypes. For instance, we observed the induction of and *Cept1* in *Abcd2*^−/−^ and *Acox1*^−/−^ cells. Conversely *Agpat4* was repressed in *Abcd1*^−/−^ and *Abcd1*^−/−^*Abcd2*^−/−^ cells. Many other acyltransferase encoding genes and phospholipid phosphatase encoding genes figured in the DEGs (Supplementary Figure 1).

Sphingolipid metabolism and sphingolipid catabolic processes came out on a high rank in the sorting of gene ontology (Figure 2C). Sphingolipid metabolism has progressively received attention in neurodegenerative diseases and is now considered as a key component of neuroinflammation regulation (Assi et al., 2013; Lee et al., 2020). We observed that a majority of genes related to sphingolipid metabolism were significantly upregulated in the mutant cells (Figure 2B; Supplementary Figure 1). A few genes were downregulated and among them, *Acer2,* which controls the hydrolysis of long- and very long-chain ceramides and dihydroceramides to yield sphingosine, and *Cers4,* the ceramide synthase 4 encoding gene (Figure 2B; Supplementary Figure 1). It is noteworthy that the most expressed ceramide synthase encoding genes in BV-2 cells (*Cers2*, *Cers5*, *Cers6*) were not differentially expressed. Some of these results (induction of *Smpd1*, *Cerk*, *Sphk1*, and repression of *Acer2*, *Cers3*, *Cers4*) are in agreement with the transcriptomic data obtained from patient fibroblasts with cerebral or adrenomyeloneuropathy forms of X-ALD showing that the anabolic pathway toward sphingolipids and glycosphingolipids is downregulated (Lee et al., 2019). Concerning glycosphingolipid biosynthesis, many genes belonging to this pathway were repressed and *Fut4*, which is involved in the synthesis of lactosphingolipids, was among the most downregulated genes (Figure 2B; Supplementary Figure 1).

#### Cholesterol

Chronic neurodegenerative disorders are increasingly thought to be associated with dysregulation of cholesterol homeostasis in the brain. Enrichment analysis revealed many genes participating to cholesterol metabolism, transport and homeostasis. *Trem2*, which was found induced in all mutant genotypes, was recently described as a key transcriptional regulator of cholesterol transport and metabolism (Nugent et al., 2020). The expression level of genes encoding the main transcriptional regulators (*Srebf1* and *Srebf2*) was found unchanged. However, a significant but moderate upregulation (Log2FC varying between 0.42 and 0.62 depending on genotypes) of *Insig2* was observed in all mutants (*Insig1* remaining stable) while *Scap* was repressed in *Abcd2*^−/−^ and *Acox1*^−/−^ cells. In the presence of elevated levels of cholesterol, *Insig2* is known to bind SCAP and prevent the proteolytic processing of SREBPs, thereby inhibiting cholesterol synthesis. *Nr1h3*, which encodes for the oxysterol nuclear receptor LXRα, was also induced. *Hdac9*, which was shown to repress cholesterol efflux (Cao et al., 2014) was strongly downregulated, especially in *Abcd2*^−/−^ and *Acox1*^−/−^ cells. *Nfkbia*, which is associated with positive regulation of cholesterol efflux was also repressed. This observation is consistent with the discrepancy found between mutant cells showing that cholesterol accumulate selectively in the *Abcd1*^−/−^ and *Abcd1*^−/−^*Abcd2*^−/−^ cells (Raas et al., 2019a,b).

**Figure 3:**
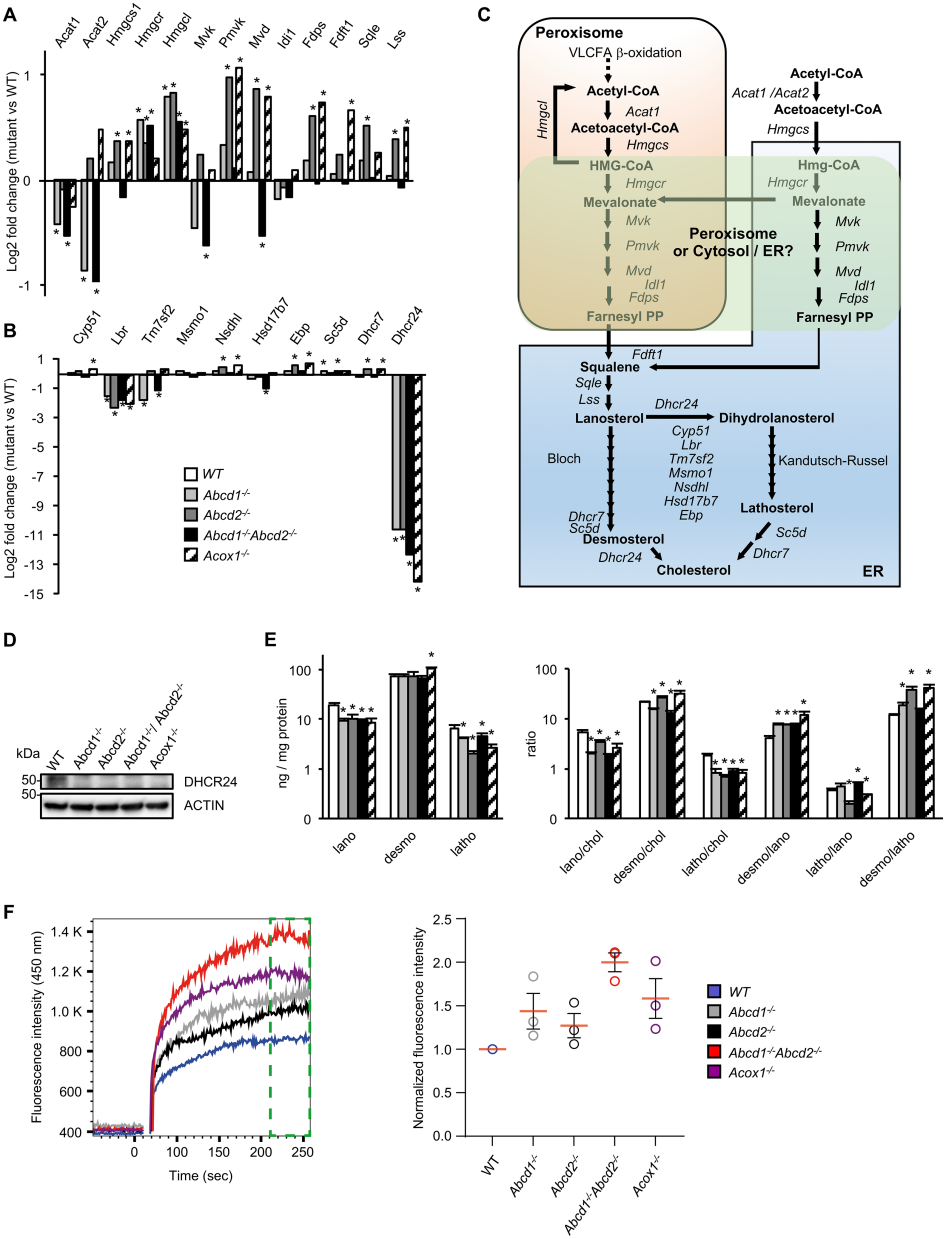
Cholesterol metabolism and membrane cholesterol in mutant cells**. (A)** Differential gene expression between mutant and WT BV-2 cells (n = 3 for each genotype) concerning the genes involved in lanosterol biosynthesis. The histogram represents the log2 FC for each gene based on RNA-seq data. * indicates an adjusted *p*-value lower than 0.05 (DESeq2 Wald test with Benjamini and Hochberg *p*-value adjustment). **(B)** Differential gene expression between mutant and WT BV-2 cells concerning the genes involved in cholesterol biosynthesis from lanosterol (Bloch and Kandutsch-Russell pathways). The histogram represents the estimated log2 FC for each gene based on RNA-seq data. * indicates an adjusted *p*-value lower than 0.05 (DESeq2 Wald test with Benjamini and Hochberg *p*-value adjustment). **(C)** Schematic representation of cholesterol biosynthesis. Enzymatic steps are represented by arrows and the associated genes are indicated. The peroxisomal location of the initial steps of cholesterol biosynthesis remains controversial especially the steps concerning *Hmgcr, Mvk, Pmvk*, and *Mvd* (green windows). **(D)** Western blotting analysis of DHCR4 from cell lysates of WT and mutant BV-2 cells. For densitometric analysis, see Supplementary Figure 3. Source data are available online for this figure. **(E)** Cholesterol precursors levels in WT and mutant BV-2 cells and their ratio. Gas chromatography mass spectrometry (GC–MS) analysis was performed from cellular homogenates (3 independent experiments). Histograms represent the mean levels of cholesterol precursors (ng/mg protein) with their respective standard deviation and their ratios. Statistical significance at *p* < 0.05* determined by Mann–Whitney test analysis is indicated. **(F)** Analysis of cholesterol accumulation in plasma membrane of WT and mutant BV-2 cells. Left panel, Filipin III binding kinetics in living cells recorded by flow cytometry during 256 s. Right panel, median fluorescence intensities calculated from the last minute filipin III binding kinetics (green gate on the left panel) were normalized as a fold of the BV2 WT fluorescence (*n* = 3 independent experiments).

Although many genes of the cholesterol and oxysterol biosynthetic pathways remained quite stable, genes encoding key enzymes of the initial steps of cholesterol synthesis (from acetyl-CoA to lanosterol) were weakly but significantly induced especially in *Abcd2*^−/−^ and *Acox1*^−/−^ cells (Figures 2B, 3A,C; Supplementary Figure 1). The involvement of peroxisomes in these initial steps of cholesterol biosynthesis remains controversial with conflicting reports about peroxisomal location of several enzymes such as HMGCR of MVK for instance (Kovacs et al., 2002; Hogenboom et al., 2004a,b; Figure 3C). In the Bloch and Kandutsch-Russell pathways which drives conversion of lanosterol to cholesterol through several steps of reduction, *Lbr* and *Dhcr24* were found repressed in all mutants while *Tm7sf2* was repressed only in the *Abcd1*^−/−^ and *Abcd1*^−/−^*Abcd2*^−/−^ cells (Figures 3B,C; Supplementary Figure 1). *Nsdhl* and *Ebp* were weakly induced in the *Abcd2*^−/−^ and *Acox1*^−/−^ cells (Figure 3B; Supplementary Figure 1). It is noteworthy that the Kandutsch-Russell pathway (lathosterol pathway) is used for more than 70% of cholesterol biosynthesis in the brain. The expression of *Dhcr24*, which encodes for the 24-dehydroxycholesterol reductase (also called seladin-1) catalyzing the final conversion of desmosterol into cholesterol in the Bloch pathway but also the exchange between the Bloch and Kandutsch-Russell pathways, was found hugely decreased in every mutant genotype (Figures 2B, 3B; Supplementary Figure 1). Western blotting experiments confirmed the loss of expression of *Dhcr24* in mutant BV-2 cells (Figure 3D). Although there was a moderated upregulation of genes of the squalene synthesis pathway, the analysis of the cholesterol precursors revealed an approximately 2-fold decrease in lanosterol content and of the lanosterol/cholesterol ratio in the mutant cells (Figure 3E). The level of lathosterol and the lathosterol/cholesterol ratio were also reduced in the mutant cells suggesting that the Kandutsch-Russell pathway is inhibited. The defect in *DHCR24* is associated with a rare autosomal recessive developmental disorder called desmosterolosis characterized by desmosterol accumulation (Waterham et al., 2001). Surprisingly, the desmosterol level remained quite stable except in the *Acox1*^−/−^ cells (37% increase; Figure 3E). The desmosterol/cholesterol ratio was weakly increased in the *Abcd2*^−/−^ and *Acox1*^−/−^ cells and weakly decreased in the *Abcd1*^−/−^ and *Abcd1*^−/−^*Abcd2*^−/−^ cells in which cholesterol accumulates (Raas et al., 2019a).

Once synthetized, cholesterol can be esterified, oxidized, used for steroidogenesis or transported. As cited above, the sterol-O-acyltransferase encoding genes *Soat1* and *Soat2* were indeed found upregulated. The expression of *Agt*, which positively regulate cholesterol esterification, was also induced (Figure 2B; Supplementary Figure 1). On the other hand, *Nceh1*, which catalyzes the hydrolysis of intracellular cholesteryl-esters, was found induced (Figure 2B; Supplementary Figure 1). Several important genes involved in cholesterol trafficking and lipoprotein metabolism such as *Apoe* and *Apoa2*, were also found upregulated. Interestingly, APOE is the main cholesterol carrier in the brain and was shown to mediate a switch toward a neurodegenerative phenotype in human microglia in association with TREM2 (Krasemann et al., 2017). *Lrp1*, which encodes low density lipoprotein receptor-related protein 1, was found induced (Figure 2B; Supplementary Figure 1). Of note, among the triglyceride lipase encoding genes which contribute to lipoprotein degradation (Loving and Bruce, 2020), *Lipg* was induced except in the *Acox1*^−/−^ cells, *Lipn* was induced except in the *Abcd1*^−/−^*Abcd2*^−/−^ cells, and *Lipa* (lysosomal lipase) was weakly induced in every mutant genotype (Figure 2B; Supplementary Figure 1). Among the ABC transporters associated with cholesterol efflux (Wang et al., 2008), many inductions were observed, suggesting increased efflux of cholesterol but with genotype specificities (Figure 2B; Supplementary Figure 1). Of note, *Npc1* and *Npc2* genes, which are involved in the transport of LDL-derived cholesterol from lysosomes to the cytoplasm (Saha et al., 2020), were induced in *Abcd2*^−/−^ and *Acox1*^−/−^ cells while *Npc1l1*, although weakly expressed, was repressed in every mutant genotype (Figure 2B; Supplementary Figure 1). The loss of these genes triggers endolysosomal accumulation of unesterified cholesterol and lipids and is associated to Niemann–Pick disease (Vance, 2012). In contrast, the expression of *Syt7*, which is involved in the contact between peroxisomes and lysosomes and in the cholesterol transport from lysosomes to peroxisomes (Chu et al., 2015), was decreased in every mutant genotype. *Star*, which transports cholesterol from the outer to the inner mitochondrial membrane and regulates the rate limiting step in steroidogenesis, was repressed in all mutant cells (Figure 2B; Supplementary Figure 1).

To document the consequences of the alterations concerning genes related to cholesterol metabolism and transport, we evaluated the free cholesterol content of the plasma membrane by Filipin III binding kinetics in BV-2 living cells by flow cytometry. We evidenced that Filipin III accumulates more in the plasma membrane of the mutant cell lines compared to BV2 WT cells and in a more pronounced manner in *Abcd1*^−/−^*Abcd2*^−/−^ and *Acox1*^−/−^ cells (Figure 3F). Excess of free cholesterol in the plasma membrane might originate from either a modification of the equilibrium between ordered versus disordered phases affecting lipid membrane organization or modified exchanges between the cellular compartments or enzymatic conversions (Litvinov et al., 2018).

#### Oxysterols

Oxysterol levels were found modified in mutant cells (Raas et al., 2019a) and either oxidative stress or gene regulation may explain this observation. Among the genes involved in oxysterol metabolism (Mutemberezi et al., 2016), only a few genes were deregulated. *Ephx*1, which encodes for cholesterol epoxide hydrolase and forms dihydroxycholesterol, *Hsd3b7*, which converts 7α-hydroxycholesterol to 7α-hydroxy-4-cholesten-3-one, and *Sult2b1*, involved in the formation of sulfoconjugates of 25- and 24-hydroxysterols, were found induced in every mutant genotype (Figure 2B; Supplementary Figure 1). *Sult4a1*, whose function is unknown was also found induced. *Cyp11a1* which encodes for the enzyme catalyzing the synthesis of 22R-hydroxycholesterol was induced in every mutant genotype except *Acox1*^−/−^ cells (Supplementary Figure 1). CYP46A1 converts cholesterol to 24S-hydroxycholesterol, a transport derivative of cholesterol from the brain to the systemic circulation, allowing cholesterol excess elimination in human brain (Petrov and Pikuleva, 2019). Although its expression level seems to be very low in BV-2 cells, *Cyp46A1* was weakly induced in mutant cell lines. The expression level of *Cyp27a1* remained unchanged while that of other main cytochrome P450 genes (*Cyp7a1*, *Cyp7b1*, *Cyp3a4*, *Cyp3a5*, *Cyp39a1*) and other genes (*Ch25h*, *Sult2a1*) involved in oxysterol metabolism were undetectable.

### A “disease-associated microglial signature”-like including upregulation of lysosomal and autophagy-related genes

Lysosome, together with endosome and autophagy, emerged as main terms of gene ontology enrichment analysis (Figure 1B). Moreover, the “Tyrobp (DAP12) causal network” figured in first position of the Wikipathways upregulated in the BV-2 mutant cells. *Trem2* and *Tyrobp* are considered as hub genes for microglial functions and the TREM2-APOE pathway has been shown essential to drive the transcriptional phenotype associated with dysfunctional microglia in neurodegenerative diseases (Jay et al., 2017; Krasemann et al., 2017; Gratuze et al., 2018; Ulland and Colonna, 2018; Deczkowska et al., 2020). *Trem2* encodes a membrane receptor for diverse ligands (LPS, phospholipids, glycolipids, lipoproteins and apolipoproteins) playing an essential physiological role in addition to be central in pathological conditions being responsible to the DAM signature (Butovsky and Weiner, 2018). Indeed, TREM2 recruits DAP12/TYROBP to mediate a complex signaling cascade controlling phagocytosis, cell survival, cell differentiation and inflammation (Konishi and Kiyama, 2018). The three hub genes (*Apoe, Tyrobp, Trem2*) were found significantly induced in the mutant cells (except for *Apoe* in *Acox1*^−/−^ cells). Of note, the expression of *CD36* which encodes for a FA translocase playing an important role in microglial activation (Stuart et al., 2007) and phagocytosis of myelin debris (Grajchen et al., 2020), was found repressed in *Abcd1*^−/−^*Abcd2*^−/−^ cells and induced in *Abcd2*^−/−^ and *Acox1*^−/−^ cells. Microglial dysfunction and lysosomal activity are tightly connected (Gabandé-Rodríguez et al., 2019). In a first step, we therefore focused on lysosome-associated genes and then explored the DEGs in mutant cells with reference to the transcriptomic DAM signature found in microglia in case of neurodegenerative disease.

#### Lysosome and autophagy

**Figure 4:**
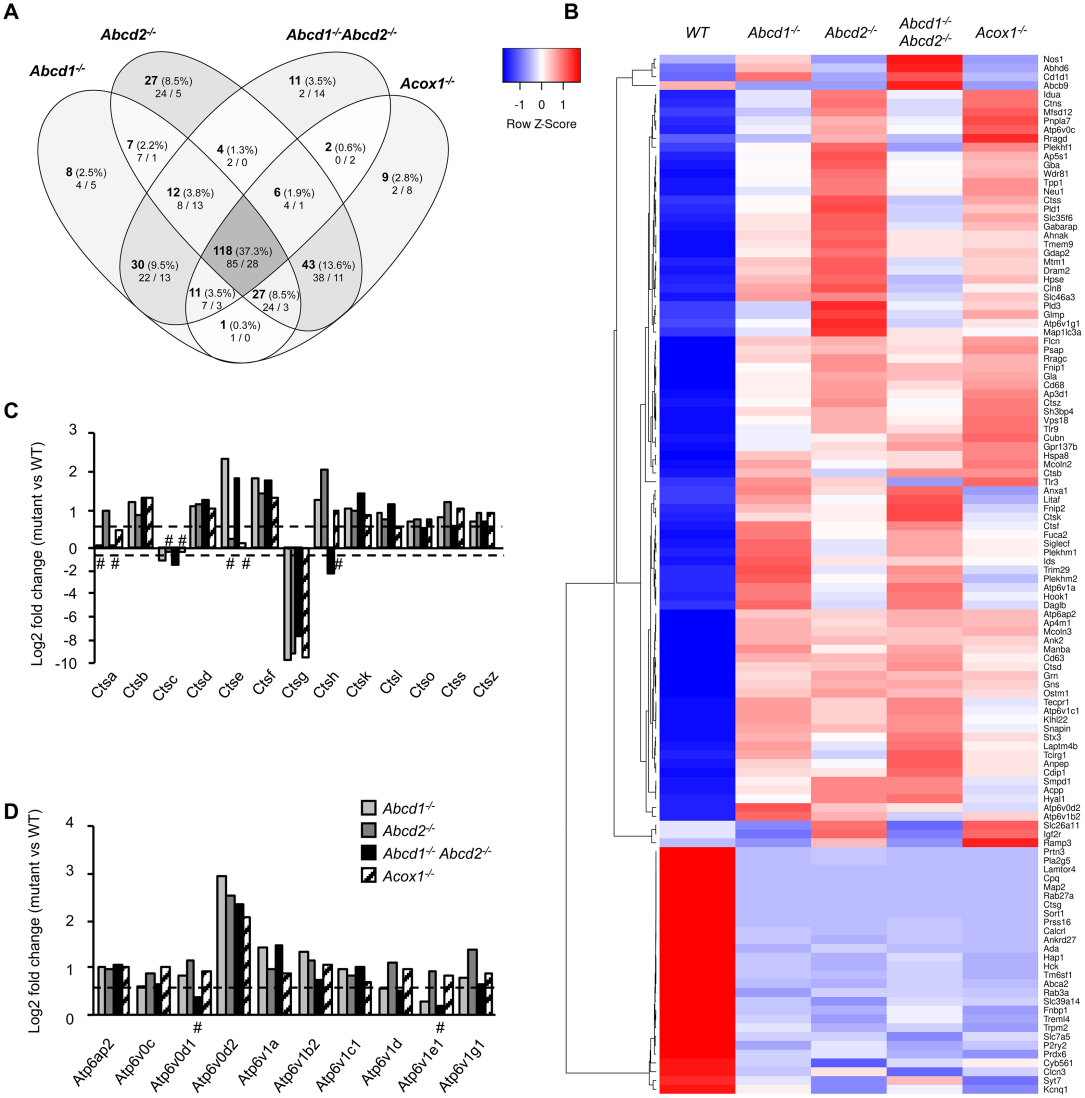
DEGs associated with lysosome and autophagy. **(A)** Comparative analysis of the sets of DEGs using Venn diagram (*n* = 3 for each genotype). From the 513 genes associated with lysosome and expressed in BV-2 cells, the significant genes (number and percentage) were selected using a cut off adjusted *p-*value lower than 0.05 (DESeq2 Wald test with Benjamini and Hochberg *p*-value adjustment) and an absolute log2 FC higher than 0.58 (higher than 1.5-fold change in both directions) and are indicated in each group [*Abcd1^−/−^* (214 DEGs: 158 up, 56 down), *Abcd2^−/−^* (244 DEGs: 192 up, 52 down), *Abcd1^−/−^Abcd2^−/−^* (194 DEGs: 130 up, 64 down), and *Acox1^−/−^* (217 DEGs: 161 up, 156 down)]. Numbers below correspond to the genes found in each group when the analysis was performed only on upregulated genes (left) and downregulated genes (right). **(B)** Expression-based heatmap showing hierarchical clustering (left) using average linking and Pearson distances based on the 118 DEGs of the intersection between the 4 mutant genotypes (right). Each point corresponds to the Row Z-score collected from the means of the reads normalized and divided by median of transcripts length in kb from 3 independent cell samples for each genotype. **(C)** Differential gene expression between mutant and WT BV-2 cells concerning the cathepsin genes. The histogram represents the estimated log2 FC for each gene based on RNA-seq data. For C and D, the bars represent significant differential expression (adjusted *p*-value lower than 0.05) except those indicated by #. **(D)** Differential gene expression between mutant and WT BV-2 cells concerning the V-ATPase genes. The histogram represents the log2 FC for each gene based on RNA-seq data.

**Figure 5:**
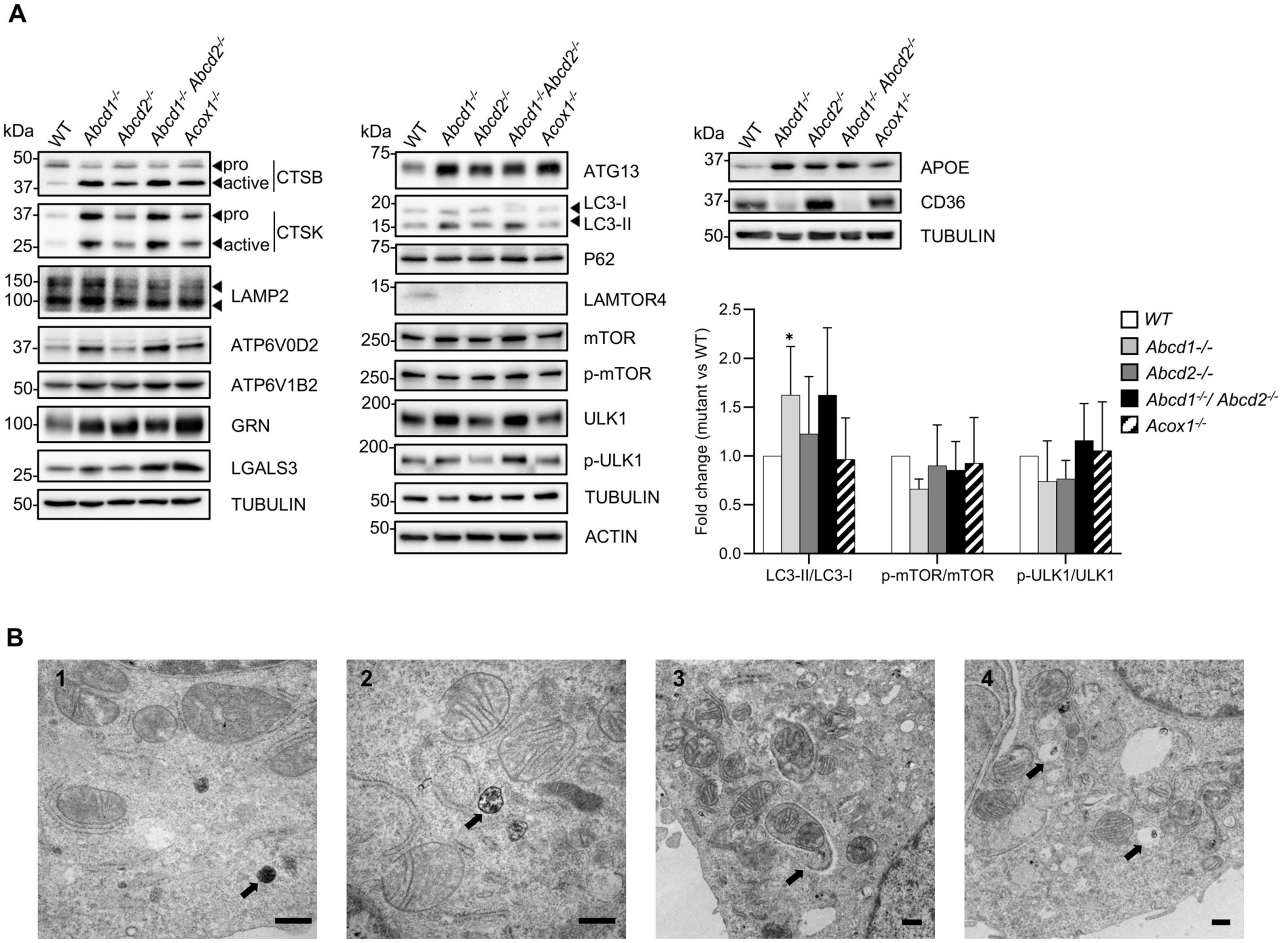
Lysosome and autophagy in mutant cells. **(A)** Western blotting analysis of proteins associated with lysosome [CTSB, CTSK, LAMP2, ATP6V0D2, ATP6V1B2, GRN (Progranulin), LGALS3 (Galectin 3)], autophagy (ATG13, LC3, P62, mMTOR, p-mTOR, ULK1, p-ULK1), lipid metabolism and microglial functions (APOE, CD36) from cell lysates of WT and mutant BV-2 cells. The histogram represents the LC3-II/LC3-I ratio, p-mTOR/mTOR ratio, and p-ULK1/ULK1 ratio obtained from densitometric analysis shown in Supplementary Figure 3. Source data are available online for this figure. **(B)** Transmission electron micrographs of WT (1) and *Abcd1^−/−^Abcd2^−/−^* (2, 3, 4) BV-2 cells illustrating the presence of autophagic figures in the mutant cells (bar = 500 nm). The arrows indicate diaminobenzidine-stained peroxisomes (1), a late autophagic compartment (2), an autophagosome engulfing mitochondria (3) and pexophagy (4). Although less numerous, similar observations were made in the *Abcd1^−/−^* and in the *Acox1^−/−^* cells but not in the *Abcd2^−/−^* cells.

The KEGG term “Lysosome” was the first term to come out from upregulated DEGs genes and from global analysis. From the 513 genes associated with the term “Lysosome” (list combined from KEGG and Gene Ontology search) and expressed in BV-2 cells, a total of 316 were found differentially expressed with a majority of upregulated genes (158 in *Abcd1*^−/−^, 192 in *Abcd2*^−/−^, 130 in *Abcd1*^−/−^*Abcd2*^−/−^, and 161 in *Acox1*^−/−^; Figure 4A; Supplementary Figure 2). Eighty-five genes were found upregulated and 28 downregulated in the 4 mutant genotypes, showing two clustering patterns indicating a higher proximity between *Abcd1*^−/−^ and *Abcd1*^−/−^*Abcd2*^−/−^ and between *Abcd2*^−/−^ and *Acox1*^−/−^ respectively (Figure 4B). Genes involved in metabolism and hydrolysis of various substrates, transport, motility, autophagy, and acidification were found in the hits. Among these genes, *Lamtor4, Map2, Pla2g5, Cpq,* and *Ctsg* emerged as the most repressed genes with log2 FC ratio exceeding −8 (Supplementary Figure 2). Of the most expressed DEGs, *Apoe*, cathepsin encoding genes (*Ctsa, Ctsb, Ctsk, Ctsl, Ctsz*), *Lamp1*, *Grn*, and vacuolar ATPase encoding genes (*Atp6v1b2, Atp6v1c1*), were all upregulated in mutant cells (Supplementary Figure 2). Noteworthy*, Lamp2*, although less expressed than the genes above, was also upregulated (Supplementary Figure 2). The expression of *Tfeb*, which encodes for a transcription factor controlling lysosomal biogenesis (Napolitano and Ballabio, 2016) was not modified suggesting that biogenesis of lysosomes was likely not modified by peroxisomal defect. In contrast, lysosomal metabolic, degradative, and signaling functions appear to be widely affected and mainly activated. In addition, motility and positioning of lysosomes should be affected regarding the virtual knock out of *Map2*, which encodes for a microtubule-associated protein 2 (Pu et al., 2016), and the induction of *Hook1* (Hook microtubule tethering protein 1) and *Plekhm2* (*SKIP*, kinesin-interacting protein). Cathepsins form an important group of proteases in the endosomal-lysosomal compartment and 14 cathepsin genes were found expressed in BV-2 cells. *Ctsk and Ctsb*, which code for two lysosomal proteins also known to be secreted and play an important role in the brain, particularly in microglia (Dauth et al., 2011; Nakanishi, 2020a), figured in the most expressed cathepsin genes (Supplementary Figure 2). With the exception of the poorly expressed *Ctsw* gene, cathepsin genes were found mostly upregulated in the mutant cells (Figures 4B,C). We also observed upregulation of all V-ATPase encoding genes expressed in BV-2 cells along with the *Atp6ap1* and *Atp6ap2* genes which encode accessory subunits involved in V-ATPase regulation (Jansen and Martens, 2012; Figures 4B,D). Western blotting analysis of several lysosomal proteins was performed to confirm the transcriptomic data. Upregulation of both cathepsins B and K and two other lysosomal proteins, progranulin (GRN) and galectin-3 (LGALS3, a cytosolic protein recruited to damaged lysosomes), was observed in the mutant cells (Figure 5A; Supplementary Figure 3). Upregulation of ATP6V0D2 was observed in the mutant cells except in the *Abcd2*^−/−^ genotype. However, we failed to show increased expression of ATP6V1B2 and LAMP2, except in *Abcd1*^−/−^ cells for the latter (Figure 5A; Supplementary Figure 3).

Autophagy is tightly coupled to lysosomal activity and plays an important role in microglia regarding neuroinflammation and brain homeostasis (Julg et al., 2021). Moreover, peroxisomal β-oxidation has recently been linked to the lysosomal localization and activation of mTORC1 complex (He et al., 2020). Besides, the heterodimeric Rag GTPases, the Lamtor/Ragulator complex that tethers the Rags to the lysosome, and the V-ATPase form a signaling system coupled to the rapamycin complex I (mTORC1) activity (Bar-Peled et al., 2012; Colacurcio and Nixon, 2016; Shen et al., 2016). *Rragc* and *Rragd* were u-regulated in mutant cells while *Rraga* expression remained unchanged. With the exception of the strong repression of *Lamtor4*, we observed a weak but significant induction of *Lamtor1, Lamtor3, and Lamtor*5. *Lamtor2* demonstrated a significant induction but below the 0.58 cut off. We observed a majority of autophagy-related (*Atg*) genes non differentially expressed but *Atg10* and *Atg13* were induced while *Atg9a* was repressed. Of note, *Atg7* expression was also found upregulated in *Abcd2*^−/−^ and *Acox1*^−/−^ cells. Moreover, *Sqstm1* (P62), *Rubcnl*, *Map1lc3a* (which exhibit two forms, LC3-I and LC3-II, the latter being induced in response to autophagic stimulus), and *Map1lc3b* genes were weakly upregulated in the mutant cells. To confirm the impact of peroxisomal mutations on autophagy, we carried out western blotting analysis of various autophagy markers and explored the phosphorylation of mTOR and ULK1, which represents the main mechanism of autophagy induction. mTORC1 directly downregulates autophagy by phosphorylating ULK1 on Ser757. LAMTOR4 repression and ATG13 induction were confirmed in each mutant genotype (Figure 5A; Supplementary Figure 3). We observed a weak induction of LC3-II, mTOR, ULK1 and p-ULK1 especially in the *Abcd1*^−/−^ and *Abcd1*^−/−^*Abcd2*^−/−^ cells while the expression of P62, LC3-I, and p-mTOR remained almost unchanged in the mutant cell lines (Figure 5A; Supplementary Figure 3). In addition to the autophagic and lysosomal markers, we analyzed the expression at the protein level of important genes associated with microglial functions related to lipids, i.e., *Apoe* and *CD36*. Western blotting results were in accordance with the transcriptomic data (Figure 5A; Supplementary Figure 3). The increased LC3-II/LC3-I ratio in the *Abcd1*^−/−^ and in the *Abcd1*^−/−^*Abcd2*^−/−^ cells suggest increased autophagy in these genotypes even though the p-mTOR/mTOR ratio and p-ULK1/ULK1 ratio remained almost unchanged (Figure 5A). Ultrastructure analysis by electron microscopy using diaminobenzidine staining to identify peroxisomes indeed revealed an increased number of autophagic figures in the mutant cell lines, especially in the *Abcd1*^−/−^*Abcd2*^−/−^ cells (Figure 5B).

#### DAM-like signature

**Figure 6:**
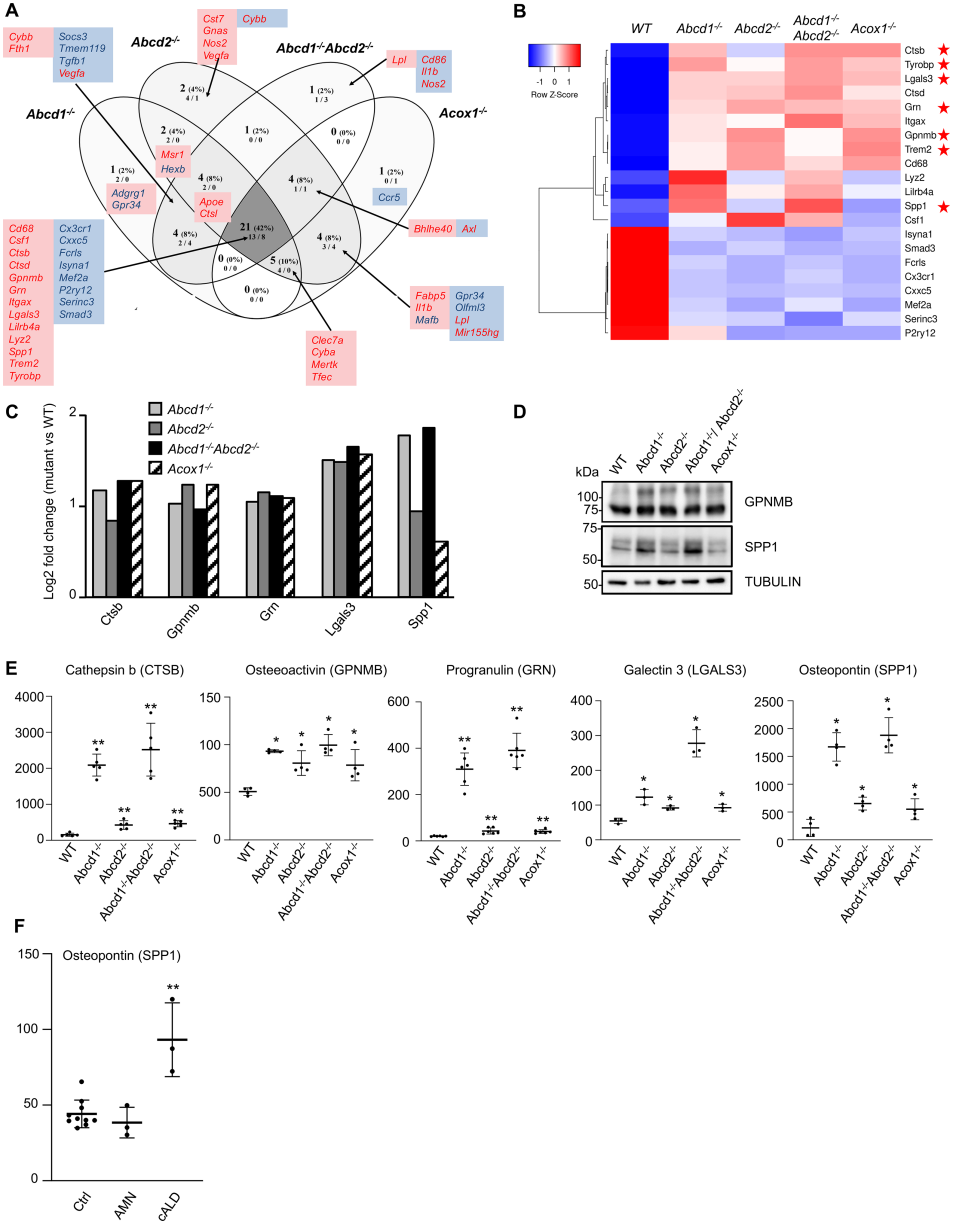
DAM-like signature in mutant cells. **(A)** Comparative analysis of the sets of DEGs using Venn diagram (*n* = 3 for each genotype). From the 59 genes associated with lipid metabolism and expressed in BV-2 cells, the significant genes (number and percentage) were selected using a cut off adjusted *p*-value lower than 0.05 and an absolute log2 FC higher than 0.58 (higher than 1.5-fold change in both directions) and are indicated in each group [*Abcd1^−/−^* (37 DEGs: 25 up, 12 down), *Abcd2^−/−^* (43 DEGs: 29 up, 14 down), *Abcd1^−/−^Abcd2^−/−^* (35 DEGs: 19 up, 16 down), and *Acox1^−/−^* (35 DEGs: 21 up, 14 down)]. Numbers below correspond to the genes found in each group when the analysis was performed only on upregulated genes (left) and downregulated genes (right). DAM genes in red and blue are expected to be upregulated and downregulated, respectively. Red boxes (left) and blue boxes (right) represent the upregulated and downregulated DEGs, respectively. **(B)** Expression-based heatmap showing hierarchical clustering (left) using average linking and Pearson distances based on the 21 DEGs of the intersection between the 4 mutant genotypes (right). Each point corresponds to the Row Z-score collected from the means of the reads normalized and divided by median of transcripts length in kb from 3 independent cell samples for each genotype. Red stars point the five DAM genes further studied and the *Trem2* and *Tyrobp* genes known as major hubs of the microglial activation. **(C)** Differential gene expression between mutant and WT BV-2 cells concerning the Cathepsin b (*Ctsb*), Osteoactivin (*Gpnmb*), Progranulin (*Grn*), Galectin-3 (*Lgals3*), and Osteopontin (*Spp1*) genes that belong the DAM signature. The histogram represents the log2 FC [with an adjusted *p*-value lower than 0.05 (DESeq2 Wald test with Benjamini and Hochberg *p*-value adjustment)] for each gene based on RNA-seq data. **(D)** Western blotting analysis of DAM proteins [Osteopontin (SPP1) and Osteoactivin (GPNMB)] from cell lysates of WT and mutant BV-2 cells. Western blotting showing Cathepsin b (CTSB), Progranulin (GRN), and Galectin-3 (LGALS3) expression has been shown in Figure 5A. For densitometric analysis, see Supplementary Figure 3. Source data are available online for this figure. **(E)** Increased secretion of DAM markers in BV-2 mutant cells. Dot plots representation of the concentration (ng/mL) of the DAM markers obtained by ELISA from the supernatant of mutant and WT BV-2 cells. Statistical significant differences are indicated: ^*^p < 0.05, ^**^p < 0.01 (Mann–Whitney test from 3 to 6 experiments). **(F)** Increased plasma level of osteopontin in cALD. Dot plots representation of the plasma level (ng/mL) of osteopontin (SPP1) obtained by ELISA from healthy controls (10), AMN patients (3), and cALD patients (3). Statistical significant differences are indicated: ^**^p < 0.01 (Mann–Whitney test).

A list of 59 genes expressed in BV-2 microglial cells was established from the review of Butovsky and Weiner and other transcriptomic analyses on microglia, which established a common signature associated with neurodegenerative pathologies (Keren-Shaul et al., 2017; Krasemann et al., 2017; Butovsky and Weiner, 2018; Deczkowska et al., 2018; Bonham et al., 2019). The DEGs belonging to this list include 27 repressed genes and 32 induced genes and define a DAM-like signature distinct from that of microglial activation or polarization. In the BV-2 mutant cells, the variations of expression of the majority of these genes were significant and their orientation was in agreement with those of the DAM signature. We found a total of 50 genes differentially expressed with 8 downregulated “homeostatic genes” (*Cx3cr1, Cxxc5, Fcrls, Isyna1, Mef2a, P2ry12, Serinc3, Smad3*) and 13 upregulated genes (*Cd68, Csf1, Ctsb, Ctsd, Gpnmb, Grn, Itgax, Lgals3, Lilrb4a, Lyz2, Spp1, Trem2, Tyrobp*) shared by the 4 mutant genotypes (Figures 6A,B; Supplementary Figure 4). If the intersection constitutes the largest group of DEGs, individual mutants have demonstrated specificity and, in some cases, contradictory results. For instance, *Abcd1*^−/−^*Abcd2*^−/−^ cells showed repression of *Cd86*, *Il1b, and Nos2* genes that should be upregulated, and induction of *Lpl*. Here again, we noticed a greater similarity of expression pattern between *Abcd1*^−/−^ and *Abcd1*^−/−^*Abcd2*^−/−^ and between *Abcd2*^−/−^ and *Acox1*^−/−^ (Figure 6A). Altogether, transcriptomic results indicate that peroxisomal defects trigger a transcriptomic signature very similar to the DAM signature encountered in common neurodegenerative disorders.

We focused on 5 upregulated genes of the DAM list which encode secreted proteins: Cathepsin b (*Ctsb*), Osteactivin (*Gpnmb*), Progranulin (*Grn*), Galectin-3 (*Lgals3*), Osteopontin (*Spp1*; Figure 6C). Cathepsin B has an important function in microglia and inflammatory control with its participation to the secretion of IL1β (Nakanishi, 2020b). Elevated levels of cathepsin B have been detected in biological fluids of patients with brain diseases (Hook et al., 2020). Osteactivin, encoded by *Gpnmb*, is a transmembrane glycoprotein that would function as a negative regulator of inflammatory processes and that can be secreted as a soluble fragment upon lysosomal stress (van der Lienden et al., 2019). Osteactivin is thought to carry anti-inflammatory and reparative functions and has been demonstrated to be neuroprotective (Budge et al., 2018). Progranulin, encoded by *Grn*, is a secreted protein that not only contributes to neuroinflammation but also actively participates in the regulation of lysosomal functions and autophagy (Chitramuthu et al., 2017; Paushter et al., 2018; Elia et al., 2019; Mendsaikhan et al., 2019). Regarding the lysosomal trafficking of progranulin, it depends on prosaposin and sortilin; it impacts the production of granulin peptides and interferes with the secretory pathway (Du et al., 2022). Interestingly, *Sort1* was found highly repressed (log2 FC = −5) while *Psap* was upregulated (log2 FC = 1) in the 4 mutant cell lines. Galectin-3, which is also secreted by microglia, has demonstrated pro-differentiating effects on oligodendrocyte progenitor cells and pro-inflammatory properties acting as a TLR4 ligand in an autocrine regulation (Burguillos et al., 2015; Thomas and Pasquini, 2018). It is worth noting that both *Lgals3* and *Tlr4* genes were upregulated in the four mutant genotypes. Galectin-3 was also recently described as an endogenous ligand of TREM2 and would therefore participate to the TREM2/DAP12 signaling cascade in an autocrine manner (Boza-Serrano et al., 2019). Osteopontin, encoded by *Spp1*, is a pro-inflammatory secreted protein which has been shown to inhibit autophagosome-lysosome fusion (Tang et al., 2020) and is thought to play a role in pathogenesis of neurodegenerative diseases or in neuroprotection by regulating the activation and function of microglia (Yu et al., 2017). We confirmed an increased expression of *Ctsb, Gpnmb, Grn, Lgals3, and Spp1* by western blotting (Figures 5A, 6D; Supplementary Figure 4). We performed ELISA experiments on the culture medium of each mutant cell line to assess the secretion levels of these 5 proteins. Significant increase of secretion of each protein was found in the mutant cells as compared with the WT cells (Figure 6E). The highest relative increase compared with the WT cells was found in the *Abcd1*^−/−^*Abcd2*^−/−^ cells (CTSB: 2518 ng/mL, 16.2-fold; GPNMB: 99.5 ng/mL, 1.96-fold; GRN: 391 ng/mL, 19-fold; LGALS3: 278 ng/mL, 5.08-fold; SPP1: 1,881 ng/mL, 8.75-fold).

Osteopontin is an inflammatory protein that has recently been described as a biomarker of Alzheimer’s disease (Chai et al., 2021) and multiple sclerosis (Agah et al., 2018). In X-ALD, microglial defect is thought to precede the neurological involvements (Eichler et al., 2008; Bergner et al., 2019). Since osteopontin appears to be oversecreted from microglial cells in case of peroxisomal defect, osteopontin could therefore represent an interesting biomarker for X-ALD. To address this point, we conducted a pilot study on the plasma level of osteopontin using samples from 3 adult patients diagnosed with a cerebral form of X-ALD (cALD) and 3 adult patients with adrenomyeloneuropathy. The results were compared with control values taken from 10 healthy controls. While there was no significant difference in AMN patients, a 2.11-fold increased concentration was observed in cALD patients (Figure 6F).

## Discussion

The transcriptomic analysis of the mutant cell lines revealed 1,000 of DEGs. As expected, genes related to lipid metabolism belong to the DEGs but “immune system process” ranked first in the significant enriched GO terms, which support a role of peroxisomal metabolism far beyond its main known metabolic functions. Several recent studies have documented a functional role of peroxisomes on immune functions and cell signaling (Di Cara et al., 2019; Di Cara, 2020; Di Cara et al., 2023) and the present study reinforces this statement. Although many signaling pathways, cell division and cell cycle features, phagocytosis, and immune response of microglial BV-2 mutant cells appear to be largely affected, we focused on lipid metabolism, lysosome, autophagy, and microglial plasticity in link with neurodegenerative processes.

Although all 4 mutant genotypes result in a defect in peroxisomal β-oxidation, different phenotypes were expected because of the predicted severity of the mutations, in descending order: *Acox1*^−/−^, *Abcd1*^−/−^*Abcd2*^−/−^, *Abcd1*^−/−^, and finally *Abcd2*^−/−^. Indeed, there is currently no disease associated with a mutation in the *ABCD2* gene. Moreover, since both *Abcd1* and *Abcd2* genes are expressed in BV-2 cells and these genes have partial functional redundancy, a single KO should have resulted in an attenuated phenotype compared to the double KO. Finally, since ACOX1 is the rate-limiting enzyme in peroxisomal β-oxidation, the most severe phenotype was expected for this KO. Autophagic alterations and increased secretion of DAM proteins was indeed more important in the *Abcd1*^−/−^*Abcd2*^−/−^ genotype However, it was quite puzzling to observe in some cases that the dysregulations are less marked in the *Abcd1*^−/−^*Abcd2*^−/−^ cells than in the single mutants. These indirect effects of peroxisomal defects are probably coming along with compensatory mechanisms, which differ depending on the genotypes. More pronounced feedback loop may exist in the case of the double knock out. VLCFA accumulation, as a marker of peroxisomal defect, was seen only in the *Acox1*^−/−^ and *Abcd1*^−/−^*Abcd2*^−/−^ cells and cannot be the sole cause of the phenotype differences (Raas et al., 2019a,b). Surprisingly, several clusters of DEGs were found associated either with *Abcd1*^−/−^ and *Abcd1*^−/−^*Abcd2*^−/−^ or with *Acox1*^−/−^ and *Abcd2*^−/−^ cells and sometimes in a counterintuitive manner. Lipidomic differences have already been described between ACOX1 and ABCD1 human fibroblasts which reflects the heterogeneity of the peroxisomal disorders ACOX1 deficiency and X-ALD (Herzog et al., 2018). Accumulation of phospholipids with VLCFAs was observed in the majority of phospholipid classes in ACOX1-deficient cells but was limited to phosphatidylcholine and phosphatidylethanolamine classes in ABCD1-deficient fibroblasts. The observed proximity between *Acox1*^−/−^ and *Abcd2*^−/−^ BV-2 genotypes observed with several genes question about a functional link between these two genes. Is it associated with a specific substrate of ABCD2, which is metabolized by ACOX1? One possible explanation could be linked to the accumulation of a specific lipid (probably a PUFA not transported by ABCD1) leading to the sustained activation of transcription factors such as PPARs. PUFAs and some of their derivatives have been demonstrated to bind PPARs and regulate microglial functions (Ajmone-Cat et al., 2012; Leyrolle et al., 2019). Interestingly, *Pparβ/δ* appears to be the most expressed isotype in the BV-2 cells and its expression was found repressed in both *Abcd1^−/−^* and *Abcd1^−/−^Abcd2^−/−^* cells, but not in the other mutants, whereas *Pparγ* was found upregulated in both *Acox1*^−/−^ and *Abcd2*^−/−^ cells.

In spite of this observation, the most numerous group of DEGs was found in the intersection group concerning the four mutant genotypes. It was both true for the global analysis and for the specific analyses related to lipid metabolism and other pathways. If LCFA and VLCFA accumulation is considered as the hallmark of peroxisomal diseases, from our results, we can conclude that peroxisomal defects have induced an in-depth reprogramming of genes involved in lipid metabolism. Fatty acid oxidation and elongation, fatty acid release and signaling, PUFA metabolism and production of bioactive PUFA derivatives, metabolism of membrane lipids, cholesterol metabolism and trafficking, have all been found impacted in the mutant cells. From these data, we could not conclude whether the mutations were leading to a clear upregulation or downregulation of these lipid metabolic pathways since most of the pathways were represented in both upregulated and downregulated DEGs. An extended lipidomic study is needed to explore in detail the impact of the mutations on the various classes of lipids and complete the preliminary analysis on total FA and cholesterol precursors of the mutant cell lines (Raas et al., 2019a,b). Further studies are also needed to understand the origin of cholesterol accumulation, especially in the context of DHCR24 loss, and document to which extent the observed modifications in FA content, in membrane cholesterol and in cholesterol precursors, alter the biophysical properties of membranes, as well as their signaling and inflammatory associated functions.

Nevertheless, such metabolic reprogramming is known to impact microglial functions related to inflammatory signaling, oxidative stress, phagocytosis, and brain homeostasis (Nadjar, 2018; Fitzner et al., 2020; Loving and Bruce, 2020; Chausse et al., 2021; Navia-Pelaez et al., 2021). Peroxisomal β-oxidation defect in microglia using the *Cx3cr1*-*Mfp2*^−/−^ mice (targeted gene *Hsd17b4*) led to an inflammatory state (Beckers et al., 2019). In *Drosophila* plasmatocytes, fly equivalent of mammalian macrophages, defective peroxisomal β-oxidation was also shown to modify glycerophospholipids with consequences on cytoskeleton and signaling related to inflammation (Nath et al., 2022). Therefore, from our observations and the microglial literature, the defect in peroxisomal ABC transporters and ACOX1 in BV-2 cells likely contribute to a shift toward a pro-inflammatory state and a modification of phagocytosis ability independently of the supposed severity of the metabolic defects associated with the different mutant genotypes. In agreement with this hypothesis, in all the mutant microglial cells, we found upregulation of the *Trem2, Tyrobp* and *Apoe* genes, which constitute essential hub genes for microglial functions (Loving and Bruce, 2020). Further experiments of phagocytosis using myelin debris or apoptotic brain cells for instance, and an in-depth analysis of the inflammatory response of the mutant cells are needed to confirm this hypothesis.

A striking observation of the analysis of DEGs in our study was the virtual knock out of genes associated with lipid metabolism such as *Scd1, Fads2, Acer2,* or *Dhcr24*. Both *Scd1* and *Dhcr24* genes impact the polarization of microglial cells and are clearly associated with microglial functions such as phagocytosis and inflammatory control (Bogie et al., 2020; Zu et al., 2020). Reduced expression of *DHCR24* was observed in Alzheimer’s disease (Iivonen et al., 2002; Zerenturk et al., 2013). The functional loss of *Scd1* was shown to trigger complex metabolic changes (Flowers and Ntambi, 2008) and has also been associated to inflammation and cellular stress (Liu et al., 2011). Interestingly, decreased SCD1 activity has been shown to increase the accumulation of saturated VLCFA (Raas et al., 2021). Regarding the Δ6-desaturase gene (*Fads2*), its disruption not only impacts PUFA content but also triggers triglyceride and cholesterol accumulation (Hayashi et al., 2021). Membrane lipid modifications and the activation of various signaling pathways related to LXR or PPAR for example, are also likely (Bogie et al., 2013; Zerenturk et al., 2013) but remain to be clarified in our mutant cell lines. Regarding the shutdown of *Acer2*, which is involved in the hydrolysis of long chain and very-long chain ceramides and dihydroceramides to generate sphingosine, important signaling consequences affecting microglia are expected (Li et al., 2018). Of note, impairment of alkaline ceramidase 3, which is specific for unsaturated long chain ceramides causes early-onset progressive leukoencephalopathy (Pant et al., 2020).

Alterations of lipid metabolism, lysosomal function, phagocytosis and autophagy, appear to form a cluster of microglial abnormalities found in common neurodegenerative diseases. Autophagy is connected to lipid metabolism and its alteration has been shown to impact the role of microglia in neuroinflammatory control (Julg et al., 2021; Xu et al., 2021). Here, we presented several evidences of increased autophagy in the mutant cell lines, mainly in the *Abcd1^−/−^Abcd2^−/−^* cells, with an increased expression of key autophagic genes, an increased LC3-II/LC3-I ratio together with the observation of autophagic figures by electron microscopy. The full repression of *Lamtor4* also in agreement with an induction of the autophagic process (Schweitzer et al., 2015). Since the phosphorylation of mTOR and ULK1 was not clearly altered, our results suggest an mTOR-independent induction of autophagy. This mechanism remains to be characterized but could be related to the increase lysosomal activity (Zhang X. D. et al., 2013) and/or to the inositol signaling pathway since many related phospholipase genes were strongly deregulated (Sarkar, 2013). The autophagy induction observed mainly in the *Abcd1^−/−^Abcd2^−/−^* genotype seems to be in contradiction with the impaired autophagic flux observed in X-ALD fibroblasts and presented as a neuroprotective mechanism (Launay et al., 2015) but fits with the increased autophagy observed in case of *Acox1* defect (He et al., 2020). Concerning lysosomes, beyond the fact that lysosomal DEGs were sorted first in GO analysis, that cathepsin and V-ATPase genes were almost all upregulated, we confirmed at the protein level the upregulation of several lysosomal markers (ATP6V0D2, CTSB, CTSK, GRN, LGALS3) and their increased secretions (CTSB, GRN, LGALS3). Of note, the activation of various lysosomal enzymatic activities by VLCFA observed in skin fibroblasts, was suggested to participate in the pathogenesis of X-ALD (Dhaunsi et al., 2005). Moreover, the increased expression and secretion of cathepsins, especially CTSB, could represent a key component of the pathogenesis of X-ALD as a major driver of neuroinflammation (Ni et al., 2015; Lowry and Klegeris, 2018; Nakanishi, 2020b). Alteration of the lysosomal acidification has been associated with aging and adult-onset neurodegenerative diseases (Colacurcio and Nixon, 2016). In contrast, overexpression of V-ATPases has been observed in tumors and likely participates in the activation of lysosomal hydrolases, autophagic flux, and various signaling pathways (Pamarthy et al., 2018). Altogether, our transcriptomic study has clearly demonstrated that peroxisomal defects induce autophagic and lysosomal alterations like in common neurodegenerative diseases. Does it mean that peroxisomal functions are affected in most of the neurodegenerative diseases or does it mean that different triggering defects converge toward a common microglial pathologic state? It is too early to conclude even though several peroxisomal markers have been described in Alzheimer’s disease or multiple sclerosis (Zarrouk et al., 2020). It is however clear that lysosomes and peroxisomes are tightly connected and that peroxisomal defects impact lysosomal lipid transport and intracellular clearance, and therefore microglial functions (Chu et al., 2015; Schrader et al., 2020).

Beyond the metabolic reprograming, we identified a transcriptomic signature resembling that of the DAM signature identified in common neurodegenerative diseases (Keren-Shaul et al., 2017; Butovsky and Weiner, 2018). Despite a few exceptions, most of the “homeostatic genes” of the DAM signature were found repressed in the mutant cells while the “neurodegenerative genes” were found upregulated. Therefore, our findings identify peroxisomal defect as a new triggering event to lose the signature of homeostatic microglia and shift toward the DAM phenotype along with aging and the most common neurodegenerative diseases. A puzzling question concerns *Abcd1* null mice which do not develop brain demyelination contrary to human. To our knowledge, primary microglia was not investigated in these mice with respect to the DAM signature but was shown to overexpress *Trem2* (Gong et al., 2017). Such discrepancy between human and mice could be due to *Abcd2* since human microglia do not express *Abcd2* (Olah et al., 2020) but *Abcd1/Abcd2* double knockout mice do not show demyelination in spite of an earlier and more severe phenotype. Species differences may involve not only microglia but also other cell types and possibly the blood–brain barrier. In any cases, correcting the peroxisomal defect in microglia and restoring homeostatic state represents a promising strategy in peroxisomal leukodystrophies.

In the context of X-ALD diagnosis and follow up, with the expansion of neonatal screening and improved diagnosis, a complementary tool in addition to brain MRI follow-up is needed. Since VLCFA levels are not correlated with neurodegenerative involvements and since brain MRI is not easy to manage, there is an urgent need for prognostic biomarkers to evaluate disease progression and improve the follow-up of patients (Honey et al., 2021). Recent studies identified and evaluated neurofilament light chain as a potential biomarker for cALD (Weinhofer et al., 2021; Wang et al., 2022). In this study, we identified 5 proteins of the DAM signature which are over secreted by the mutant cells that could virtually represent relevant biomarkers of disease progression. Cathepsin B, progranulin, galectin-3, osteactivin and osteopontin are secreted proteins that have been found in biological fluid of patients suffering from neurodegenerative disorders (Kramer et al., 2016; Agah et al., 2018; Moloney et al., 2018; Batzu et al., 2019; Siew et al., 2019; Chai et al., 2021). Here, we confirmed in this pilot study that the plasma level of osteopontin is significantly higher in patients with cALD than in AMN or healthy controls. In spite of this interesting result, a clinical study conducted on a larger cohort and including longitudinal follow-up of young boys is necessary to investigate the use of osteopontin as a biomarker of the cerebral form of X-ALD and to explore whether the 4 other proteins may also be useful. During the preparation of the manuscript, the level of osteactivin (GPNMB) in cerebrospinal fluid was found correlated with MRI disease severity score in cALD patients (Taghizadeh et al., 2022), which reinforces the relevance of our results.

In conclusion, our study demonstrates that BV-2 microglial cells with a peroxisomal defect lose their homeostatic signature and adopt a DAM-like signature accompanied with a large reprogramming in lipid metabolism, signaling, lysosomal and autophagic genes. It would be interesting to confirm such observations in primary microglial cells from peroxisomal knock out mice or human induced pluripotent stem cells into microglia-like cells for instance (Pandya et al., 2017) but functional consequences including secretion of inflammatory cytokines and immune response, phagocytosis, are likely. While these BV-2 murine microglial cell models have limitations relative to primary microglial cells and their relevance to humans, they have offered the opportunity to explore in detail and robustly the consequences of a peroxisomal defect in a key cell type of the peroxisomal leukodystrophies and to discover potential biomarkers for which further clinical studies may confirm value.

## Data availability statement

The datasets presented in this study can be found in online repositories. The names of the repository/repositories and accession number(s) can be found at: https://www.ncbi.nlm.nih.gov/, GSE200022.

## Ethics statement

Ethical review and approval was not required for the study on human participants in accordance with the local legislation and institutional requirements. Written informed consent for participation was not required for this study in accordance with the national legislation and the institutional requirements.

## Author contributions

QR, AT, MT-J, and CG were in charge of cell culture, mRNA, and protein purification. QR, AT, MT-J, and CG performed experiments related to protein expression. VL performed lipid analysis. RK and CK performed RNA-seq and the related bioinformatics and statistical analyses. QR, AT, PA, and SS performed heatmap, gene ontology analyses, V-Plot, and MA-Plot analyses. EB, MB, and YH, performed Filipin labeling and cytometry analyses. QR, AT, MT-J, CG, AB, PA, YH, CT, and MC-M analyzed the data. CT, CG, and SS performed statistical analyses. QR, AT, MT-J, CG, PA, and SS mounted the figures. SS conceived the project, performed analyses, and wrote the manuscript with QR, AT, MT-J, CG, YH, FC, MC-M, PA, DT, AB, and BN. All authors contributed to the article and approved the submitted version.

## Funding

We warmly acknowledge the Fondation Maladies Rares which supported our transcriptomic analysis project (GenOmics: High throughput sequencing and rare diseases, call 2017-20170615). Sequencing was performed by the GenomEast platform, a member of the “France Génomique” consortium (ANR-10-INBS-0009). We would like to acknowledge the SATT Sayens of Dijon, the CHU of Dijon (project CRBSEP-EMATSEP grant), the Canadian New Frontiers Research Funds Exploration (NFRF-E 19-00007), and the regional council of Bourgogne Franche-Comté (Project PERSIL 2019) for their support. We are also grateful to networking support by the COST Action CA 16,112 NutRedOx (Personalized Nutrition in aging society: redox control of major age-related diseases), supported by COST (European Cooperation in Science and Technology). We thank the Centre d’Immunologie de Marseille-Luminy (CIML) flow cytometry facility. This work was supported by institutional grants from INSERM, CNRS and Aix-Marseille University to the CIML and program grant from the French National Research Agency (ANR-17-CE15-0032). The project leading to this publication has received funding from Excellence Initiative of Aix-Marseille University—A*MIDEX, a French “Investissements d’Avenir” program. The laboratory BioPeroxIL was funded by the Ministère de l’Education Nationale et de l’Enseignement Supérieur et de la Recherche (France) and by the University of Bourgogne. MT-J was funded by CNRST (PhD excellence grant number: 17UHP2019, Morocco) and by the Action Intégrée of the Comité Mixte Inter-universitaire Franco-Marocain (n° TBK 19/92 n° Campus France: 41501RJ) from the PHC Toubkal program, Ministère des Affaires Étrangères.

## Conflict of interest

The authors declare that the research was conducted in the absence of any commercial or financial relationships that could be construed as a potential conflict of interest.

## Publisher’s note

All claims expressed in this article are solely those of the authors and do not necessarily represent those of their affiliated organizations, or those of the publisher, the editors and the reviewers. Any product that may be evaluated in this article, or claim that may be made by its manufacturer, is not guaranteed or endorsed by the publisher.

## Footnotes

^1^ https://www.ncbi.nlm.nih.gov/geo/query/acc.cgi?acc=GSE200022

^2^ https://bioinfogp.cnb.csic.es/tools/venny/index.html

^3^ https://huygens.science.uva.nl/VolcaNoseR/

^4^ https://david.ncifcrf.gov/tools.jsp

^5^ https://maayanlab.cloud/Enrichr/

^6^ http://www.heatmapper.ca/

## Abbreviations

AA: Arachidonic acid
ABC: ATP-binding cassette
ACOX1: Acyl-CoA oxidase 1
AMN: Adrenomyeloneuropathy
cALD: Cerebral adrenoleukodystrophy
CRISPR: Clustered regularly interspaced short palindromic repeat
DAM signature: Disease-associated microglial signature
DEGs: Differentially expressed genes
DHA: Docosahexaenoic acid
DPA: Docosapentaenoic acid
EPA: Eicosapentaenoic acid
FA: Fatty acids
FBS: Fetal bovine serum
log2 FC: log2 fold change
GC-MS: Gas chromatography mass spectrometry
L/VLCFA: Long/very long-chain fatty acids
MUFA: Monounsaturated fatty acids
PUFA: Polyunsaturated fatty acids
RNA-seq: RNA sequencing
WT: Wild type
X-ALD: X-linked adrenoleukodystrophy
