# Supplementary material for: Peroxisomal defects in microglial cells induce a disease-associated microglial signature
Source: Front Mol Neurosci. 2023 Apr 17;16:1170313. doi: 10.3389/fnmol.2023.1170313 (PMC10149961; doi:10.3389/fnmol.2023.1170313)

# Western-blot Source Data

## Table of Contents


- DHCR24 slides no. 2
- CTSB slide no. 3
- CTSK slide no. 4
- LAMP2 slides no. 5
- ATP6V0D2 slides no. 6-7-8-9
- ATP6V1B2 slides no. 10-11-12-13
- GRN slides no. 14-15-16-17
- LGALS3 slides no. 18-19-20-21
- ATG13 slides no. 22-23-24-25
- LC-3 slides no. 26-27-28-29-30
- P62 slides no. 31-32-33-34
- LAMTOR4 slides no. 35
- mTOR / p-mTOR slides no. 36-37-38-39
- ULK1 / p-ULK1 slides no. 40-41-42-43
- APOE slides no. 44-45-46-47
- CD36 slides no. 48-49-50-51
- GPNMB slides no. 52-53-54-55
- SPP1 slides no. 56-57-58-59

# DHCR24

**DHCR 24**
MW : 60 kDa
Observed : 55 kDa

Primary antibody : Cell Signaling # 2033

4-20% Gradient Gels
30 µg/load
PVDF Transfer

| | Transcriptomic |
|---|---|
| | Ratio Mean |
| WT | 1.0000 |
| KO D1 | 0.0005 |
| KO D2 | 0.0005 |
| KO D1/D2 | 0.0001 |
| KO Acox | 0.0001 |

## Samples serie S1 (21/10/21)
## WB Catherine 28/11/2022 – Mb « D1/2 »

BV2 WT | KO D1 | KO D2 | KO D1/D2 | KO Acox

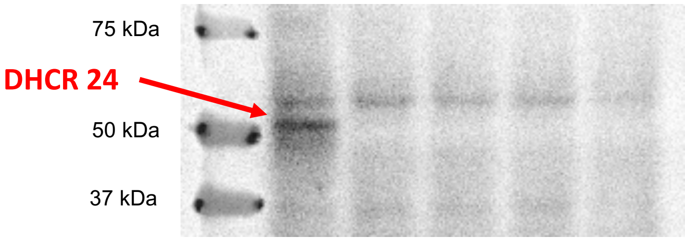


Expo 1200 sec
Membrane « D1/2 »

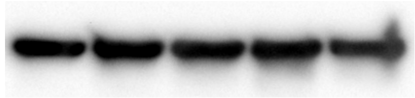


Expo 59.0 sec
Membrane « D1/2 »

## Samples serie S2 (27/10/21)
## WB Catherine 28/11/2022 – Mb « D1/2 »

BV2 WT | KO D1 | KO D2 | KO D1/D2 | KO Acox

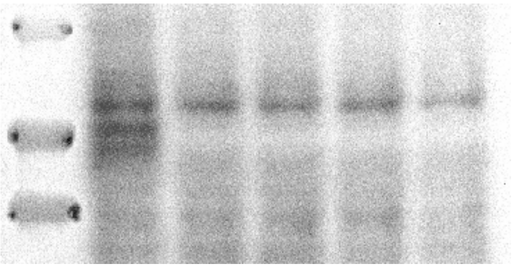


Expo 1200 sec
Membrane « D1/2 »

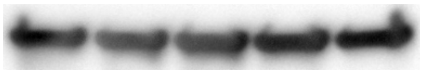


Expo 59.0 sec
Membrane « D1/2 »

## Samples serie S3 (28/10/21)
## WB Catherine 22/11/2022 – Mb «D2/3 »

BV2 WT | KO D1 | KO D2 | KO D1/D2 | KO Acox

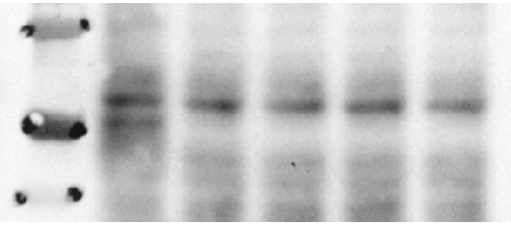


Expo 1200 sec
Membrane « D2/3 »

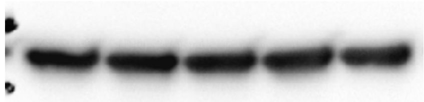


Expo 49.8 sec
Membrane « D2/3 »

*Mb selected for the publication*

# Cathepsin B

*Mb selected for the publication*

29-09-2021
MPS1 samples
Catherine and Ali

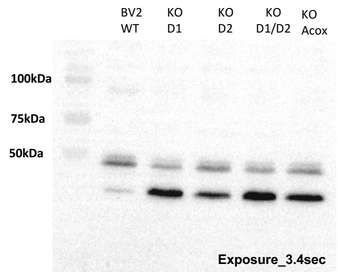


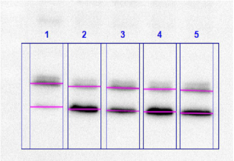


Tubuline

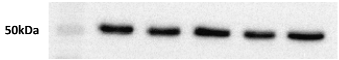


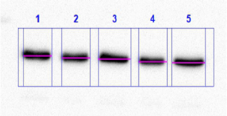


## Densito analysis

| 29-09-2021 MPS1 | n° bande | CathB | Tubuline | Ratio CathB/tub | Ratio /WT |
|---|---|---|---|---|---|
| WT | 1 | 5592356 | 28062125 | 0.199 | 1.00 |
| KO D1 | 2 | 12855073 | 23550540 | 0.546 | 2.74 |
| KO D2 | 3 | 9688371 | 31239100 | 0.310 | 1.56 |
| KO D1/D2 | 4 | 13882638 | 21395335 | 0.649 | 3.26 |
| KO ACOX1 | 5 | 11788161 | 29096752 | 0.405 | 2.03 |

F:\Mounia CathB mbne 10-29-09-2021-Echb Catherine MPS1\XRS 2021-09-29 14hr 05min_Exposure_3.4sec.scn

F:\Mounia CathB mbne 10-29-09-2021-Echb Catherine MPS1\Tubuline\XRS 2021-09-29 18hr 46min_Exposure_59.3sec.scn

23/24-09-2021
MPS3 samples
Catherine and Ali

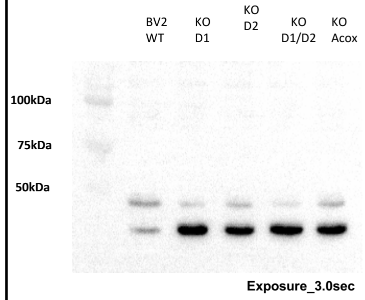


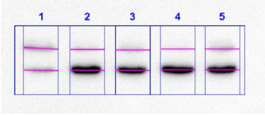


Tubuline

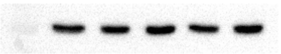


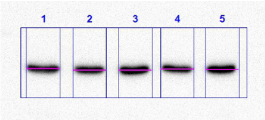


## Densito analysis

| 23/24-09-2021 MPS | n° bande | CathB | Tubuline | Ratio CathB/tub | Ratio /WT |
|---|---|---|---|---|---|
| WT | 1 | 3302065 | 17381376 | 0.190 | 1.00 |
| KO D1 | 2 | 11363820 | 16975552 | 0.669 | 3.52 |
| KO D2 | 3 | 8336575 | 21293376 | 0.392 | 2.06 |
| KO D1/D2 | 4 | 10784800 | 16284160 | 0.662 | 3.49 |
| KO ACOX1 | 5 | 10050365 | 21467200 | 0.468 | 2.46 |

F:\Mounia CathB mbne 9-23-09-2021-Ech Catherine MPS3\XRS 2021-09-23 14hr 11min Exposure_3.0sec

F:\Mounia CathB mbne 9-23-09-2021-Ech Catherine MPS3\Tubuline\XRS 2021-09-24 13hr 20min Exposure_59.3sec

29-09-2021
MPS2 samples
Catherine and Ali

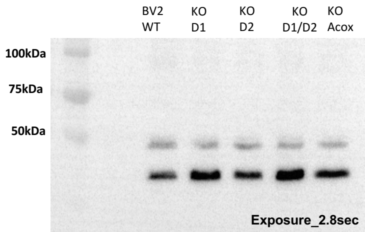


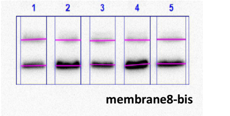


Tubuline

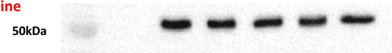


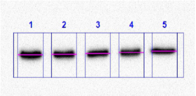


## Densito analysis

| 29-09-2021 MPS2 | n° bande | CathB | Tubuline | Ratio CathB/tub | Ratio /WT |
|---|---|---|---|---|---|
| WT | 1 | 6140610 | 18308096 | 0.335 | 1.00 |
| KO D1 | 2 | 10052343 | 16014400 | 0.628 | 1.87 |
| KO D2 | 3 | 6782139 | 15661568 | 0.433 | 1.29 |
| KO D1/D2 | 4 | 12596157 | 13354880 | 0.943 | 2.81 |
| KO ACOX1 | 5 | 8604936 | 14438208 | 0.596 | 1.78 |

F:\Mounia CathB mbne 8-bis-29-09-2021-ech Catherine MPS2\XRS 2021-09-29 13hr 54min

Exposure_59.3sec

F:\Mounia CathB mbne 8-bis-29-09-2021-ech Catherine MPS2\Tubuline\XRS 2021-09-29 18hr 36min_Exposure_59.3sec.scn

| | Transcriptomique |
|---|---|
| | Moyenne ratio |
| WT | 1 |
| KO D1 | 1,21 |
| KO D2 | 0,86 |
| KO D1/D2 | 1,33 |
| KO ACOX1 | 1,33 |

| | Moyenne ratio | Ecart-type |
|---|---|---|
| WT | 1.00 | 0.00 |
| KO D1 | 2.71 | 0.83 |
| KO D2 | 1.64 | 0.39 |
| KO D1/D2 | 3.18 | 0.34 |
| KO ACOX1 | 2.09 | 0.35 |

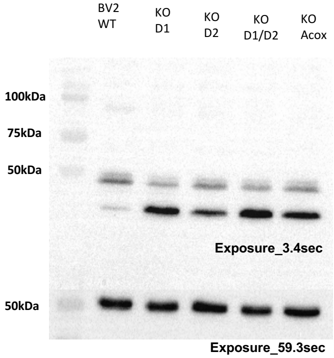


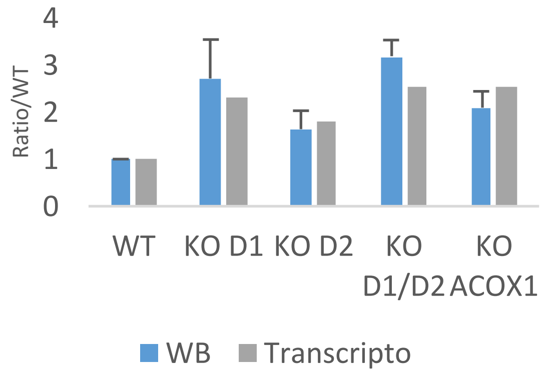

# Cathepsin K

20/26-01-2021
Samples
Mounia

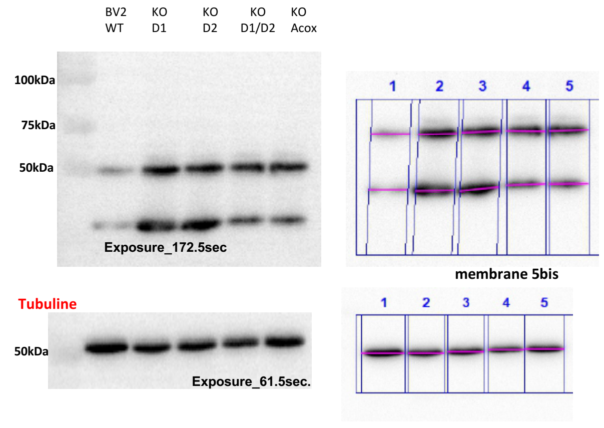


F:\Western blot Mounia CathK bis-test5-20 01 2021\XRS 2021-01-20 12hr 29min_Exposure_172.5sec.scn

F:\Western blot Mounia CathK bis-test5-20 01 2021\Tubuline sur mbn cathK -test5-bis-26 01 2021\XRS 2021-01-26 15hr 49min_Exposure_61.5sec.scn

## Densito analysis

| 20/26-01-2021 samples | n° bande | CathK | Tubuline | Ratio CathK/tub | Ratio /WT |
|---|---|---|---|---|---|
| WT | 1 | 16801848 | 29464364 | 0.570 | 1.00 |
| KO D1 | 2 | 56945259 | 21739230 | 2.619 | 4.59 |
| KO D2 | 3 | 53175248 | 21864976 | 2.432 | 4.26 |
| KO D1/D2 | 4 | 36881600 | 18307080 | 2.015 | 3.53 |
| KO ACOX1 | 5 | 38047856 | 23367680 | 1.628 | 2.86 |

*Mb selected for the publication*

23/24-09-2021
MPS3 samples
Catherine and Ali

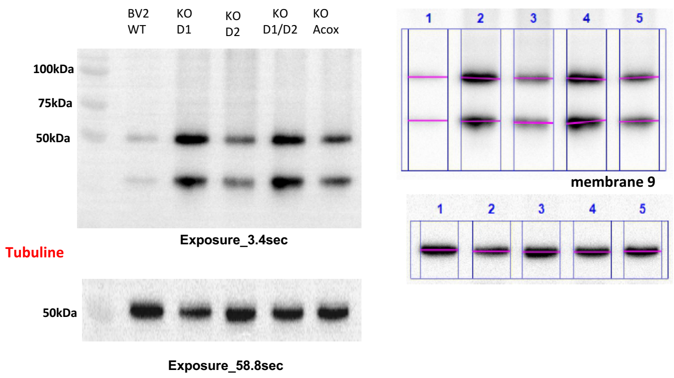


F:\Mounia CathK mbne 9-24-09-2021-Ech Catherine MPS3\XRS 2021-09-24 12hr 58min_Exposure_3.4sec.scn

F:\Mounia CathK mbne 9-24-09-2021-Ech Catherine MPS3\Tubuline\XRS 2021-09-24 18hr 05min_Exposure_58.8sec.scn

## Densito analysis

| 23/24-09-2021 MPS3 samples | n° bande | CathK | Tubuline | Ratio CathK/tub | Ratio /WT |
|---|---|---|---|---|---|
| WT | 1 | 4551172 | 15939642 | 0.286 | 1.00 |
| KO D1 | 2 | 44655996 | 10964762 | 4.073 | 14.26 |
| KO D2 | 3 | 21615370 | 15485182 | 1.396 | 4.89 |
| KO D1/D2 | 4 | 49135496 | 12854770 | 3.822 | 13.39 |
| KO ACOX1 | 5 | 26148872 | 13637334 | 1.917 | 6.72 |

24-09-2021
MPS2 samples
Catherine and Ali

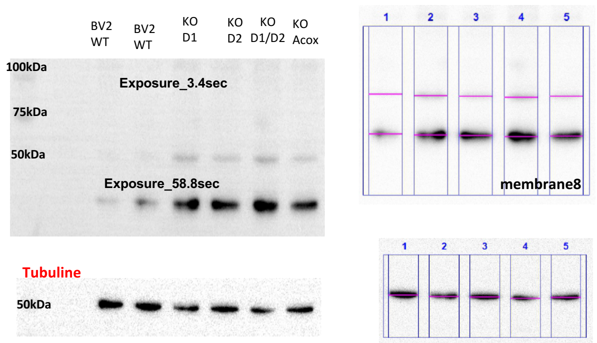


F:\Mounia CathK mbne 8-24-09-2021-Ech Catherine MPS2\XRS 2021-09-24 12hr 48min_Exposure_3.4sec.scn

F:\Mounia CathK mbne 8-24-09-2021-Ech Catherine MPS2\Tubuline\XRS 2021-09-24 17hr 55min_Exposure_58.8sec.scn

## Densito analysis

| 24-09-2021 MPS2 samples | n° bande | CathK | Tubuline | Ratio CathK/tub | Ratio /WT |
|---|---|---|---|---|---|
| WT | 1 | 4857204 | 11053360 | 0.439 | 1.00 |
| KO D1 | 2 | 17787056 | 6225854 | 2.857 | 6.50 |
| KO D2 | 3 | 17298124 | 9066260 | 1.908 | 4.34 |
| KO D1/D2 | 4 | 23384664 | 5171296 | 4.522 | 10.29 |
| KO ACOX1 | 5 | 13746454 | 7946416 | 1.730 | 3.94 |

| | Transcriptomique |
|---|---|
| | Moyenne ratio |
| WT | 1 |
| KO D1 | 1,05 |
| KO D2 | 0,98 |
| KO D1/D2 | 1,41 |
| KO ACOX1 | 0,86 |

| | Moyenne ratio | Ecart-type |
|---|---|---|
| WT | 1.000 | 0.000 |
| KO D1 | 8.453 | 5.122 |
| KO D2 | 4.499 | 0.340 |
| KO D1/D2 | 9.070 | 5.039 |
| KO ACOX1 | 4.502 | 1.991 |

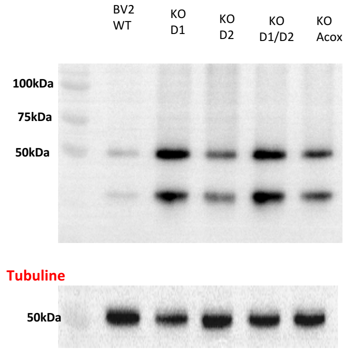


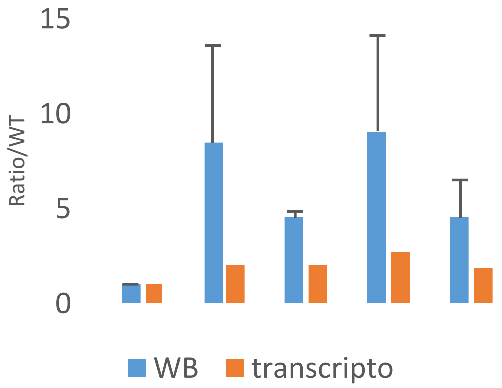

# Lamp2

12-10-2021
Samples
Mounia

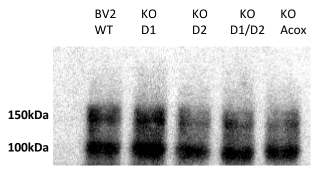


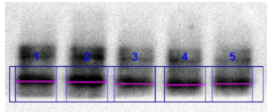


membrane 8-bis

_Exposure_687.0sec

Tubuline

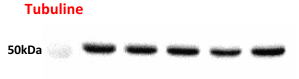


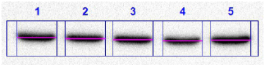


Exposure_59.9sec

## Densito analysis

| 12-10-2021 Samples Mounia | n° bande | Lamp2 | Tubuline | Ratio Lamp2/tub | Ratio /WT |
|---|---|---|---|---|---|
| WT | 1 | 14792832 | 24383736 | 0.607 | 1.00 |
| KO D1 | 2 | 19835064 | 21795768 | 0.910 | 1.50 |
| KO D2 | 3 | 14554152 | 24219504 | 0.601 | 0.99 |
| KO D1/D2 | 4 | 15007392 | 19417680 | 0.773 | 1.27 |
| KO ACOX1 | 5 | 15696648 | 25799256 | 0.608 | 1.00 |

C:\Users\MOUNIA\Desktop\New membranes Lamp2\Mounia Membrane Lamp2-8-bis-12-10-2021\XRS 2021-10-12 12hr 11min_Exposure_687.0sec.scn

C:\Users\MOUNIA\Desktop\New membranes Lamp2\Mounia Membrane Lamp2-8-bis-12-10-2021\Tubuline\XRS 2021-10-12 11hr 39min_Exposure_59.9sec.scn

12-10-2021
Samples
Mounia

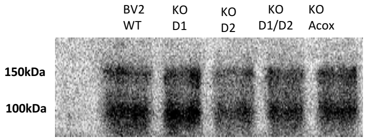


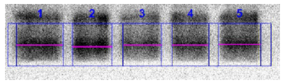


Membrane 10-bis

Exposure_691.1sec

Tubuline

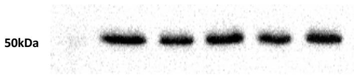


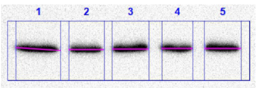


Exposure_59.3sec

## Densito analysis

| 12-10-2021 Samples | n° bande | CathB | Tubuline | Ratio CathB/tub | Ratio /WT |
|---|---|---|---|---|---|
| WT | 1 | 9274500 | 11315568 | 0.820 | 1.00 |
| KO D1 | 2 | 9244413 | 7627492 | 1.212 | 1.48 |
| KO D2 | 3 | 4883027 | 9942660 | 0.491 | 0.60 |
| KO D1/D2 | 4 | 6106368 | 7123440 | 0.857 | 1.05 |
| KO ACOX1 | 5 | 6816667 | 8907080 | 0.765 | 0.93 |

C:\Users\MOUNIA\Desktop\New membranes Lamp2\Mounia Membrane Lamp2 10-bis-12-10-2021\XRS 2021-10-12 12hr 55min_Exposure_691.1sec.scn

C:\Users\MOUNIA\Desktop\New membranes Lamp2\Mounia Membrane Lamp2 10-bis-12-10-2021\Tubuline\Tubuline revelation 2\XRS 2021-10-12 12hr 01min_Exposure_59.3sec.scn

*Mb selected for the publication*

12-10-2021
Samples
Mounia

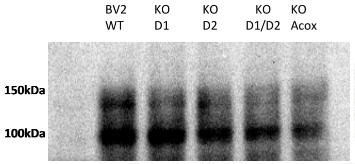


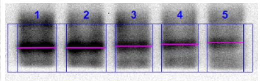


membrane 9 bis

Exposure_687.0sec

Tubuline

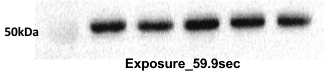


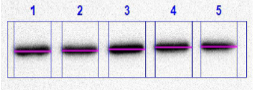


Exposure_59.9sec

## Densito analysis

| 12-10-2021 Samples | n° bande | Lamp2 | Tubuline | Ratio Lamp2/tub | Ratio /WT |
|---|---|---|---|---|---|
| WT | 1 | 20194104 | 22902066 | 0.882 | 1.00 |
| KO D1 | 2 | 28551744 | 20224038 | 1.412 | 1.60 |
| KO D2 | 3 | 14932759 | 27439920 | 0.544 | 0.62 |
| KO D1/D2 | 4 | 13640932 | 23239131 | 0.587 | 0.67 |
| KO ACOX1 | 5 | 6533036 | 18765999 | 0.348 | 0.39 |

C:\Users\MOUNIA\Desktop\New membranes Lamp2\Mounia Membrane Lamp2-9-bis-12-10-2021\XRS 2021-10-12 12hr 31min_Exposure_687.0sec.scn

C:\Users\MOUNIA\Desktop\New membranes Lamp2\Mounia Membrane Lamp2-9-bis-12-10-2021\Tubuline\XRS 2021-10-12 11hr 48min_Exposure_59.9sec.scn

| | Transcriptomique |
|---|---|
| | Moyenne ratio |
| WT | 1 |
| KO D1 | 0,56 |
| KO D2 | 1,03 |
| KO D1/D2 | 0,48 |
| KO ACOX1 | 0,87 |

| | Moyenne ratio | Ecart-type |
|---|---|---|
| WT | 1.000 | 0.000 |
| KO D1 | 1.527 | 0.065 |
| KO D2 | 0.736 | 0.221 |
| KO D1/D2 | 0.995 | 0.307 |
| KO ACOX1 | 0.777 | 0.333 |

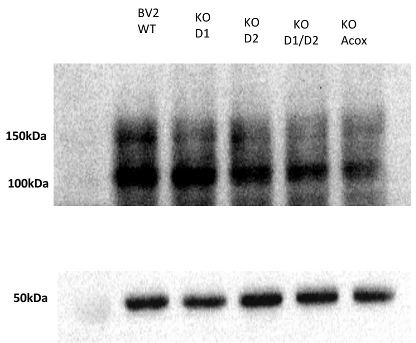


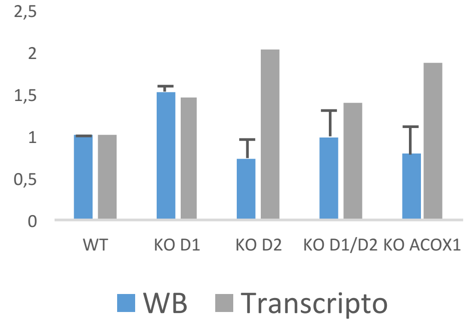

# Atp6v0d2

## Samples serie S1 (21/10/21)
## WB Catherine 28/11/2022 – Mb « A1/3 »

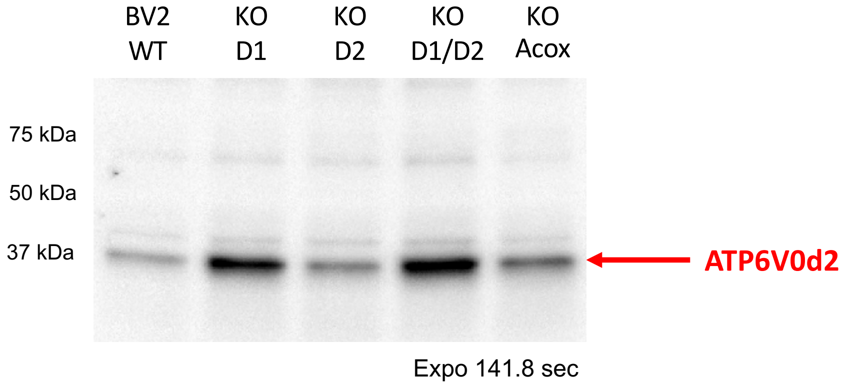


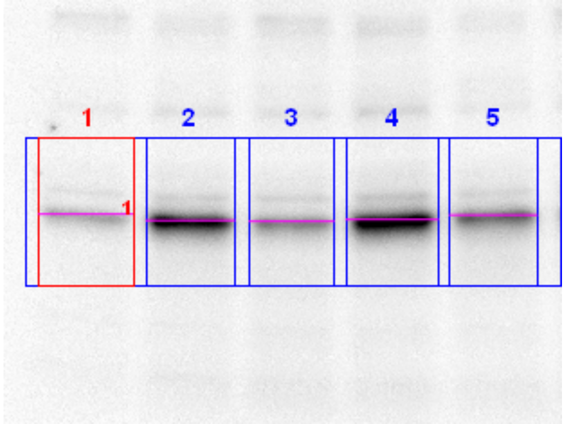


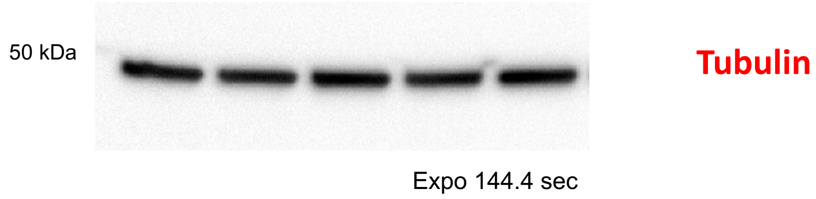


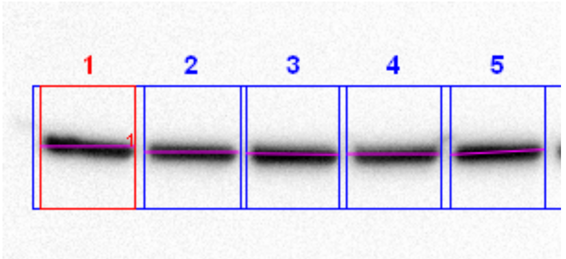


**ATP6v0d2**
MW : 40 kDa
Observed : 38 kDa

Antibody : Sigma #SAB2103221

4-20% Gradient Gel
30 µg/load
PVDF Transfer

## Densitometric analysis

| Série 1 - 21-10-2021 | ATP6V0d2 | Tubulin | Ratio ATP6v0d2/tub | Ratio /WT |
|---|---|---|---|---|
| WT | 24 166 905 | 78 766 422 | 0.307 | 1.00 |
| KO D1 | 76 845 384 | 72 136 722 | 1.065 | 3.47 |
| KO D2 | 33 795 775 | 83 127 456 | 0.407 | 1.33 |
| KO D1/D2 | 92 785 284 | 76 446 720 | 1.214 | 3.96 |
| KO Acox | 49 237 258 | 85 165 709 | 0.578 | 1.88 |

**Atp6v0d2**

# Samples serie S2 (27/10/21)
# WB Catherine 22/11/2022 – Mb « A2/3 »

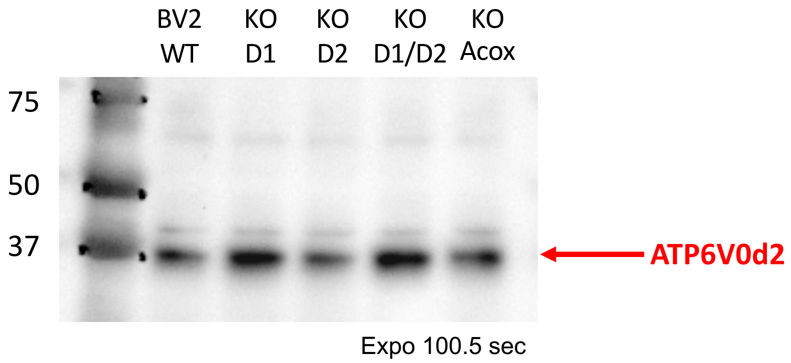


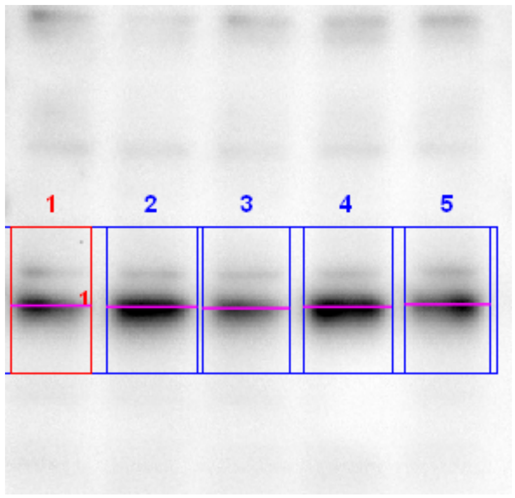


4-20% Gradient Gel
30 µg/load
PVDF Transfer

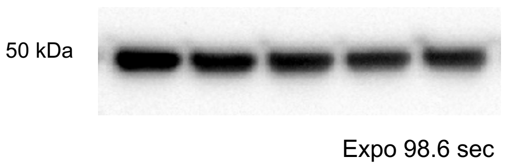


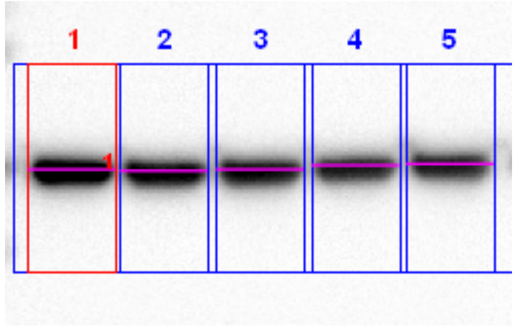


## Densitometric analysis

| Série 2 - 27-10-2021 | ATP6V0d2 | Tubulin | Ratio ATP6v0d2/tub | Ratio /WT |
|---|---|---|---|---|
| **WT** | 31 403 227 | 59 390 528 | 0.529 | **1.00** |
| **KO D1** | 50 318 664 | 49 225 472 | 1.022 | **1.93** |
| **KO D2** | 31 909 350 | 46 981 888 | 0.679 | **1.28** |
| **KO D1/D2** | 53 228 485 | 41 566 144 | 1.281 | **2.42** |
| **KO Acox** | 38 270 505 | 42 272 448 | 0.905 | **1.71** |

**Atp6v0d2**

## Samples serie S3 (28/10/21)
## WB Catherine 28/11/2022 – Mb « A1/3 »

*Mb selected for the publication*

BV2 WT | KO D1 | KO D2 | KO D1/D2 | KO Acox

75 kDa
50 kDa
37 kDa

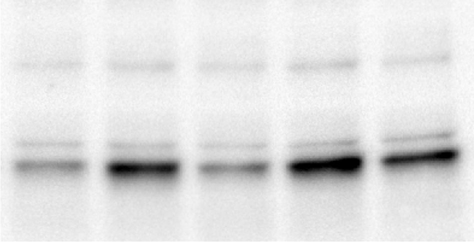

ATP6V0d2

Expo 129.7 sec

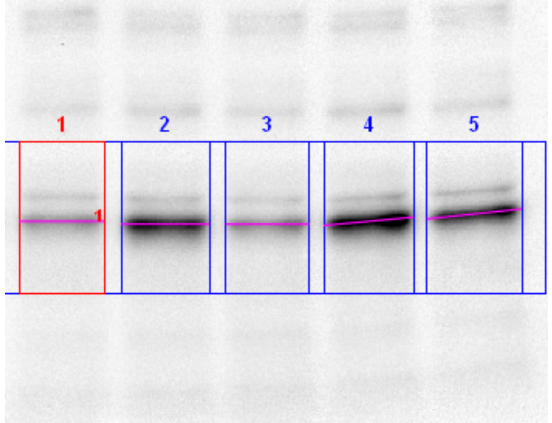


4-20% Gradient Gel
30 µg/load
PVDF Transfer

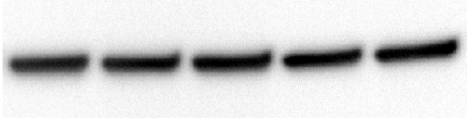

Tubulin

Expo 144.4 sec

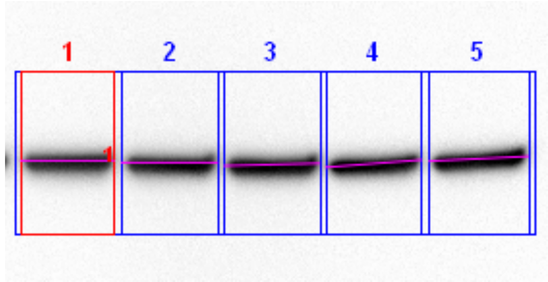


## Densitometric analysis

| Série 3 - 28-10-2021 | ATP6V0d2 | Tubulin | Ratio ATP6v0d2/tub | Ratio KO/WT |
|---|---|---|---|---|
| WT | 29 913 171 | 82 577 167 | 0.362 | 1.00 |
| KO D1 | 70 492 416 | 84 742 000 | 0.832 | 2.30 |
| KO D2 | 30 864 743 | 90 564 111 | 0.341 | 0.94 |
| KO D1/D2 | 86 160 228 | 86 535 207 | 0.996 | 2.75 |
| KO Acox | 65 967 586 | 89 789 735 | 0.735 | 2.03 |

# Atp6v0d2

| | Transcriptomic |
|---|---|
| | Ratio Mean |
| WT | 1.0 |
| KO D1 | 7.6 |
| KO D2 | 5.6 |
| KO D1/D2 | 5.0 |
| KO Acox | 4.2 |

| Mb A1/3 S1 - A 2/3 S2 - A1/3 S3 | | |
|---|---|---|
| | Ratio Mean | Ratio SD |
| WT | 1 | 0 |
| KO D1 | 2.57 | 0.80 |
| KO D2 | 1.18 | 0.21 |
| KO D1/D2 | 3.04 | 0.81 |
| KO Acox | 1.87 | 0.16 |

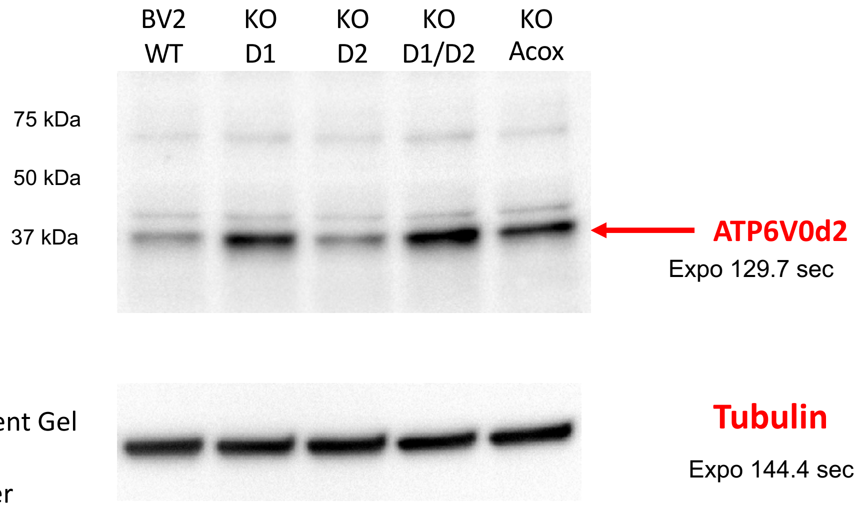


4-20% Gradient Gel
30 µg/load
PVDF Transfer

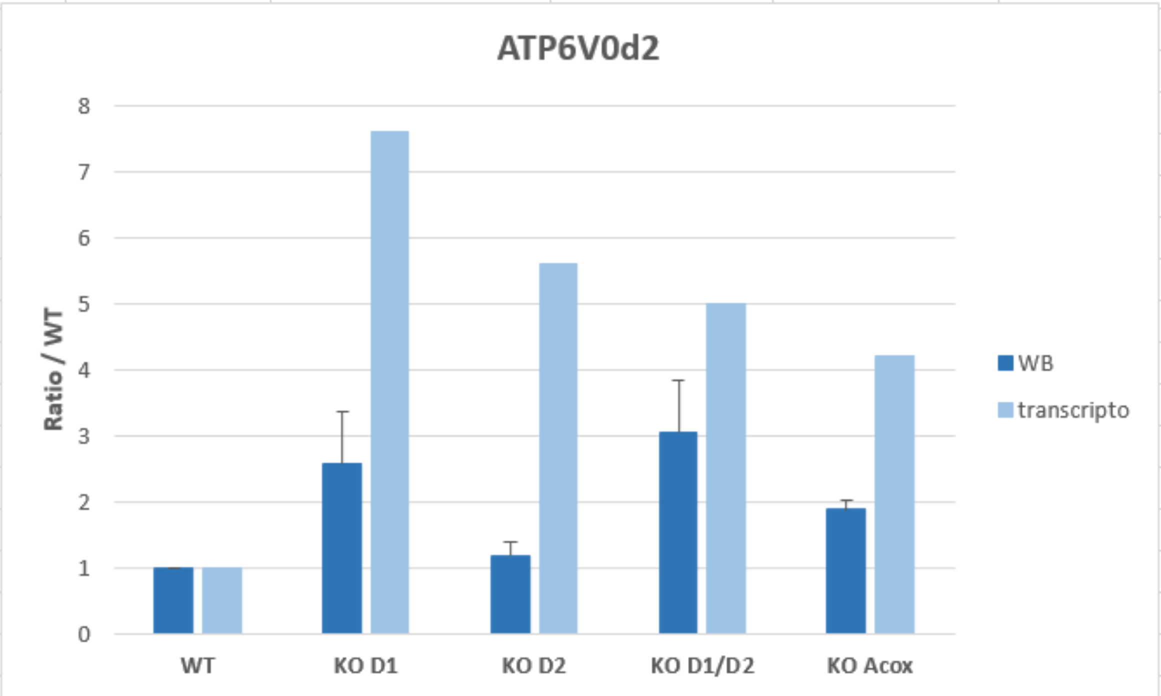

# Atp6v1b2

## Samples serie NS2
## WB Ali – Mb55

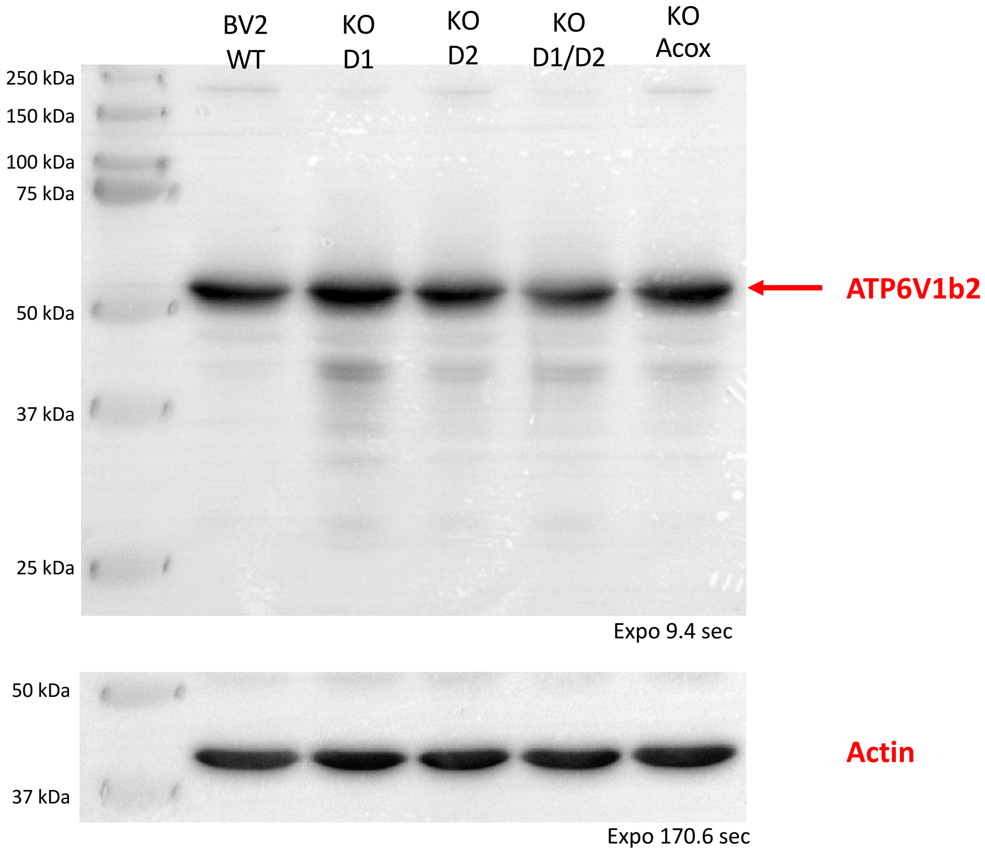


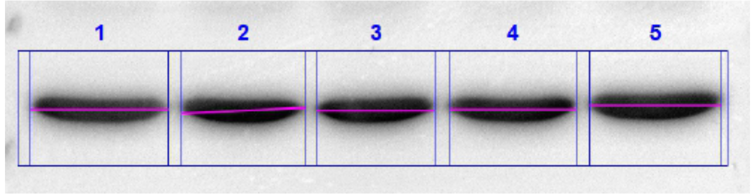


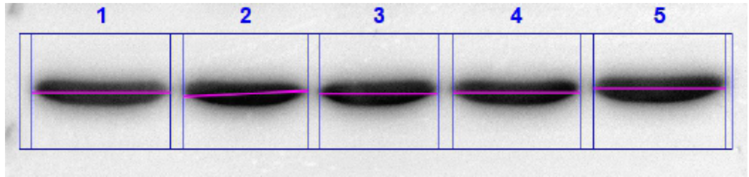


**ATP6v1b2**
MW : 56 kDa

Antibody : Abcam # ab73404

12% Gel
30 µg/load
PVDF Transfer

## Densitometric analysis

| NS2 18/05/2021 | ATP6V1B2 | Actin | Ratio ATP6V1B2/tub | Ratio /WT |
|---|---|---|---|---|
| **WT** | 135,008,118 | 113,887,488 | 1.185 | **1.00** |
| **KO D1** | 144,064,743 | 117,253,905 | 1.229 | **1.04** |
| **KO D2** | 121,386,804 | 109,782,554 | 1.106 | **0.93** |
| **KO D1/D2** | 103,206,404 | 119,362,222 | 0.865 | **0.73** |
| **KO Acox** | 137,876,462 | 129,153,188 | 1.068 | **0.90** |

# Atp6v1b2

## Samples serie NS3
## WB Ali – Mb56

*Mb selected for the publication*

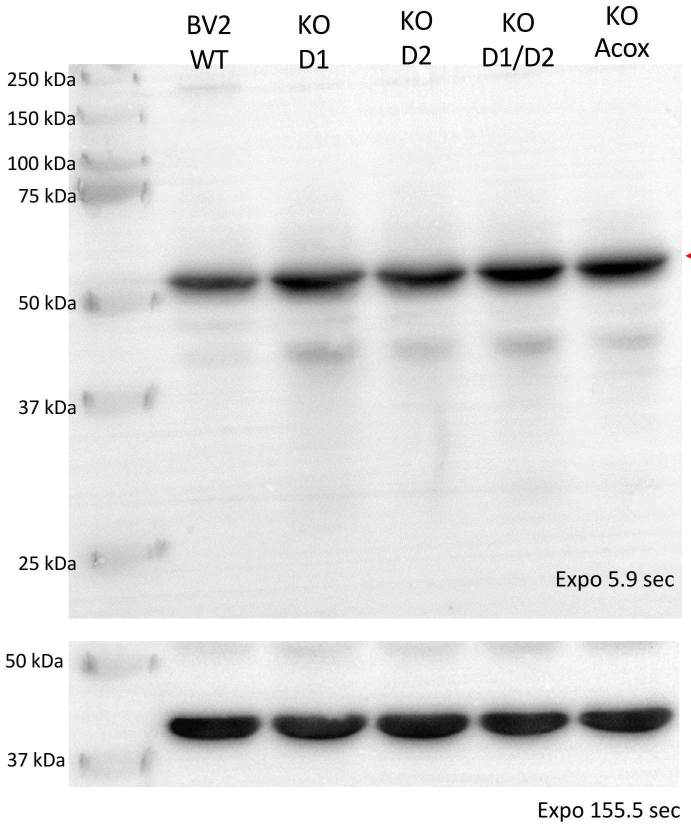


ATP6V1b2

Actin

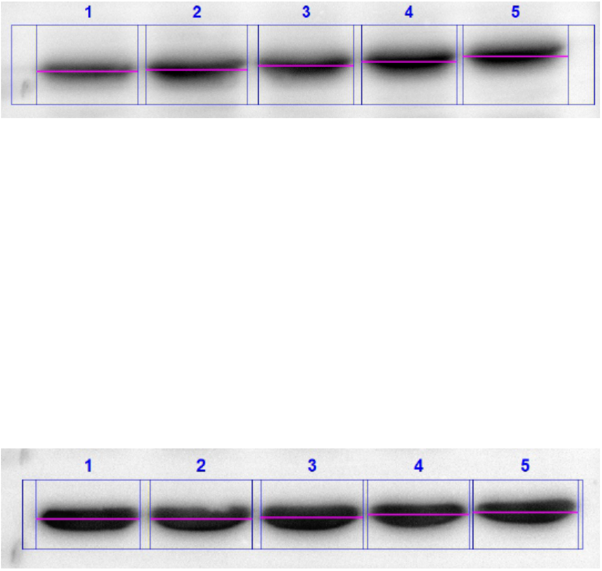


12% Gel
30 µg/load
PVDF Transfer

## Densitometric analysis

| NS3 18/05/2021 | ATP6V1B2 | Actin | Ratio ATP6V1B2/tub | Ratio /WT |
|---|---|---|---|---|
| **WT** | 100,369,900 | 133,759,539 | 0.750 | **1.00** |
| **KO D1** | 123,827,456 | 125,366,265 | 0.988 | **1.32** |
| **KO D2** | 114,960,440 | 140,472,224 | 0.818 | **1.09** |
| **KO D1/D2** | 131,733,219 | 130,434,930 | 1.010 | **1.35** |
| **KO Acox** | 130,342,828 | 133,204,812 | 0.979 | **1.30** |

# Atp6v1b2

## Samples serie NS7
## WB Ali – Mb57

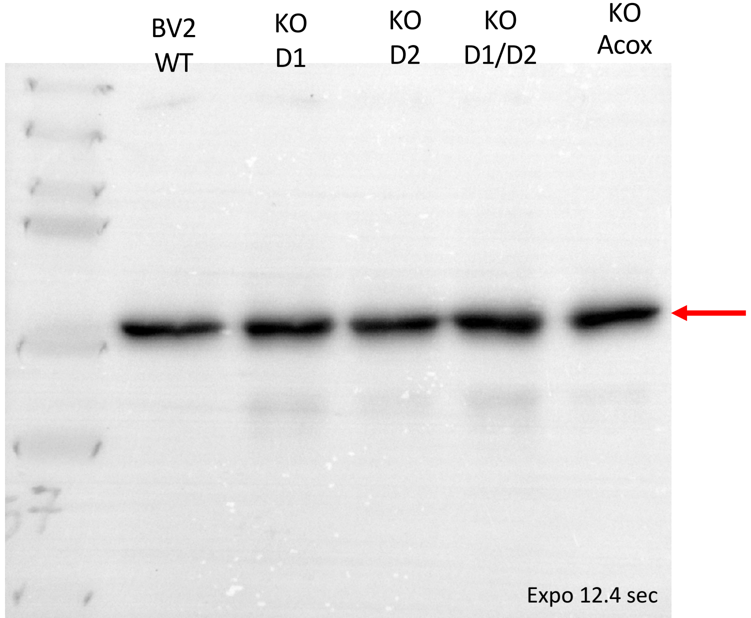


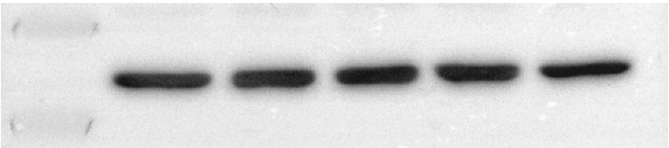


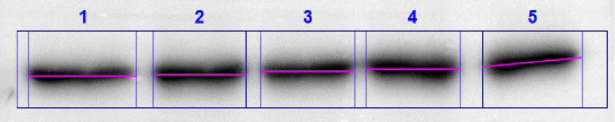


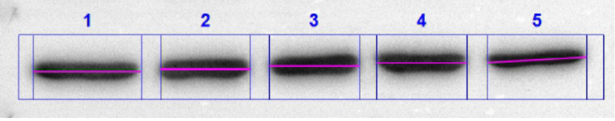


10% Gel
30 µg/load
PVDF Transfer

## Densitometric analysis

| NS7 04/06/2021 | ATP6V1B2 | Actin | Ratio ATP6V1B2/tub | Ratio /WT |
|---|---|---|---|---|
| **WT** | 107,810,518 | 111,720,222 | 0.965 | **1.00** |
| **KO D1** | 110,016,830 | 105,092,559 | 1.047 | **1.08** |
| **KO D2** | 104,381,613 | 111,269,808 | 0.938 | **0.97** |
| **KO D1/D2** | 126,103,044 | 105,896,700 | 1.191 | **1.23** |
| **KO Acox** | 112,339,370 | 101,306,667 | 1.109 | **1.15** |

# Atp6v1b2

| | Transcriptomique Moyenne Ratio |
|---|---|
| **WT** | 1 |
| **KO D1** | 2.51 |
| **KO D2** | 2.22 |
| **KO D1/D2** | 1.69 |
| **KO Acox** | 2.07 |

| Toutes les Mb | | |
|---|---|---|
| | **Moyenne Ratio** | **Ecart-type Ratio** |
| **WT** | 1 | 0 |
| **KO D1** | 1.15 | 0.15 |
| **KO D2** | 1.00 | 0.08 |
| **KO D1/D2** | 1.10 | 0.33 |
| **KO Acox** | 1.12 | 0.20 |

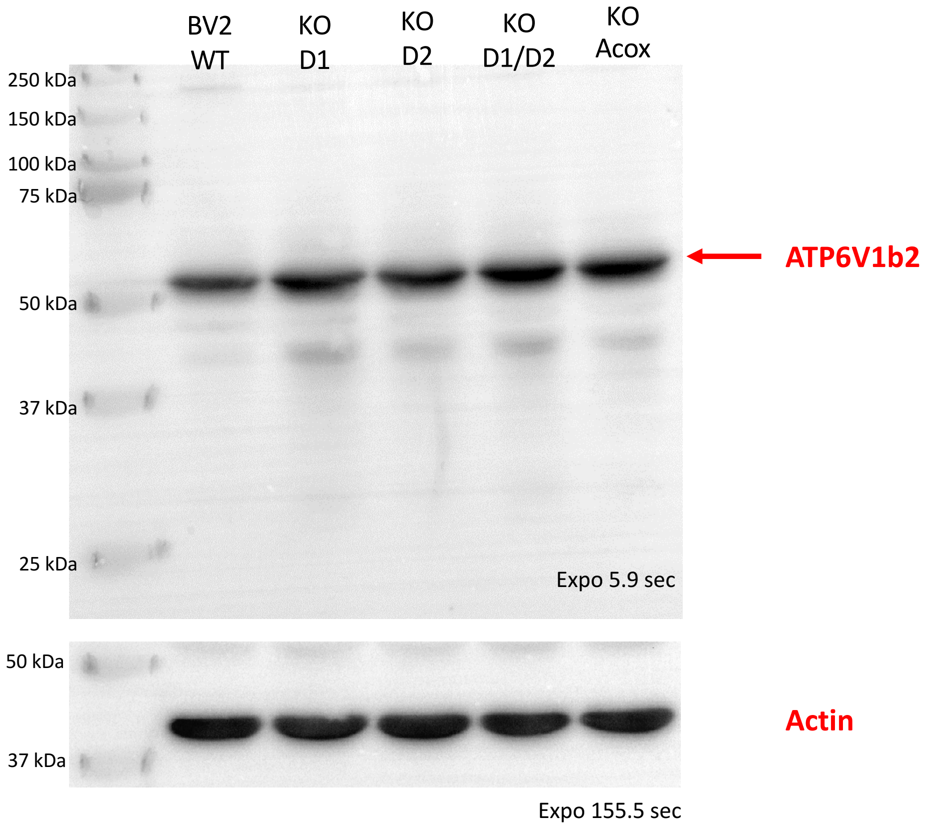


12% Gel
30 µg/load
PVDF Transfer

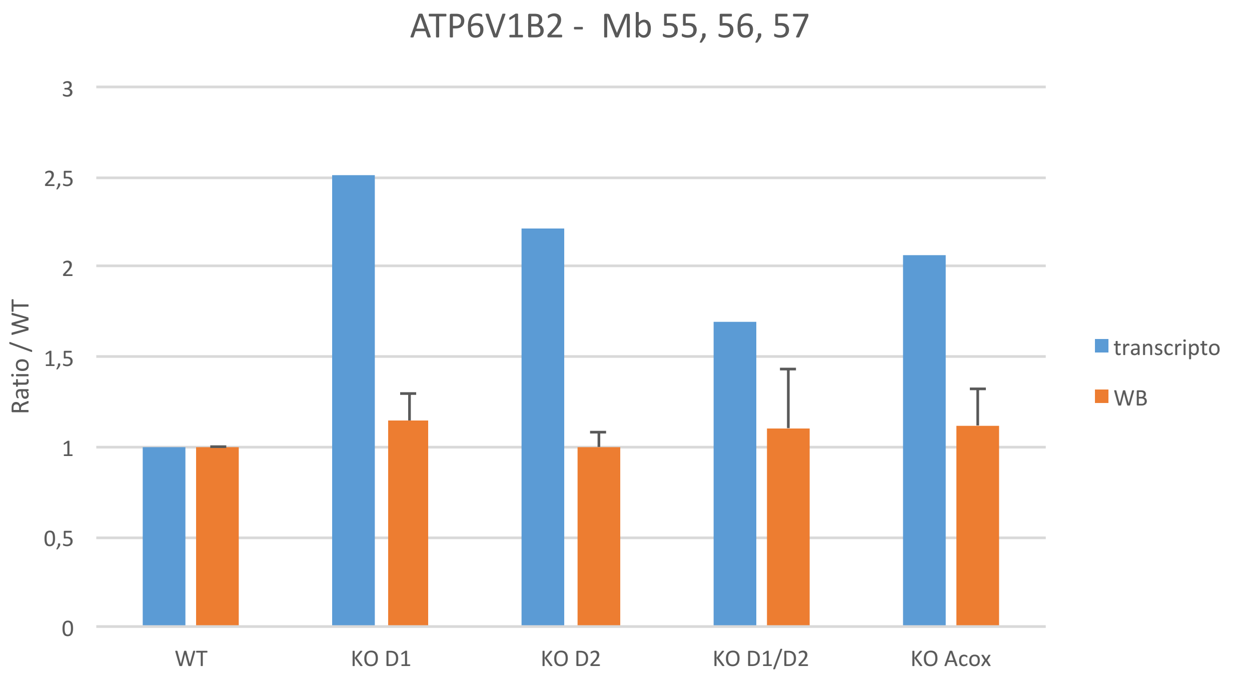

**Grn**

*Mb selected for the publication*

## 9/12/2020
## Samples serie 5

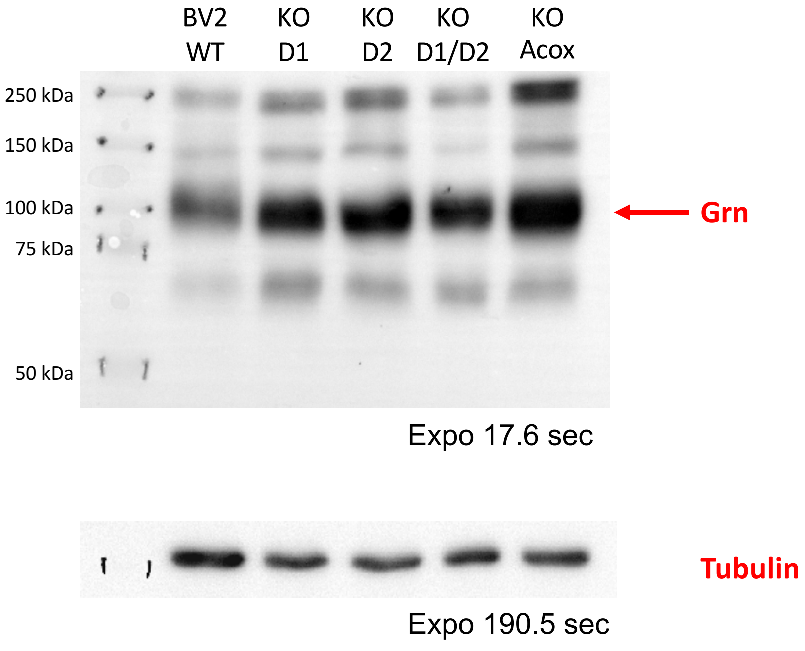


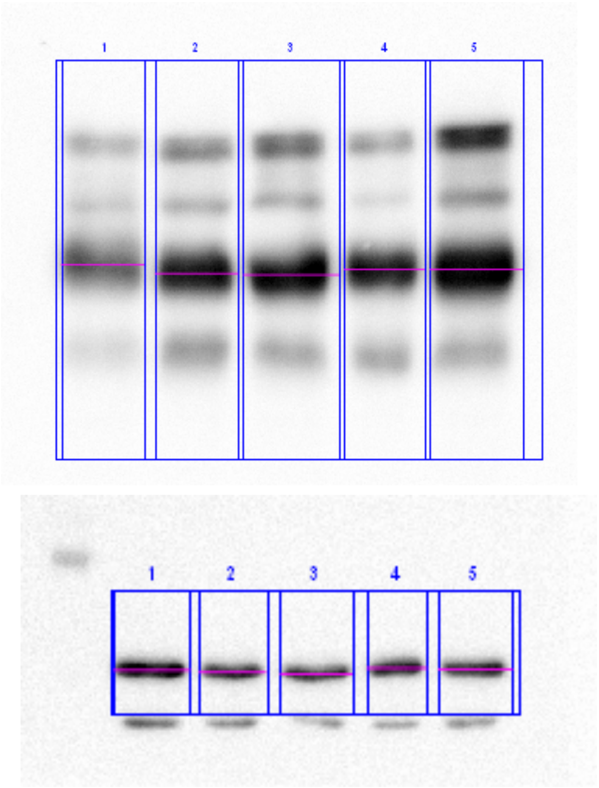


**Grn**
MW : 63 kDa
Observed : 100 kDa

Antibody : Abcam # ab187070

8% Gel
30 µg/load
PVDF Transfer

## Densitometric analysis

| Série 5 - 9/12/2020 | GRN | Tubuline | Ratio GRN/tub | Ratio /WT |
|---|---|---|---|---|
| WT | 199 919 248 | 50 604 825 | 3.951 | 1.00 |
| KO D1 | 251 904 512 | 31 603 506 | 7.971 | 2.02 |
| KO D2 | 307 851 973 | 31 481 116 | 9.779 | 2.48 |
| KO D1/D2 | 239 396 160 | 30 886 028 | 7.751 | 1.96 |
| KO Acox | 351 029 905 | 35 116 431 | 9.996 | 2.53 |

**Grn**

## NS1
## Samples
## 23/04/2021

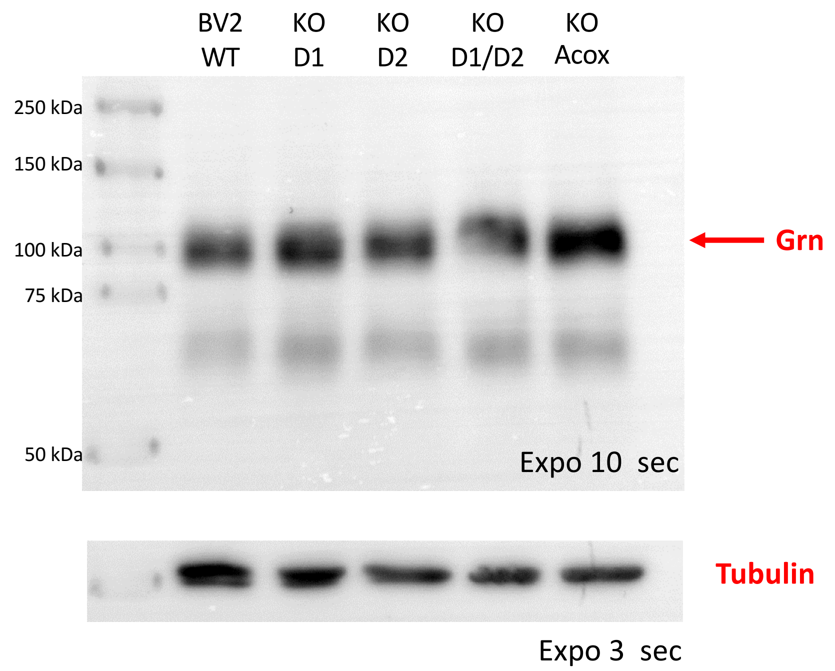


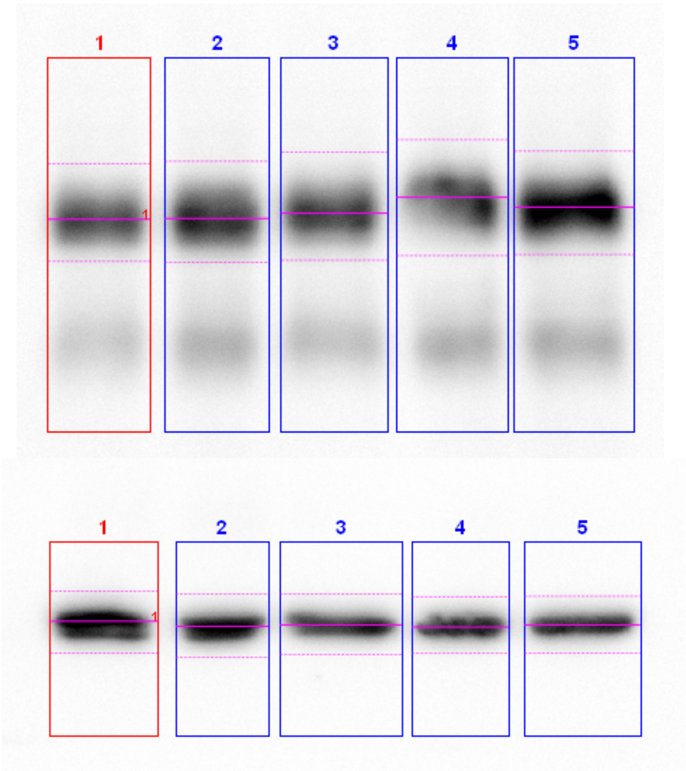


8% Gel
30 µg/load
PVDF Transfer

## Densitometric analysis

| Série - 25/02/2021 | GRN | Tubuline | Ratio GRN/tub | Ratio /WT |
|---|---|---|---|---|
| WT | 233 024 175 | 194 779 263 | 1.196 | 1.00 |
| KO D1 | 286 170 048 | 144 192 244 | 1.985 | 1.66 |
| KO D2 | 271 671 891 | 146 415 154 | 1.855 | 1.55 |
| KO D1/D2 | 272 572 932 | 122 337 864 | 2.228 | 1.86 |
| KO Acox | 405 764 820 | 137 063 262 | 2.960 | 2.47 |

# Grn

## NS3 Samples 30/04/2021

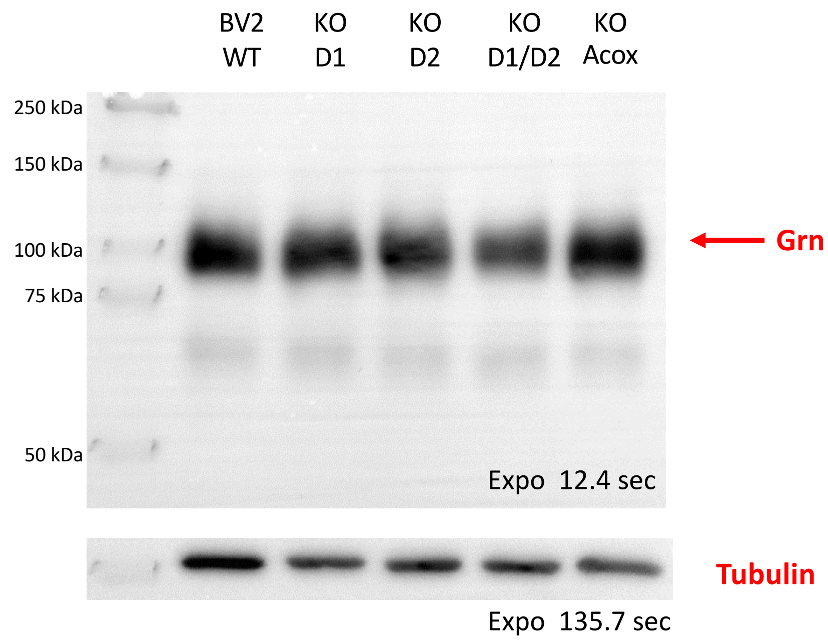


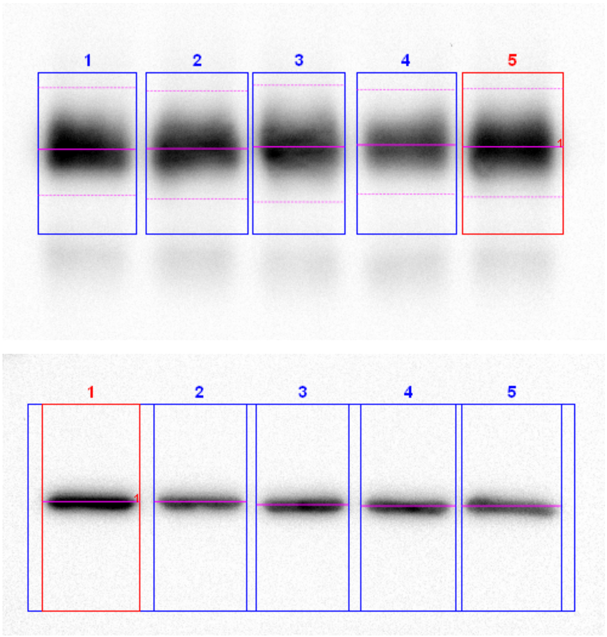


8% Gel
30 µg/load
PVDF Transfer

## Densitometric analysis

| Série - 25/02/2021 | GRN | Tubuline | Ratio GRN/tub | Ratio /WT |
|---|---|---|---|---|
| WT | 459 182 878 | 201 925 393 | 2.274 | 1.00 |
| KO D1 | 445 833 955 | 128 036 594 | 3.482 | 1.53 |
| KO D2 | 415 956 280 | 143 449 750 | 2.900 | 1.28 |
| KO D1/D2 | 349 668 852 | 139 179 969 | 2.512 | 1.10 |
| KO Acox | 494 113 170 | 130 731 040 | 3.780 | 1.66 |

# Grn

| | Transcriptomique Moyenne Ratio |
|---|---|
| WT | 1 |
| KO D1 | 2,12 |
| KO D2 | 2,30 |
| KO D1/D2 | 2,23 |
| KO Acox | 2,20 |

| Mb 4, 40, 47 | | |
|---|---|---|
| | Moyenne Ratio | Ecart-type Ratio |
| WT | 1,00 | 0,00 |
| KO D1 | 1,74 | 0,25 |
| KO D2 | 1,77 | 0,63 |
| KO D1/D2 | 1,64 | 0,47 |
| KO Acox | 2,22 | 0,49 |

**9/12/2020**
**Samples serie 5**

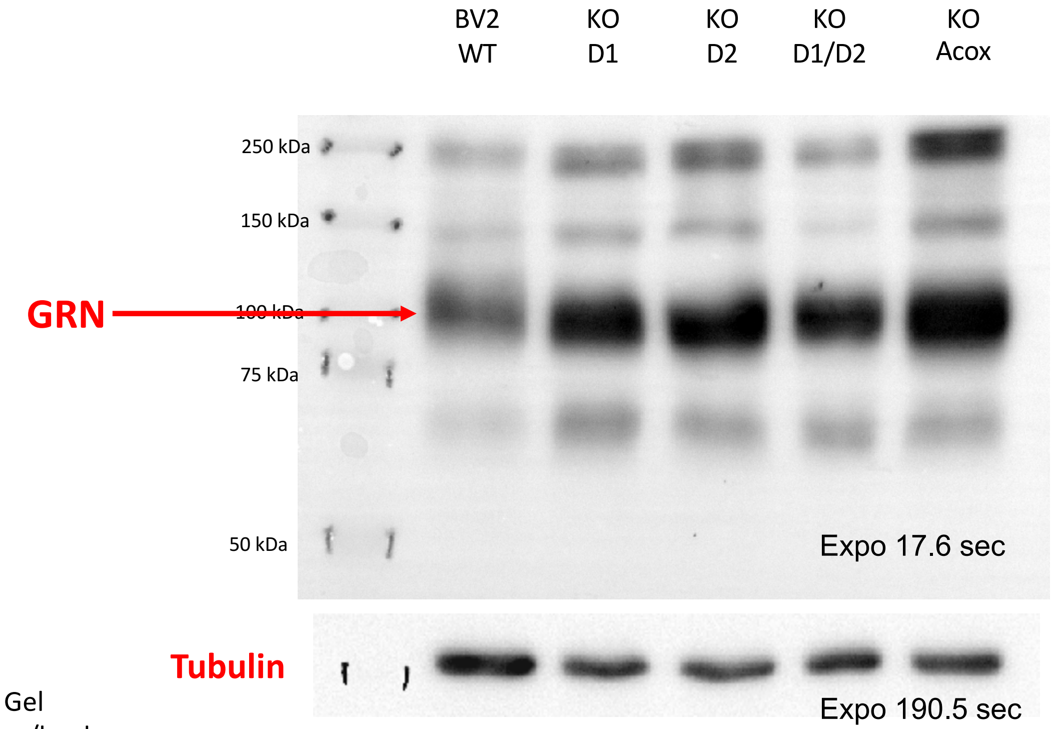


8% Gel
30 µg/load
PVDF Transfer

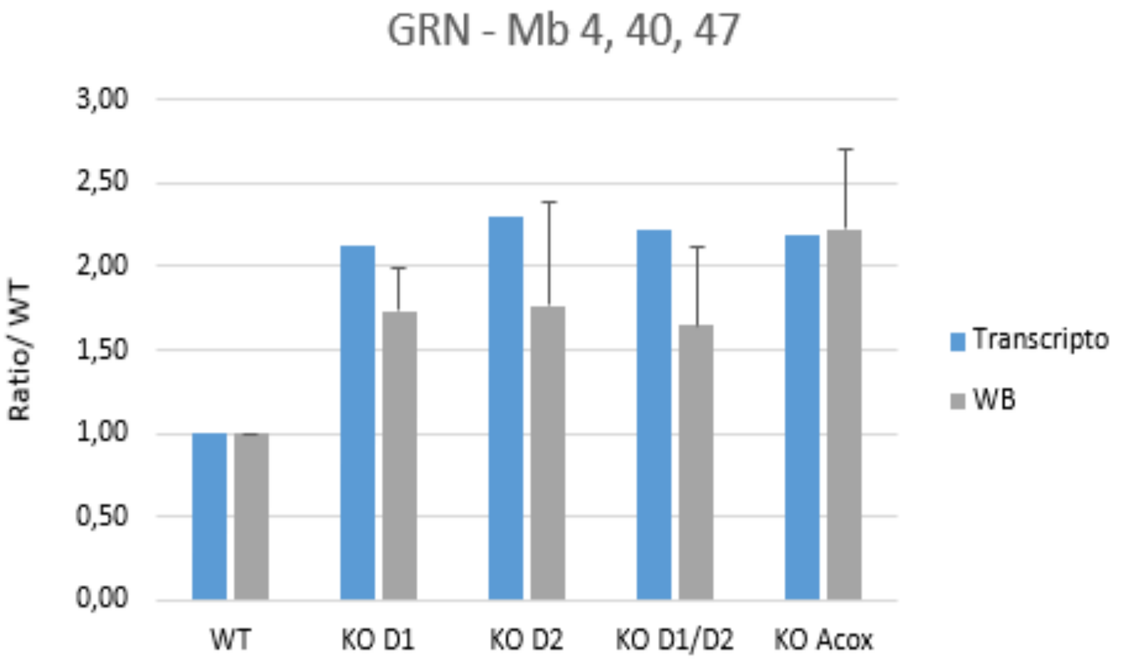

**Lgals3**

# 9-10-11/12/2020
# Catherine - Samples serie 5

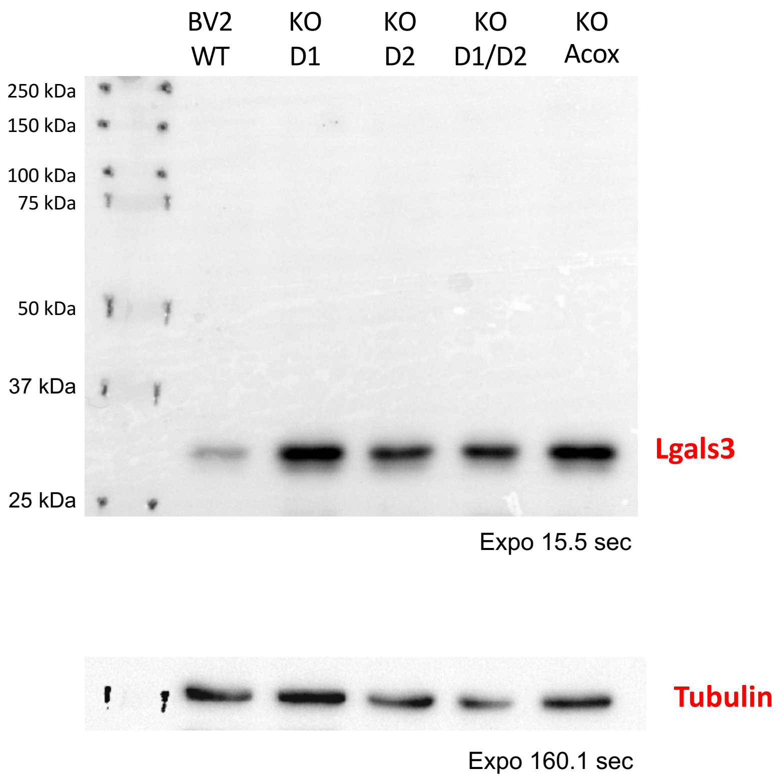


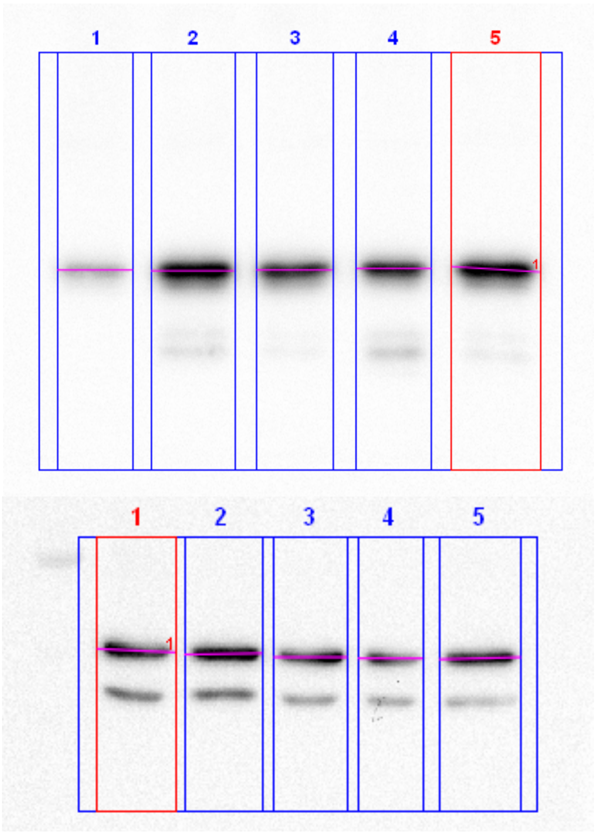


**Lgals3**
MW : 27 kDa

Antibody : Abcam # ab190167

10% Gel
30 µg/load
PVDF Transfer

## Densitometric analysis

| Série 5 - 10/12/2020 | LGALS3 | Tubuline | Ratio LGALS3/tub | Ratio KO/WT |
|---|---|---|---|---|
| WT | 29 152 926 | 28 172 871 | 1.035 | 1.00 |
| KO D1 | 127 930 880 | 34 931 432 | 3.662 | 3.54 |
| KO D2 | 88 452 768 | 24 281 336 | 3.643 | 3.52 |
| KO D1/D2 | 79 641 631 | 14 773 128 | 5.391 | 5.21 |
| KO Acox | 126 739 165 | 28 054 140 | 4.518 | 4.37 |

# Lgals3

## 14-15/12/2020
## Ali - Samples serie 6

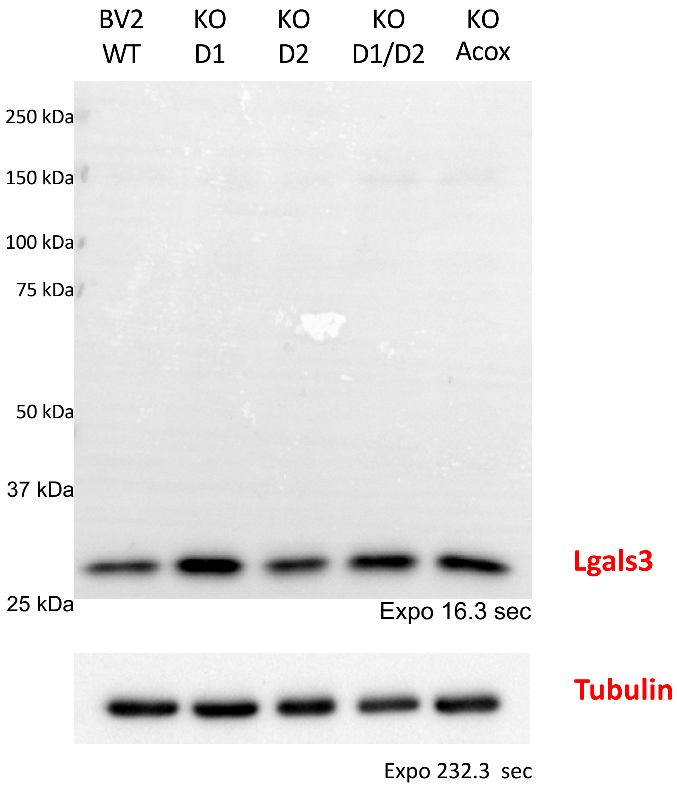


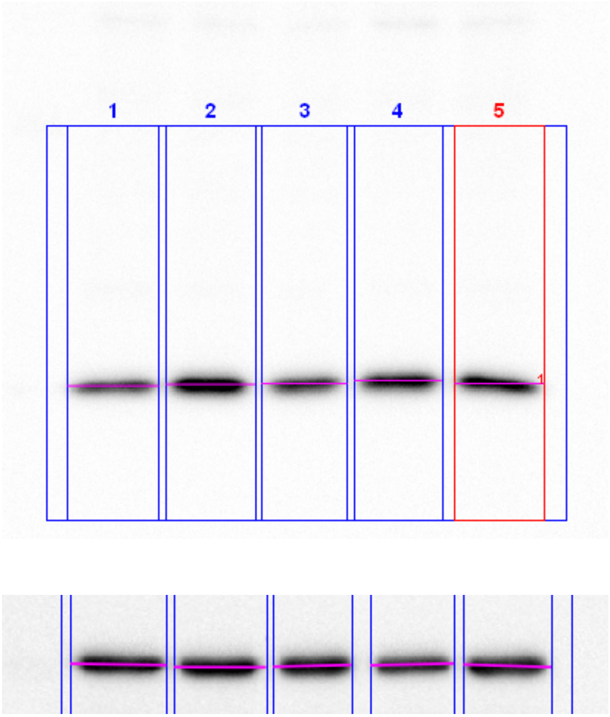


10% Gel
30 µg/load
PVDF Transfer

## Densitometric analysis

| Série 6 - 15/12/2020 | LGALS3 | Tubuline | Ratio LGALS3/tub | Ratio KO/WT |
|---|---|---|---|---|
| **WT** | 53 494 189 | 40 214 166 | 1.330 | **1.00** |
| **KO D1** | 92 607 788 | 39 055 632 | 2.371 | **1.78** |
| **KO D2** | 55 305 600 | 33 966 240 | 1.628 | **1.22** |
| **KO D1/D2** | 74 585 526 | 28 632 960 | 2.605 | **1.96** |
| **KO Acox** | 73 814 949 | 36 035 811 | 2.048 | **1.54** |

Lgals3

*Mb selected for the publication*

## 11/02/2021
## Samples serie Ali

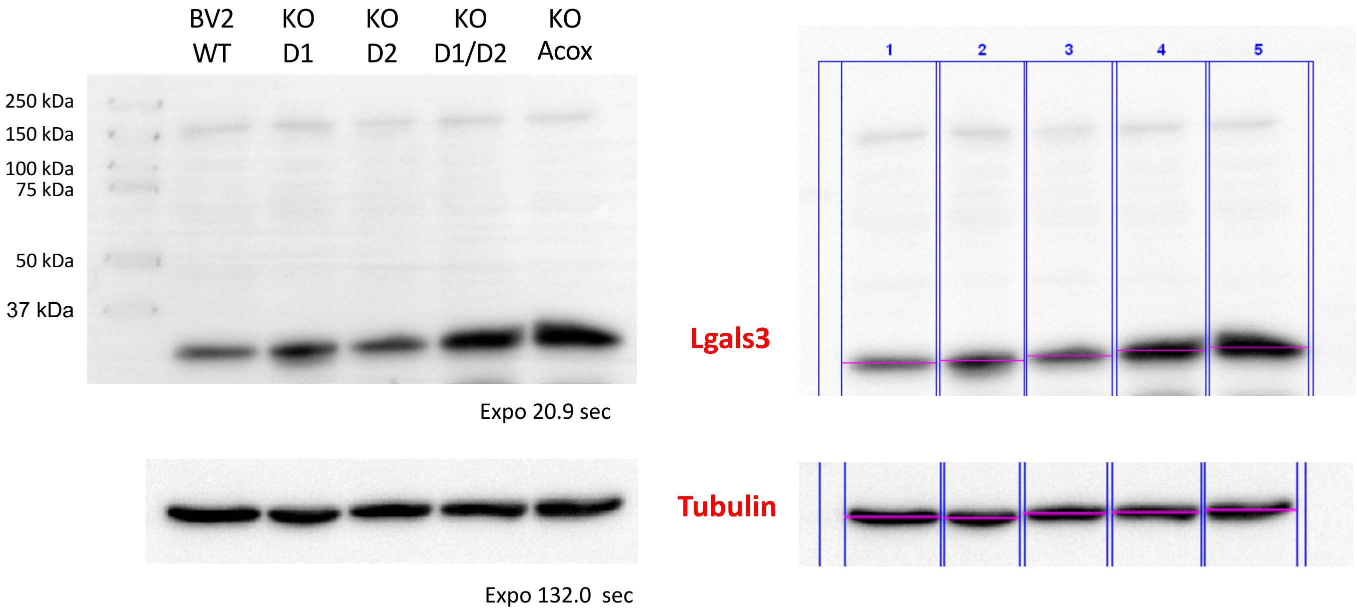


10% Gel
30 µg/load
PVDF Transfer

## Densitometric analysis

| Série 10 - 12/02/2021 | LGALS3 | Tubuline | Ratio LGALS3/tub | Ratio KO/WT |
|---|---|---|---|---|
| WT | 109 097 104 | 55 657 559 | 1.960 | 1.00 |
| KO D1 | 140 387 306 | 37 918 400 | 3.702 | 1.89 |
| KO D2 | 120 055 650 | 42 941 736 | 2.796 | 1.43 |
| KO D1/D2 | 209 989 127 | 42 344 692 | 4.959 | 2.53 |
| KO Acox | 263 175 968 | 46 488 735 | 5.661 | 2.89 |

# Lgals3

| | Transcriptomique |
|---|---|
| | Moyenne Ratio |
| WT | 1,00 |
| KO D1 | 2,93 |
| KO D2 | 2,91 |
| KO D1/D2 | 3,27 |
| KO Acox | 3,05 |

| | Mb 6, 10, 20 | |
|---|---|---|
| | Moyenne Ratio | Ecart-type Ratio |
| WT | 1 | 0 |
| KO D1 | 2,40 | 0,98 |
| KO D2 | 2,06 | 1,27 |
| KO D1/D2 | 3,23 | 1,74 |
| KO Acox | 2,93 | 1,41 |

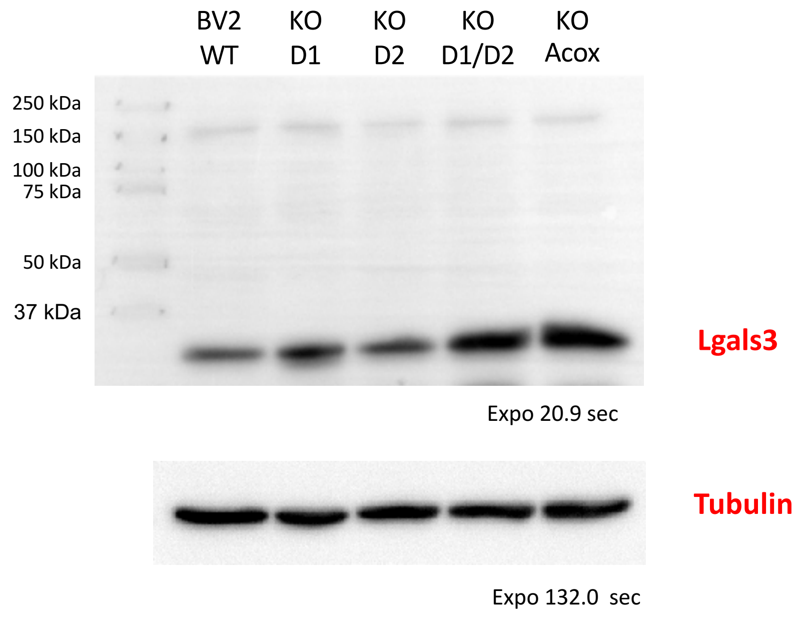


10% Gel
30 µg/load
PVDF Transfer

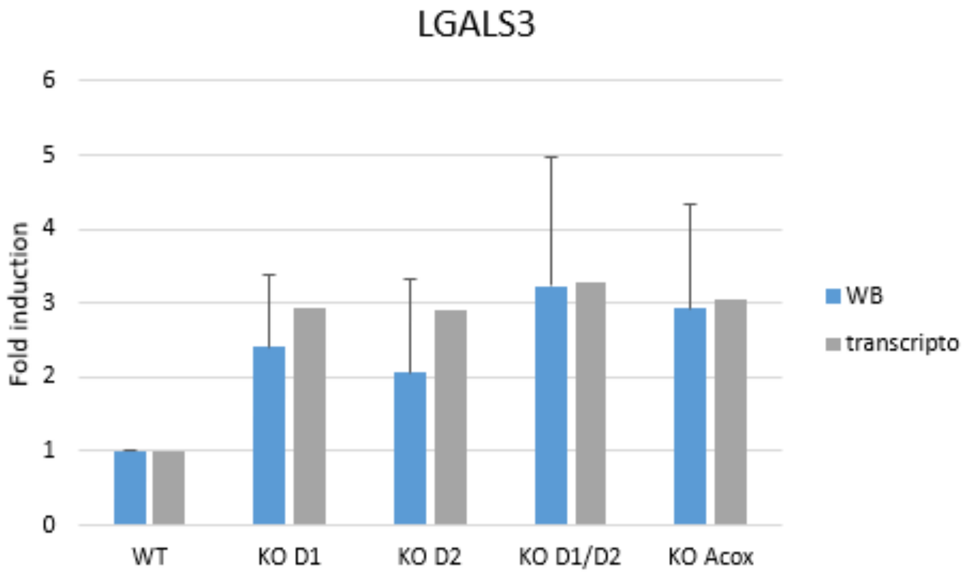

# Atg13

## 3-4/12/2020
## Samples serie 4

*Mb selected for the publication*

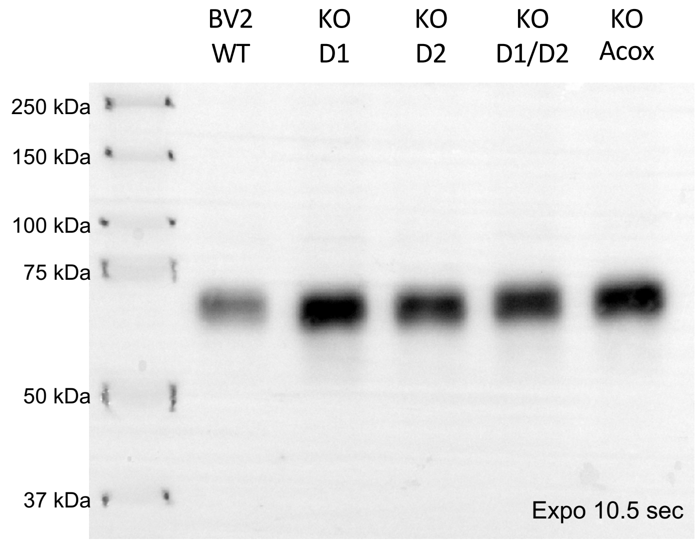


**Atg13**

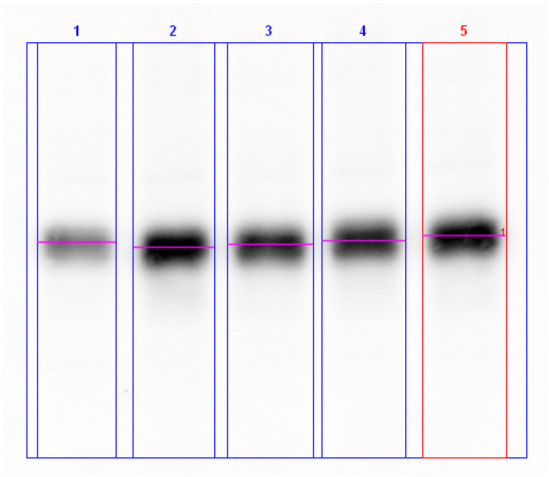


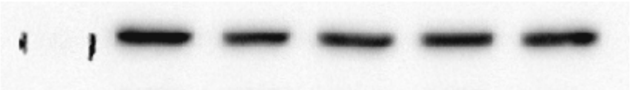

Expo 166.2 sec

**Tubulin**

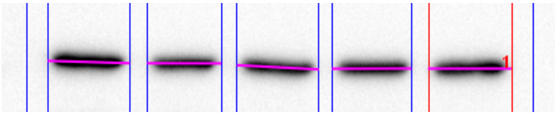

**Atg13**
MW : 56 kDa
Observed : 70 kDa

Antibody : Abcam # ab201467

8% Gel
30 µg/load
PVDF Transfer

## Densitometric analysis

| Série 4 - 9/12/2020 | ATG 13 | Tubuline | RatioATG 13/tub | Ratio /WT |
|---|---|---|---|---|
| WT | 81 593 706 | 34 162 821 | 2.388 | 1.00 |
| KO D1 | 182 440 932 | 22 751 493 | 8.019 | 3.36 |
| KO D2 | 151 664 877 | 26 011 377 | 5.831 | 2.44 |
| KO D1/D2 | 153 109 980 | 26 308 202 | 5.820 | 2.44 |
| KO Acox | 178 369 758 | 29 110 976 | 6.127 | 2.57 |

# Atg13

## 9-10-11/12/2020
## Catherine - Samples serie 5

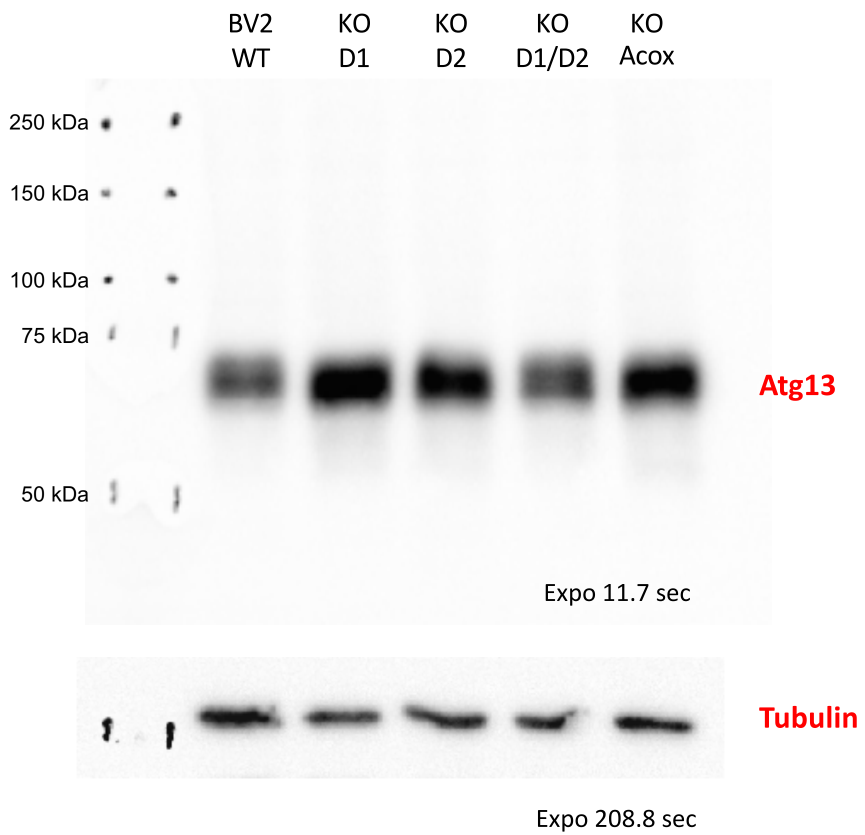


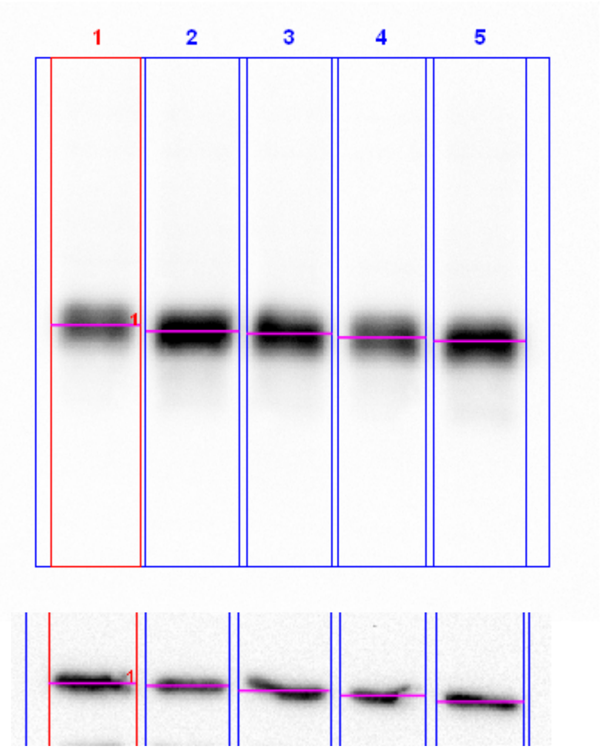


8% Gel
30 µg/load
PVDF Transfer

## Densitometric analysis

| Série 5 - 10/12/2020 | ATG 13 | Tubuline | RatioATG 13/tub | Ratio KO/WT |
|---|---|---|---|---|
| WT | 49 849 056 | 44 252 383 | 1.126 | 1.00 |
| KO D1 | 89 755 575 | 29 436 718 | 3.049 | 2.71 |
| KO D2 | 72 644 097 | 34 149 224 | 2.127 | 1.89 |
| KO D1/D2 | 54 758 111 | 26 027 559 | 2.104 | 1.87 |
| KO Acox | 77 444 922 | 34 057 422 | 2.274 | 2.02 |

**Atg13**

# 14-15/12/2020
# Ali - Samples serie 6

| BV2 WT | KO D1 | KO D2 | KO D1/D2 | KO Acox |
|---|---|---|---|---|

250 kDa
150 kDa
100 kDa
75 kDa
50 kDa

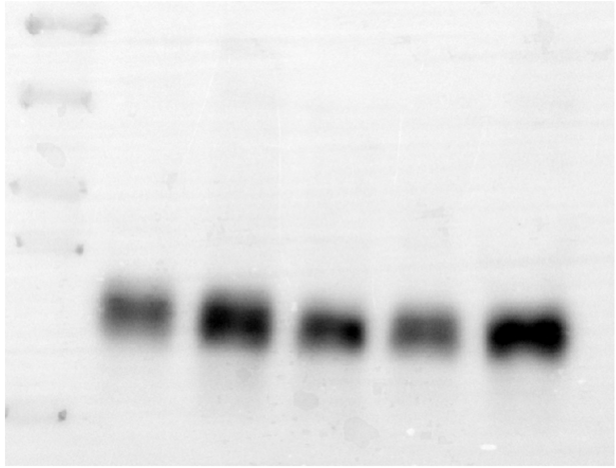

Expo 15.1 sec

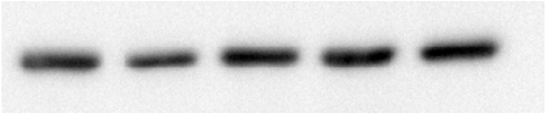

Expo 259.0 sec

Atg13

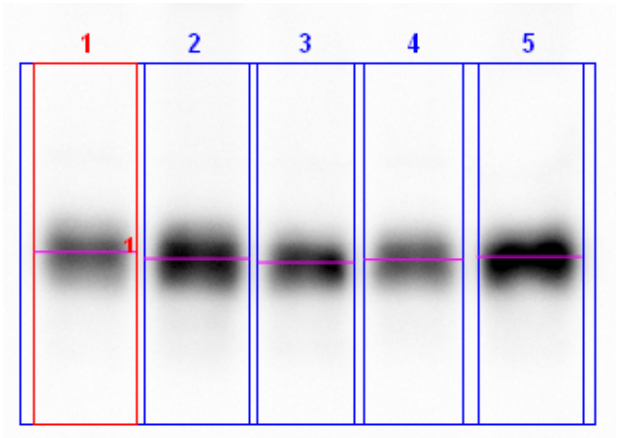


Tubulin

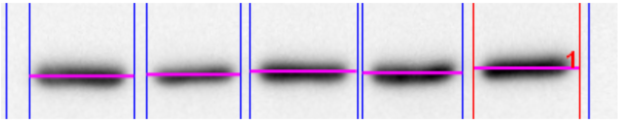

8% Gel
30 µg/load
PVDF Transfer

## Densitometric analysis

| Série 6 - 15/12/2020 | ATG 13 | Tubuline | RatioATG 13/tub | Ratio KO/WT |
|---|---|---|---|---|
| WT | 107 620 279 | 31 428 540 | 3.424 | 1.00 |
| KO D1 | 158 785 278 | 22 618 434 | 7.020 | 2.05 |
| KO D2 | 114 754 180 | 31 807 908 | 3.608 | 1.05 |
| KO D1/D2 | 93 547 064 | 31 835 016 | 2.938 | 0.86 |
| KO Acox | 176 284 130 | 35 436 180 | 4.975 | 1.45 |

# Atg13

| | Transcriptomique |
|---|---|
| | Moyenne Ratio |
| WT | 1,00 |
| KO D1 | 2,24 |
| KO D2 | 2,83 |
| KO D1/D2 | 2,33 |
| KO Acox | 2,35 |

| Mb 3bis, 7, 11 | | |
|---|---|---|
| | Moyenne Ratio | Ecart-type Ratio |
| WT | 1 | 0 |
| KO D1 | 2,70 | 0,65 |
| KO D2 | 1,79 | 0,70 |
| KO D1/D2 | 1,72 | 0,80 |
| KO Acox | 2,01 | 0,56 |

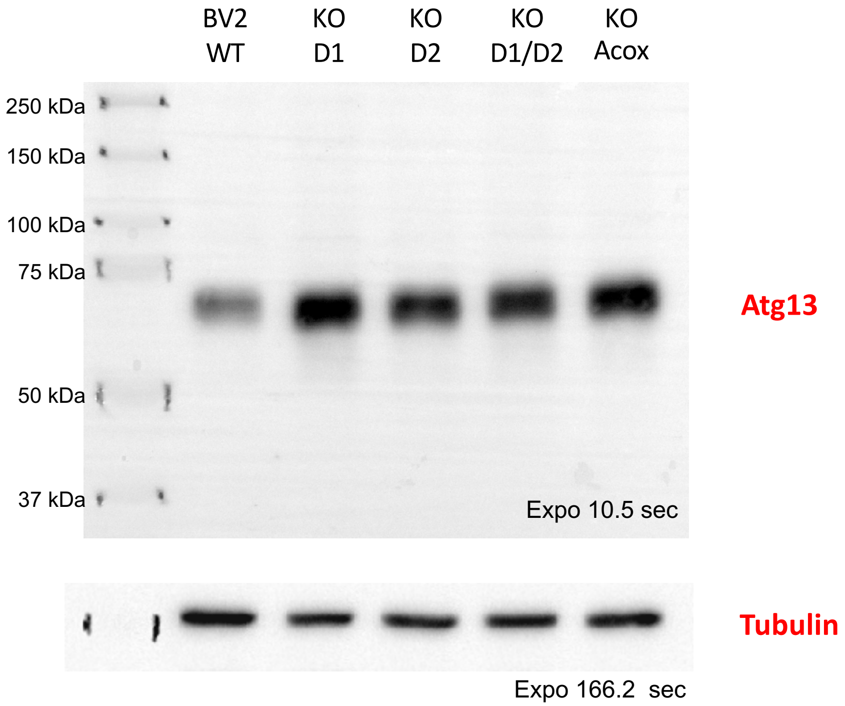


8% Gel
30 µg/load
PVDF Transfer

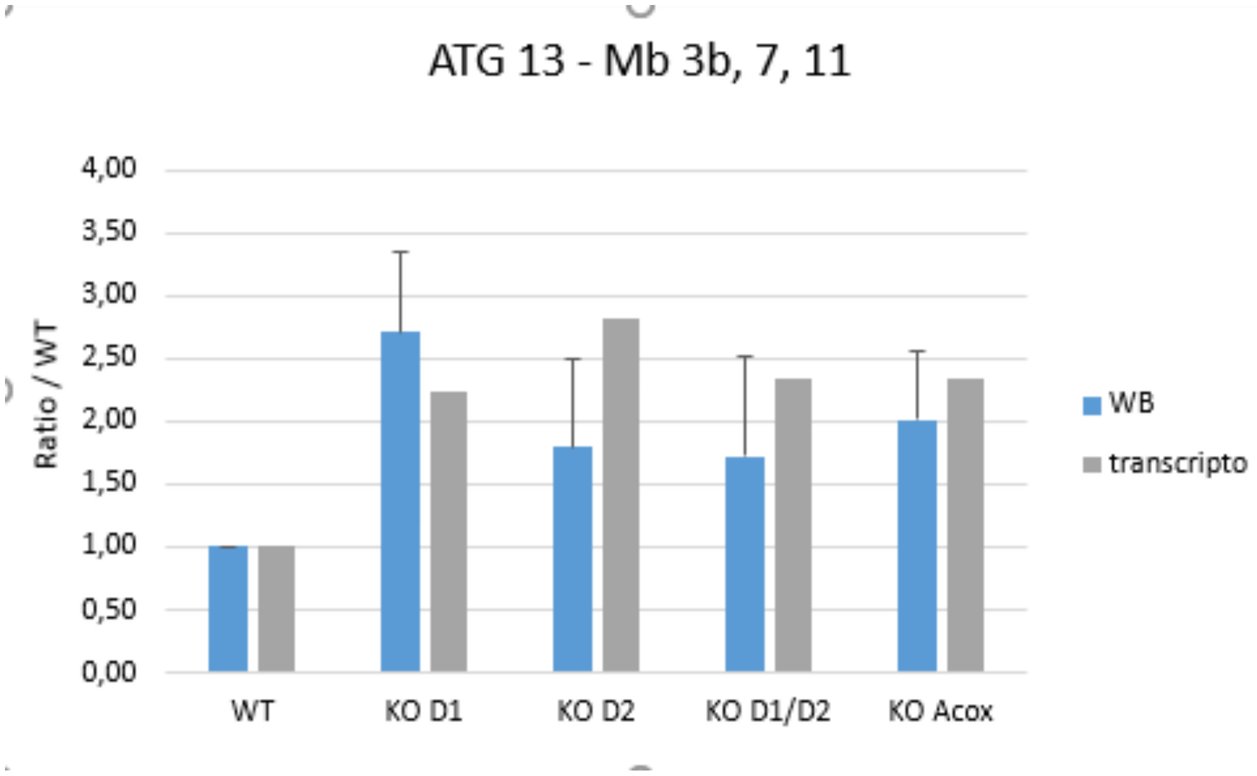

**LC3**

# Samples serie NS2
# WB Ali – Mb51

*Mb selected for the publication*

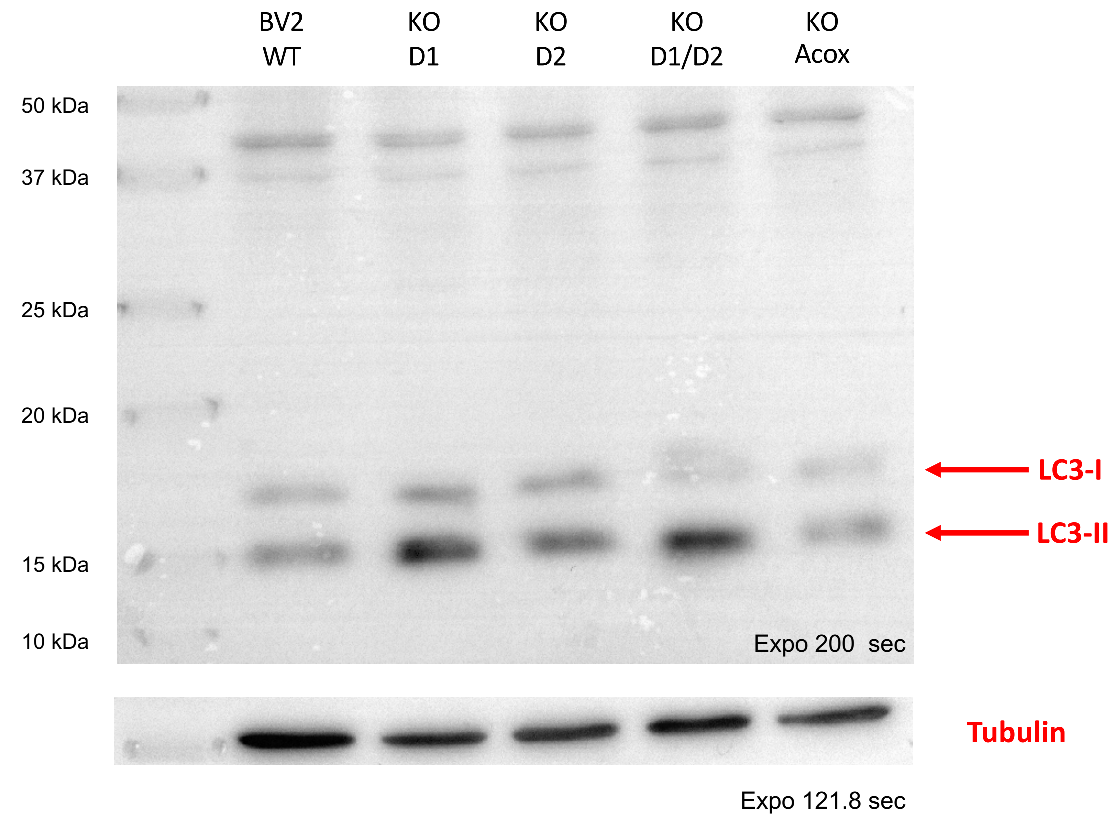


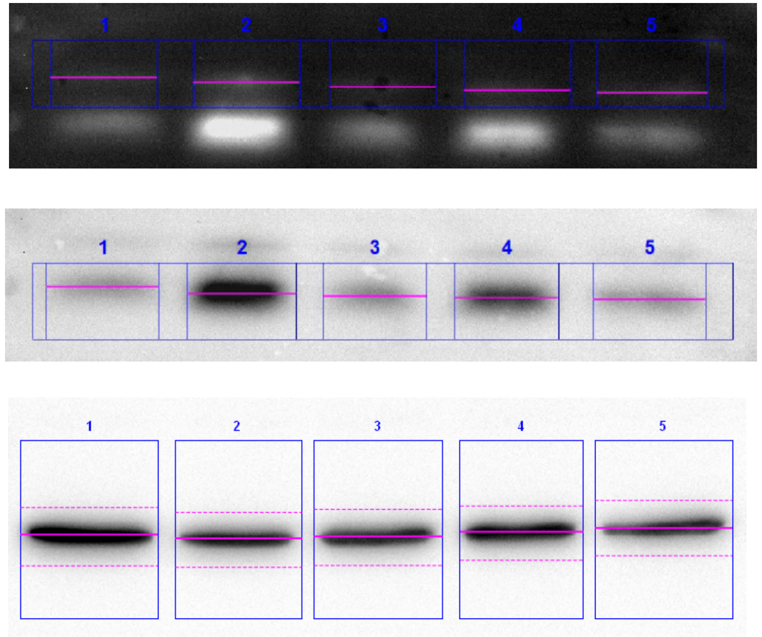


**LC3 (~ 17 kDa)**

**LC3-I**
Observed : 18 kDa

**LC3-II**
Observed : 16 kDa

Antibody Sigma # L8918

14% Gel
30 µg/load
PVDF Transfer

## Densitometric analysis

| Mb 51 | | LC3-I + LC3-II | Tubuline | (LC3-I + LC3-II )/ Tubuline | Ratio /WT |
|---|---|---|---|---|---|
| NS2 11/05/2021 | WT | 46 725 980 | 152 367 790 | 0.307 | 1.00 |
| | KO D1 | 143 834 524 | 95 172 165 | 1.511 | 4.93 |
| | KO D2 | 40 726 784 | 97 894 446 | 0.416 | 1.36 |
| | KO D1/D2 | 92 762 512 | 105 402 338 | 0.880 | 2.87 |
| | KO Acox | 51 728 405 | 92 031 246 | 0.562 | 1.83 |

| | LC3 -I | LC3 -II | Ratio LC3-II/LC3-I | Ratio /WT |
|---|---|---|---|---|
| WT | 5 699 200 | 41 026 780 | 7.199 | 1.00 |
| KO D1 | 15 962 624 | 127 871 900 | 8.011 | 1.11 |
| KO D2 | 5 542 528 | 35 184 256 | 6.348 | 0.88 |
| KO D1/D2 | 8 859 648 | 83 902 864 | 9.470 | 1.32 |
| KO Acox | 13 653 913 | 38 074 492 | 2.789 | 0.39 |

**LC3**

## Samples serie NS4
## WB Ali – Mb52

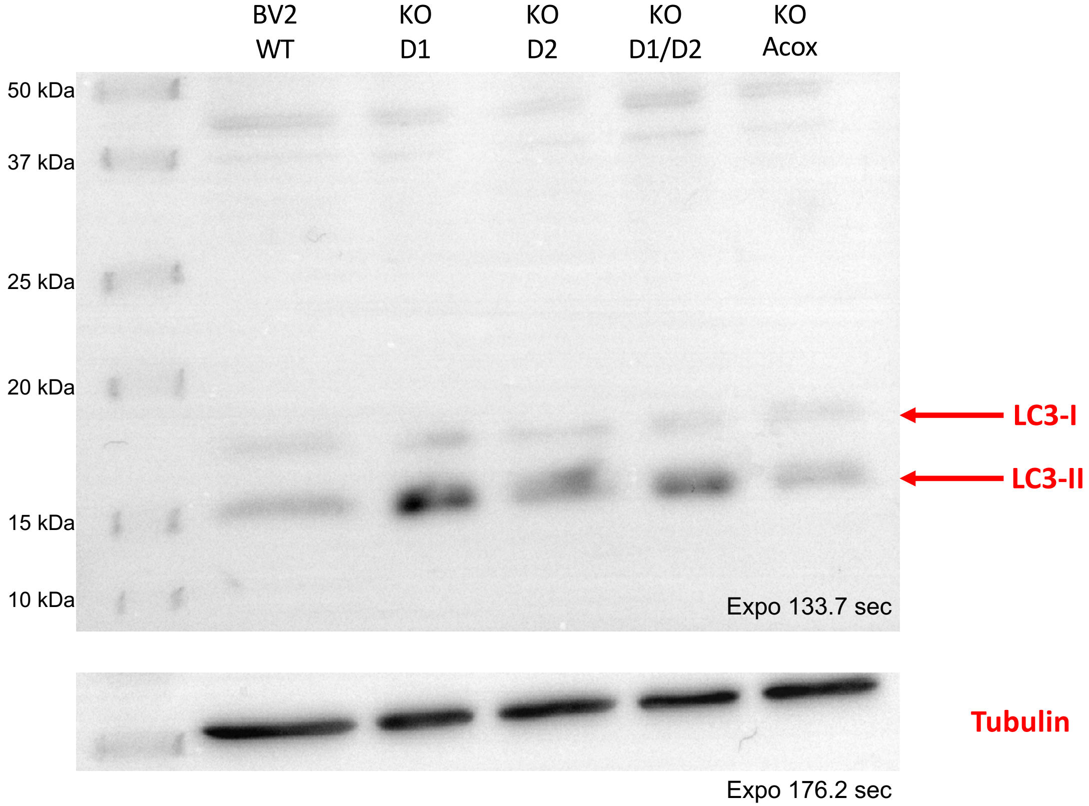


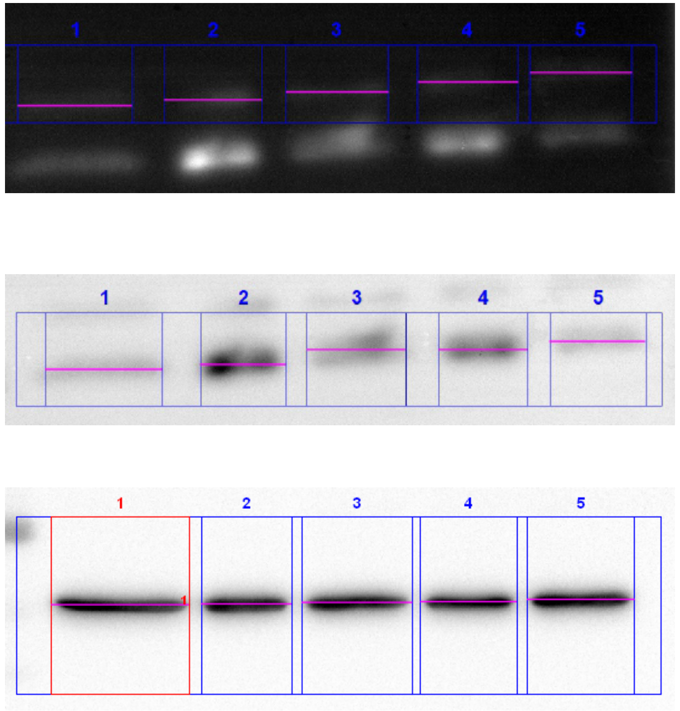


14% Gel
30 µg/load
PVDF Transfer

## Densitometric analysis

| Mb 52 | | LC3-I + LC3-II | Tubuline | (LC3-I + LC3-II )/ Tubuline | Ratio /WT |
|---|---|---|---|---|---|
| NS4 11/05/2021 | WT | 32 789 596 | 175 922 292 | 0.186 | 1.00 |
| | KO D1 | 72 688 120 | 116 533 560 | 0.624 | 3.35 |
| | KO D2 | 44 690 720 | 137 808 825 | 0.324 | 1.74 |
| | KO D1/D2 | 50 607 720 | 118 181 888 | 0.428 | 2.30 |
| | KO Acox | 22 585 433 | 142 336 051 | 0.159 | 0.85 |

| | LC3 -I | LC3 -II | Ratio LC3-II/LC3-I | Ratio /WT |
|---|---|---|---|---|
| WT | 10 712 856 | 22 076 740 | 2.061 | 1.00 |
| KO D1 | 12 670 448 | 60 017 672 | 4.737 | 2.30 |
| KO D2 | 8 431 280 | 36 259 440 | 4.301 | 2.09 |
| KO D1/D2 | 7 826 868 | 42 780 852 | 5.466 | 2.65 |
| KO Acox | 5 878 301 | 16 707 132 | 2.842 | 1.38 |

**LC3**

# Samples serie S1 (21/10/21)
# WB Catherine 5/12/2022 – Mb « LC3 1-2 »

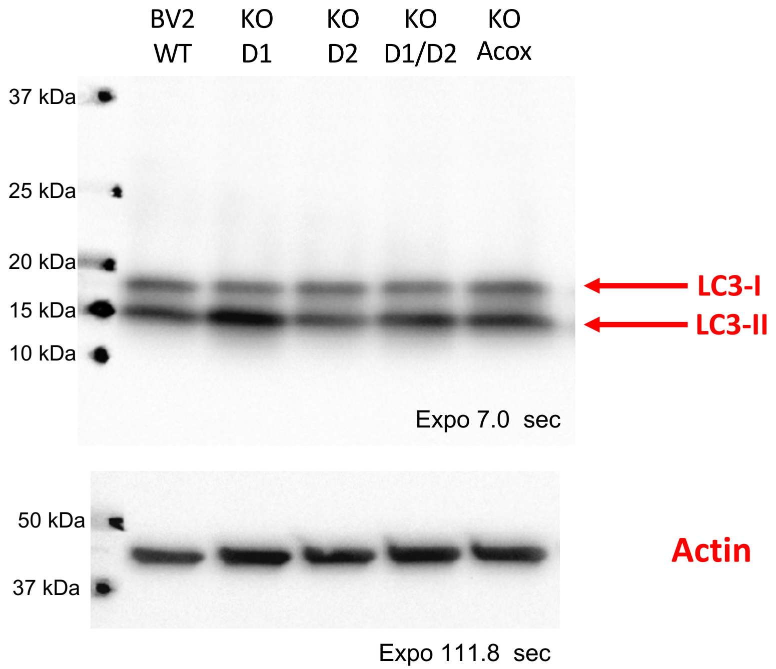


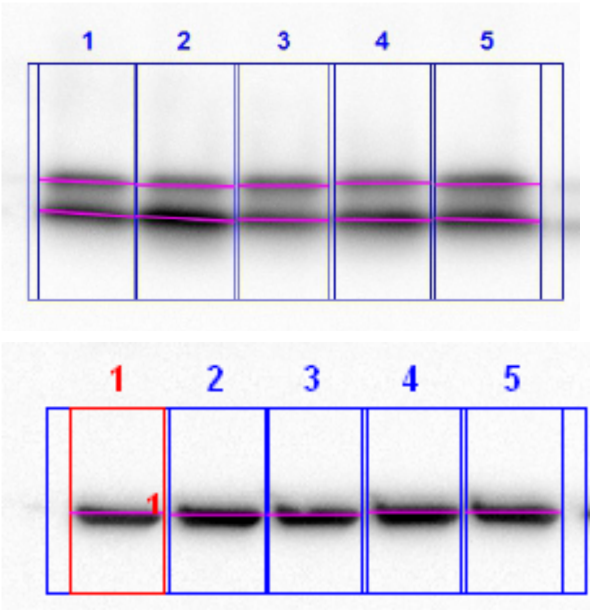


Gradient Gel 4-20%
30 µg/load
PVDF Transfer

## Densitometric analysis

| Mb LC3 1-2 | | LC3-I + LC3-II | Actine | (LC3-I + LC3-II )/ Actine | Ratio /WT |
|---|---|---|---|---|---|
| S1 - 21/10/2021 | WT | 73 583 400 | 41 139 459 | 1.789 | 1.00 |
| | KO D1 | 93 882 825 | 55 574 398 | 1.689 | 0.94 |
| | KO D2 | 73 298 610 | 52 239 960 | 1.403 | 0.78 |
| | KO D1/D2 | 82 665 346 | 56 879 253 | 1.453 | 0.81 |
| | KO Acox | 91 350 261 | 51 916 536 | 1.760 | 0.98 |

| | LC3 -I | LC3 -II | Ratio LC3-II/LC3-I | Ratio /WT |
|---|---|---|---|---|
| WT | 27 657 600 | 45 925 800 | 1.661 | 1.00 |
| KO D1 | 25 454 025 | 68 428 800 | 2.688 | 1.62 |
| KO D2 | 30 779 910 | 42 518 700 | 1.381 | 0.83 |
| KO D1/D2 | 27 985 499 | 54 679 847 | 1.954 | 1.18 |
| KO Acox | 35 410 608 | 55 939 653 | 1.580 | 0.95 |

LC3

# Samples serie S2 (27/10/21)
# WB Catherine 5/12/2022 – Mb « LC3 1-2 »

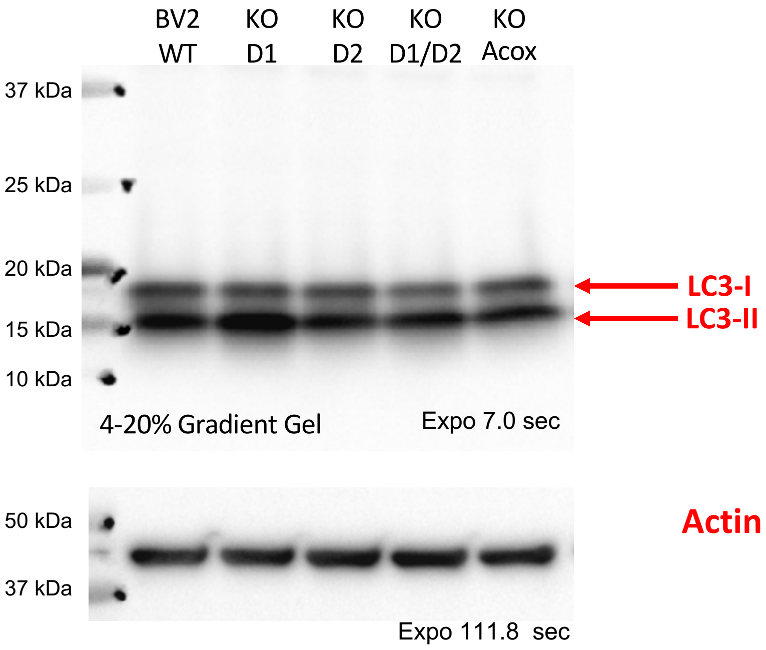


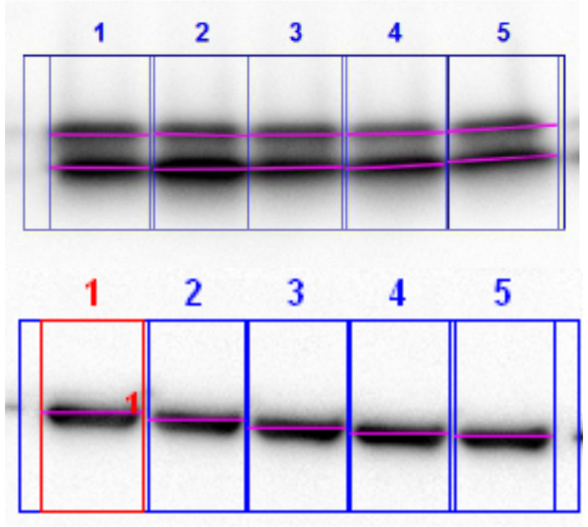


Gradient Gel 4-20%
30 µg/load
PVDF Transfer

## Densitometric analysis

| Mb LC3 1-2 | | LC3-I + LC3-II | Actine | (LC3-I + LC3-II )/ Actine | Ratio /WT |
|---|---|---|---|---|---|
| S2 - 27/10/2021 | WT | 113 913 492 | 57 472 575 | 1.982 | 1.00 |
| | KO D1 | 120 391 344 | 54 925 813 | 2.192 | 1.11 |
| | KO D2 | 100 975 536 | 59 356 800 | 1.701 | 0.86 |
| | KO D1/D2 | 101 131 300 | 60 448 392 | 1.673 | 0.84 |
| | KO Acox | 105 426 804 | 53 948 736 | 1.954 | 0.99 |

| | LC3 -I | LC3 -II | Ratio LC3-II/LC3-I | Ratio /WT |
|---|---|---|---|---|
| WT | 45 054 933 | 68 858 559 | 1.528 | 1.00 |
| KO D1 | 37 161 360 | 83 229 984 | 2.240 | 1.47 |
| KO D2 | 37 571 184 | 63 404 352 | 1.688 | 1.10 |
| KO D1/D2 | 33 081 888 | 68 049 412 | 2.057 | 1.35 |
| KO Acox | 38 312 652 | 67 114 152 | 1.752 | 1.15 |

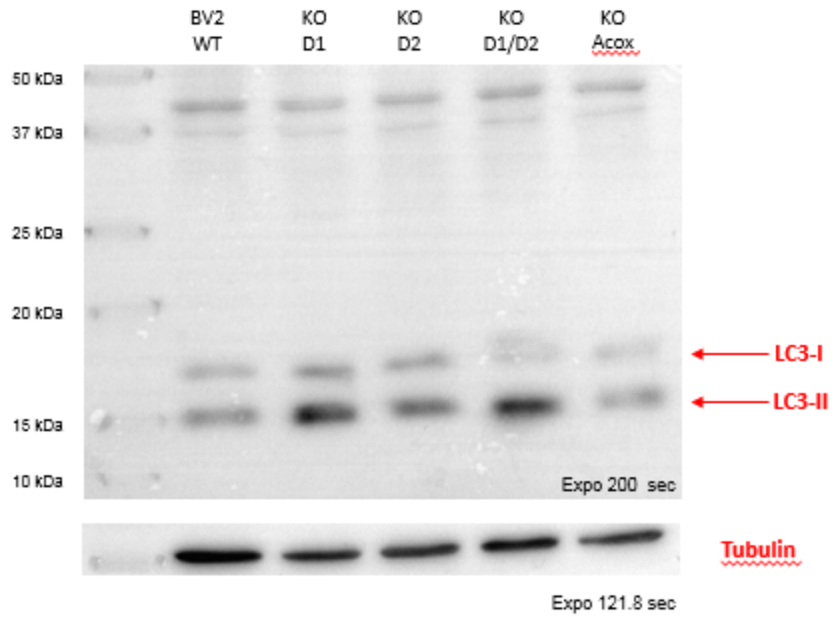


| | Transcriptomic *Map1lc3a* gene |
|---|---|
| | Ratio Mean |
| **WT** | **1.0** |
| **KO D1** | **1.8** |
| **KO D2** | **2.7** |
| **KO D1/D2** | **2.0** |
| **KO Acox** | **1.8** |

| | LC3 TOTAL Moyennes 4 blots | |
|---|---|---|
| | Ratio /WT | Ecrat-type |
| **WT** | 1.00 | |
| **KO D1** | 2.58 | 1.91 |
| **KO D2** | 1.18 | 0.45 |
| **KO D1/D2** | 1.71 | 1.04 |
| **KO Acox** | 1.16 | 0.45 |

| | RATIO LC3-II / LC3-I Moyennes 4 blots | |
|---|---|---|
| | Ratio /WT | Ecrat-type |
| **WT** | 1.00 | |
| **KO D1** | 1.62 | 0.50 |
| **KO D2** | 1.23 | 0.59 |
| **KO D1/D2** | 1.62 | 0.69 |
| **KO Acox** | 0.97 | 0.42 |

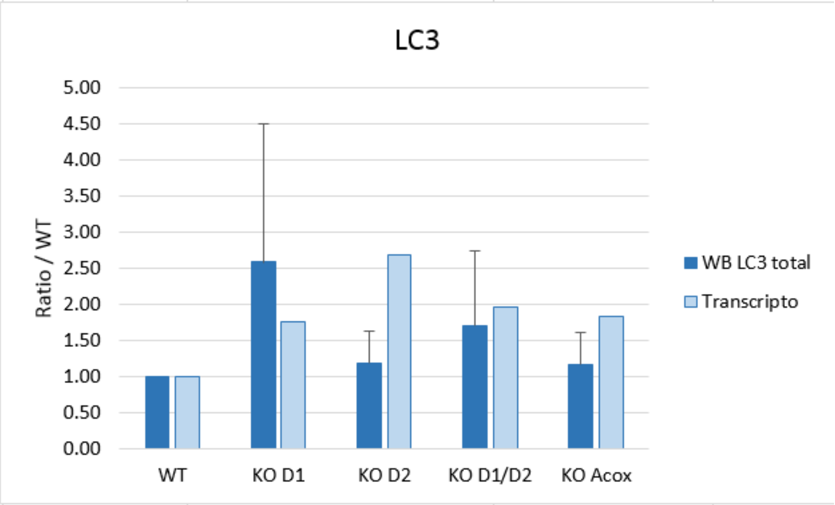


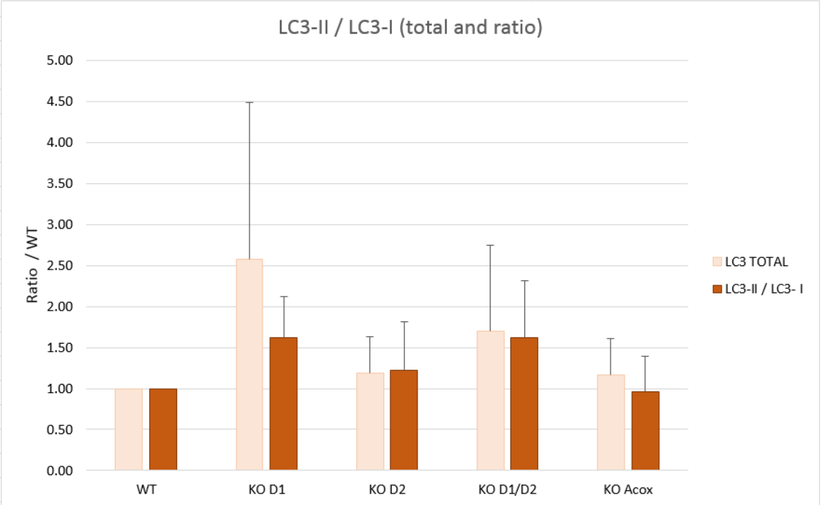


14% Gel
30 µg/load
PVDF Transfer

**p62**

# Samples serie S7
# WB Ali - Mb 32

*Mb selected for the publication*

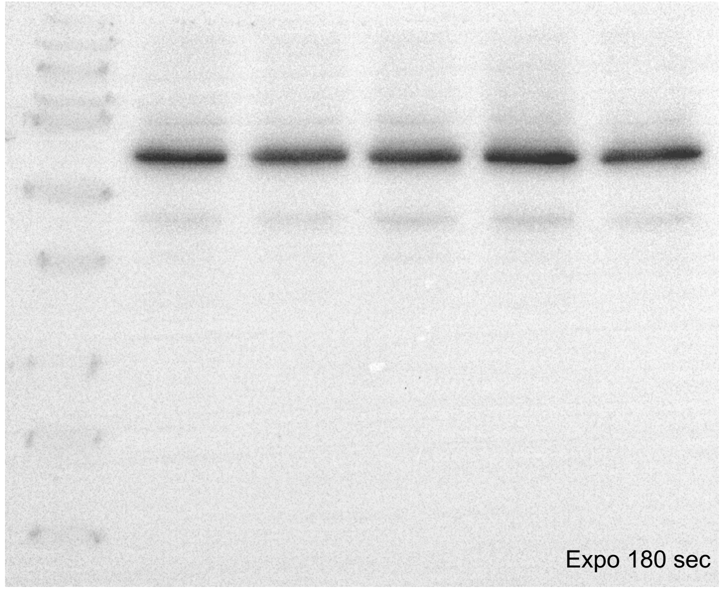


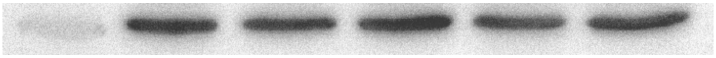

**p62**

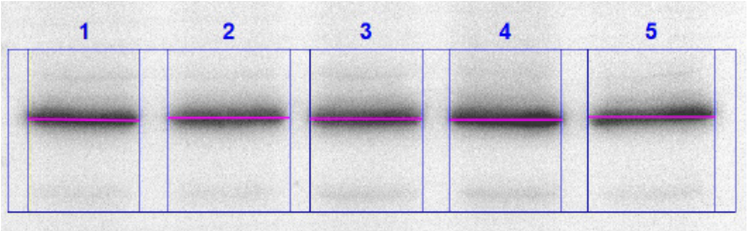


**Tubulin**

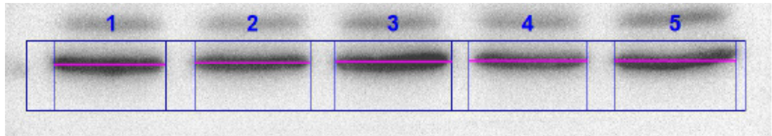


**P62**

MW : 62 kDa

Antibody : Abcam # ab56416

12% Gel
30 µg/load
PVDF Transfer

## Densitometric analysis

| S7 S5 | P62 | Tubuline | Ratio P62/tub | Ratio /WT |
|---|---|---|---|---|
| **WT** | 85,285,278 | 81,910,762 | 1.041 | **1.00** |
| **KO D1** | 85,947,377 | 79,164,336 | 1.086 | **1.04** |
| **KO D2** | 88,702,614 | 92,416,419 | 0.960 | **0.92** |
| **KO D1/D2** | 98,723,781 | 73,123,000 | 1.350 | **1.30** |
| **KO Acox** | 94,285,429 | 86,872,986 | 1.085 | **1.04** |

**p62**

# Samples serie NS 1
# WB Ali - Mb 38

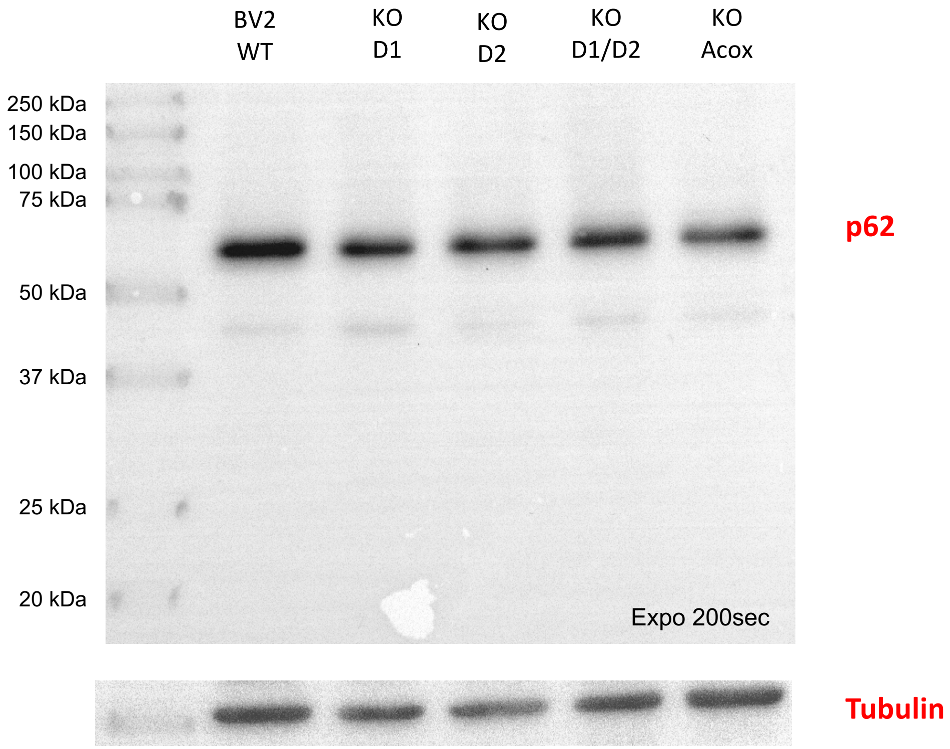


Expo 10.1 sec

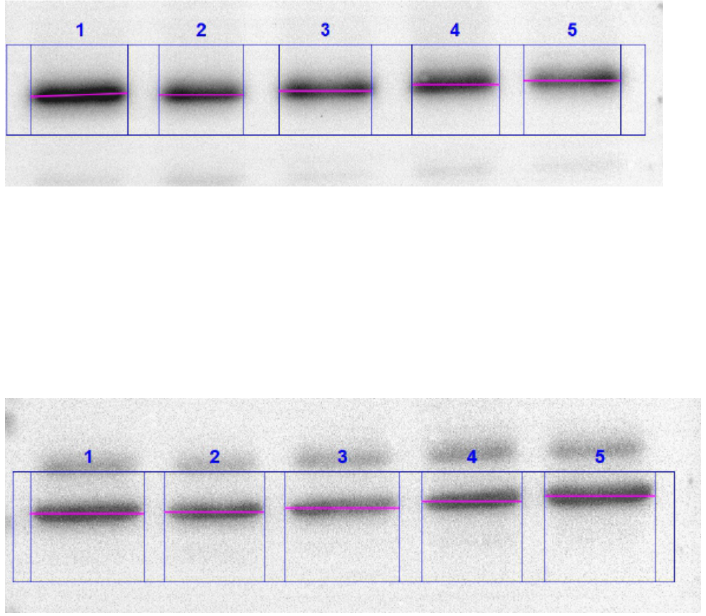


12% Gel
30 µg/load
PVDF Transfer

## Densitometric analysis

| NS1 - 01/05/2021 | P62 | Tubuline | Ratio P62/tub | Ratio /WT |
|---|---|---|---|---|
| **WT** | 122,025,796 | 95,539,588 | 1.277 | **1.00** |
| **KO D1** | 84,643,920 | 75,697,040 | 1.118 | **0.88** |
| **KO D2** | 91,211,994 | 71,266,108 | 1.280 | **1.00** |
| **KO D1/D2** | 84,968,280 | 76,387,720 | 1.112 | **0.87** |
| **KO Acox** | 74,999,912 | 94,395,466 | 0.795 | **0.62** |

**p62**

## Samples serie NS3
## WB Ali - Mb 54

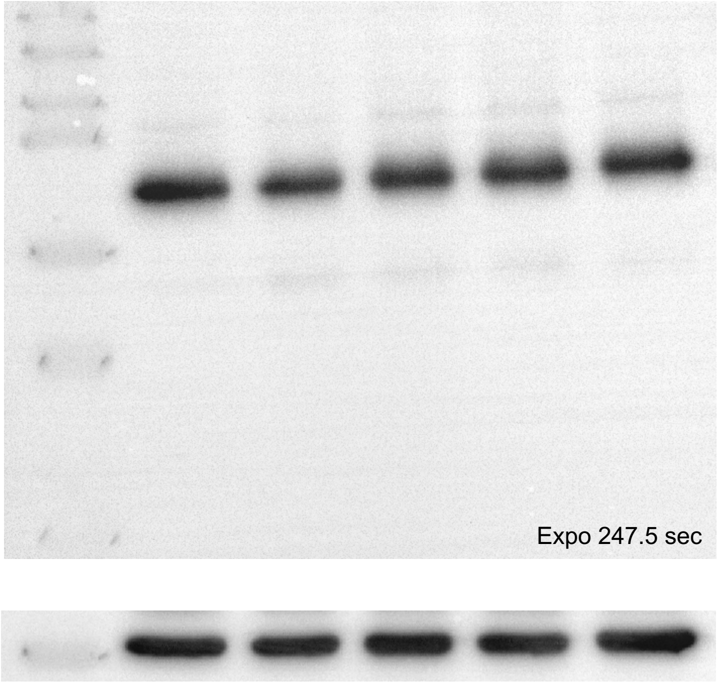


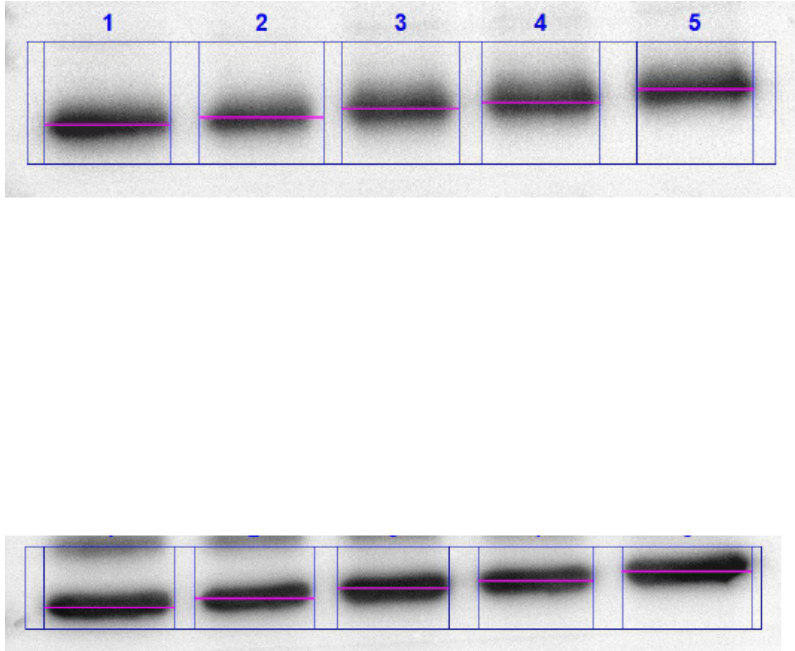


12% Gel
30 µg/load
PVDF Transfer

## Densitometric analysis

| NS3 - 18/05/2021 | P62 | Tubuline | Ratio P62/tub | Ratio /WT |
|---|---|---|---|---|
| **WT** | 125,331,264 | 104,406,874 | 1.200 | **1.00** |
| **KO D1** | 102,490,980 | 96,488,050 | 1.062 | **0.88** |
| **KO D2** | 118,627,864 | 118,745,894 | 0.999 | **0.83** |
| **KO D1/D2** | 125,831,277 | 118,526,814 | 1.062 | **0.88** |
| **KO Acox** | 124,924,040 | 147,229,434 | 0.848 | **0.71** |

# p62

| | Transcriptomique<br>Moyenne Ratio |
|---|---|
| **WT** | 1 |
| **KO D1** | 1.55 |
| **KO D2** | 1.28 |
| **KO D1/D2** | 1.79 |
| **KO Acox** | 1.22 |

| | Toutes les Mb | |
|---|---|---|
| | **Moyenne Ratio** | **Ecart-type Ratio** |
| **WT** | 1 | 0 |
| **KO D1** | 0.93 | 0.09 |
| **KO D2** | 0.92 | 0.08 |
| **KO D1/D2** | 1.02 | 0.24 |
| **KO Acox** | 0.79 | 0.22 |

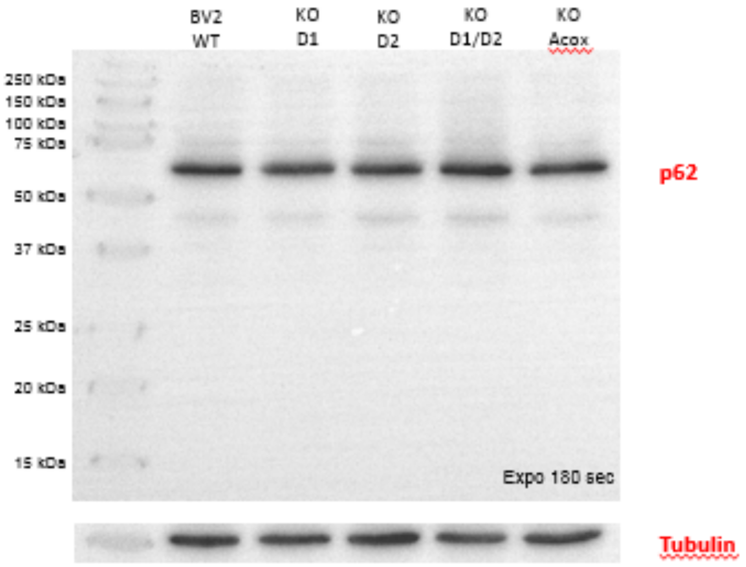


12% Gel
30 µg/load
PVDF Transfer

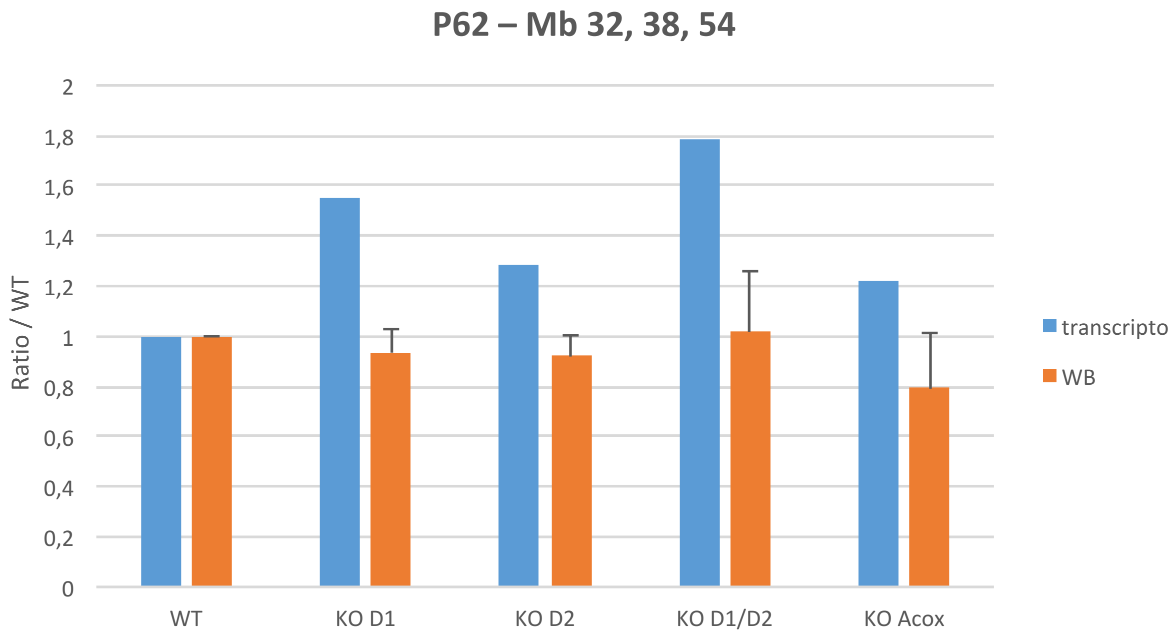

# Lamtor4

**Lamtor 4**
MW : 11 kDa
Observed : ~ 12 kDa

Primary antibody : Cell Signaling # 12284

Gradient Gel 4-20%
PVDF Transfer
30 µg/load

| | Transcriptomic Ratio Mean |
|---|---|
| WT | 1.0000 |
| KO D1 | 0.0008 |
| KO D2 | 0.0003 |
| KO D1/D2 | 0.0003 |
| KO Acox | 0.0002 |

## Samples serie S1 (21/10/21)
## WB Catherine 5/12/2022 – Mb « L 1-2 »

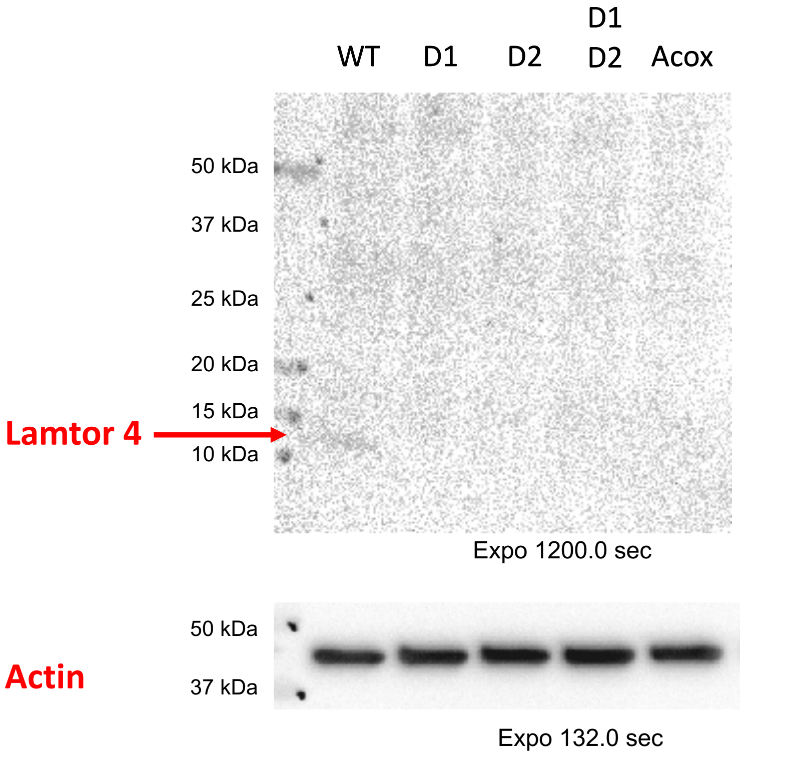


## Samples serie S2 (27/10/21)
## WB Catherine 5/12/2022 – Mb « L 1-2 »

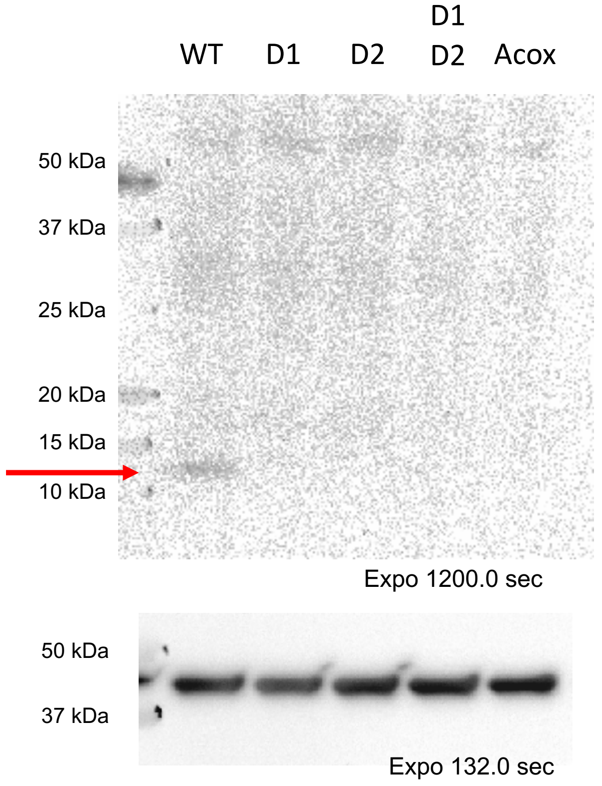


## Samples serie S3 (28/10/21)
## WB Catherine 5/12/2022 – Mb « L 3 »

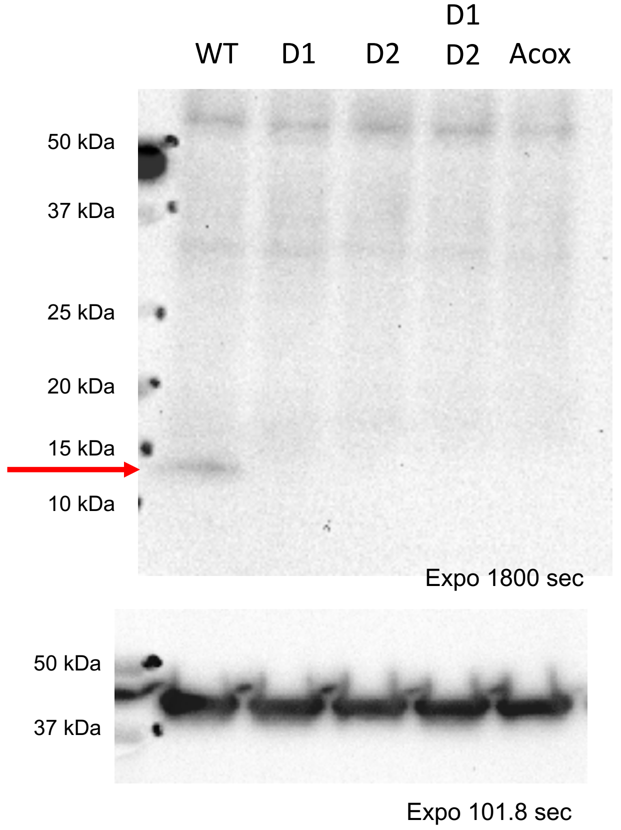


*Mb selected for the publication*

# mTor

## Samples serie S1 (21/10/2021)
## WB Ali (16/11/2022)

*Mbs selected for the publication*

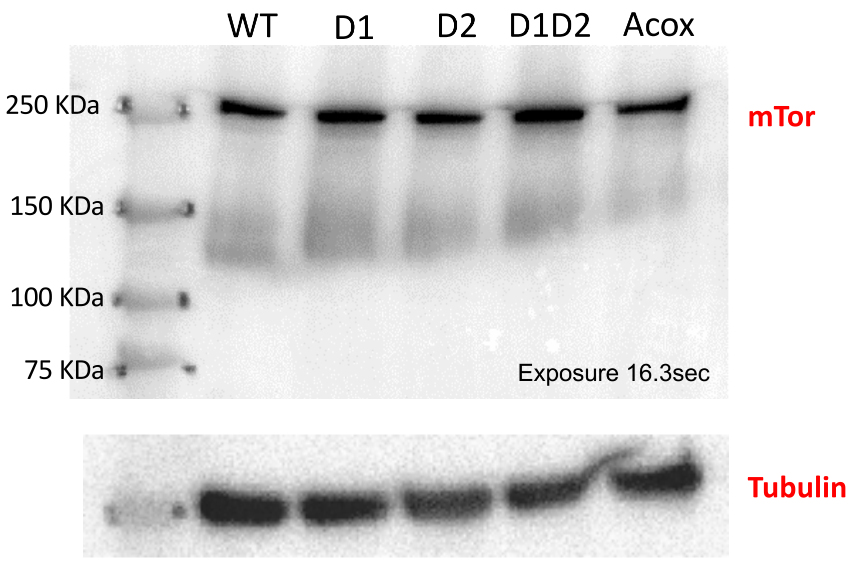


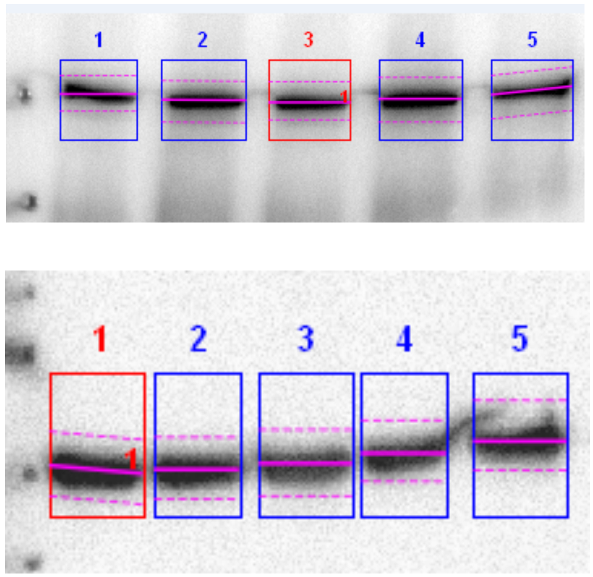


**mTor**

~ 290 kDa

Antibodies :

**mTor** : Cell Signaling # 2983

**P-mTor (Ser2448)** : Cell Signaling # 5536

Gradient Gel 4-20%
PVDF Transfer
30 µg/load

# p-mTor

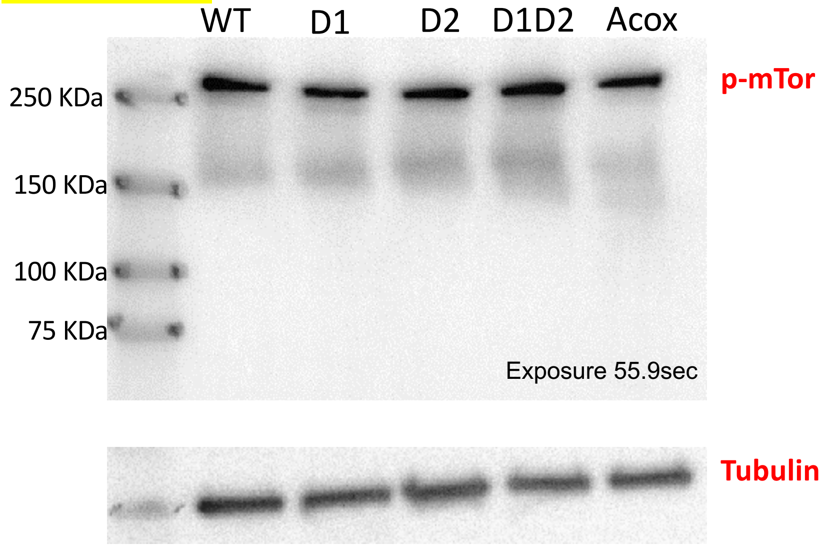


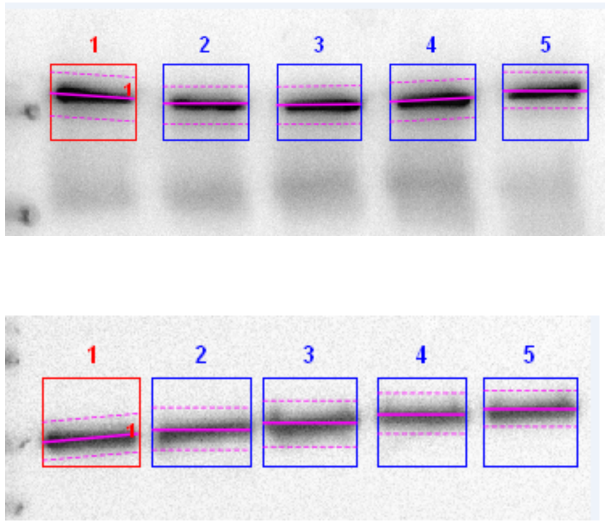


## Densitometric analysis

| S1 | n° bande | mTOR | Tubulin | Ratio mTOR/tub | Ratio PmTOR/mTOR |
|---|---|---|---|---|---|
| WT | 1 | 26 522 600 | 26 321 016 | 1.01 | |
| WT P-mTOR | 1 | 32 281 708 | 29 229 970 | 1.10 | 1.10 |
| KO D1 | 2 | 35 149 331 | 22 784 664 | 1.54 | |
| KO D1 P-mTOR | 2 | 29 467 256 | 27 714 600 | 1.06 | 0.69 |
| KO D2 | 3 | 30 872 118 | 22 333 276 | 1.38 | |
| KO D2 P-mTOR | 3 | 32 923 084 | 31 120 035 | 1.06 | 0.77 |
| KO D1/D2 | 4 | 37 542 260 | 21 060 816 | 1.78 | |
| KO D1/D2 P-mTOR | 4 | 33 195 424 | 24 157 120 | 1.37 | 0.77 |
| KO Acox | 5 | 29 828 948 | 27 495 396 | 1.08 | |
| KO Acox P-mTOR | 5 | 24 980 956 | 24 241 184 | 1.03 | 0.95 |

**mTor**

# Samples serie S2 (27/10/2021)
# WB Ali (16/11/2022)

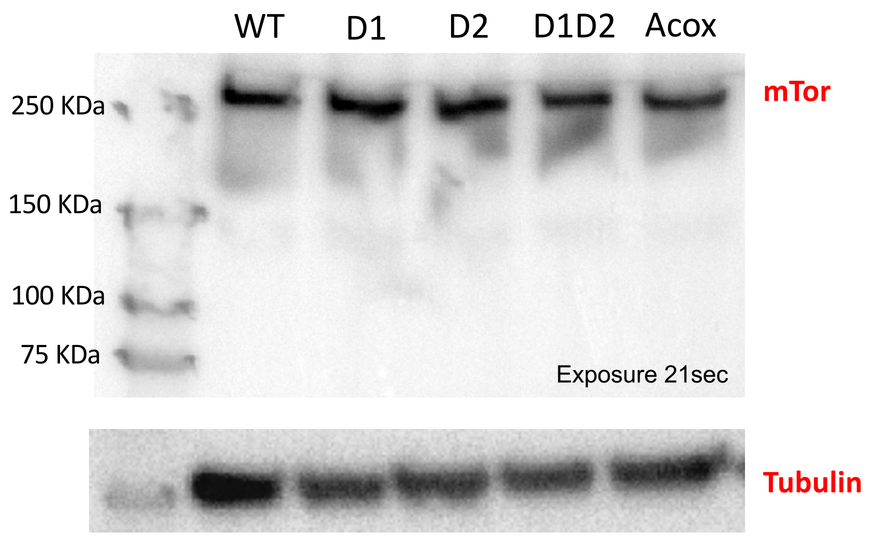


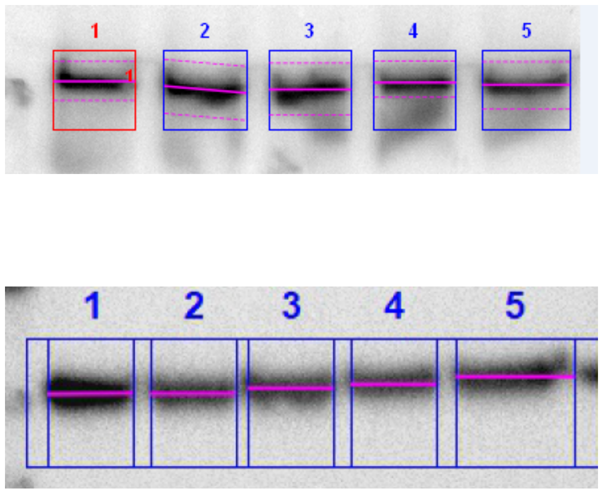


Gradient Gel 4-20%
PVDF Transfer
30 µg/load

## Densitometric analysis

| S2 | n° bande | mTOR | Tubulin | Ratio mTOR/tub | Ratio PmTOR/mTOR |
|---|---|---|---|---|---|
| WT | 1 | 28 306 493 | 26 145 196 | 1.083 | |
| WT P-mTOR | 1 | 32 626 275 | 25 303 159 | 1.289 | 1.19 |
| KO D1 | 2 | 45 658 467 | 19 210 752 | 2.377 | |
| KO D1 P-mTOR | 2 | 32 777 628 | 20 017 723 | 1.637 | 0.69 |
| KO D2 | 3 | 45 811 188 | 20 701 780 | 2.213 | |
| KO D2 P-mTOR | 3 | 34 286 775 | 20 888 010 | 1.641 | 0.74 |
| KO D1/D2 | 4 | 29 846 767 | 17 638 280 | 1.692 | |
| KO D1/D2 P-mTOR | 4 | 26 070 225 | 19 424 295 | 1.342 | 0.79 |
| KO Acox | 5 | 34 579 012 | 25 877 481 | 1.336 | |
| KO Acox P-mTOR | 5 | 14 908 974 | 19 106 835 | 0.780 | 0.58 |

**p-mTor**

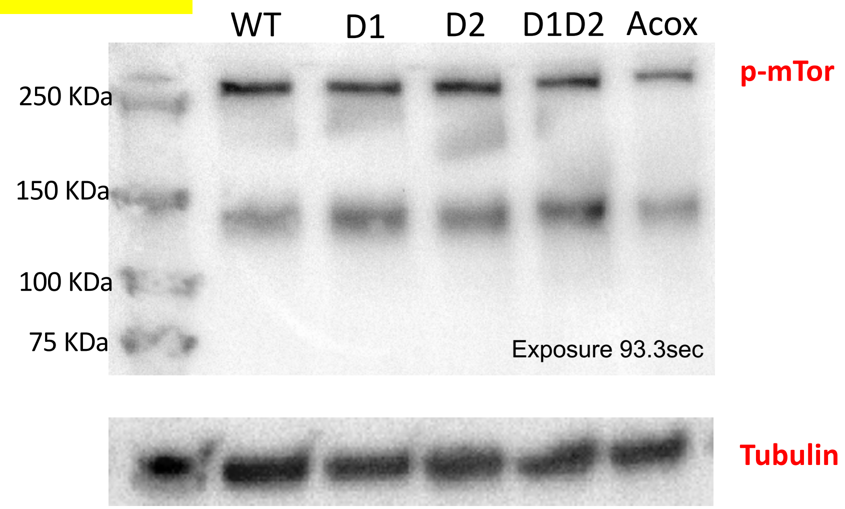


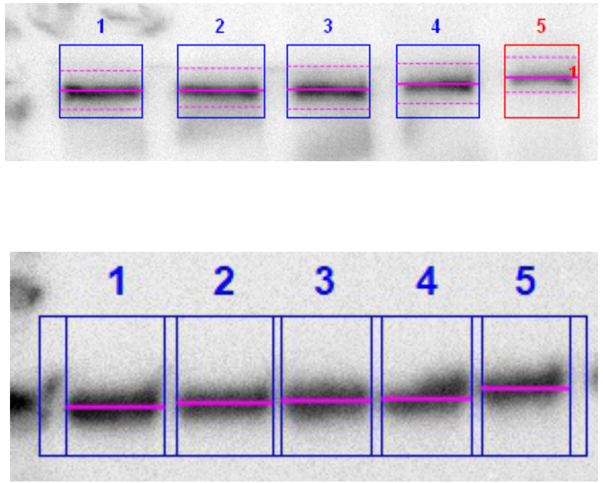

mTor

# Samples serie S3 (28/10/2021)
# WB Ali (16/11/2022)

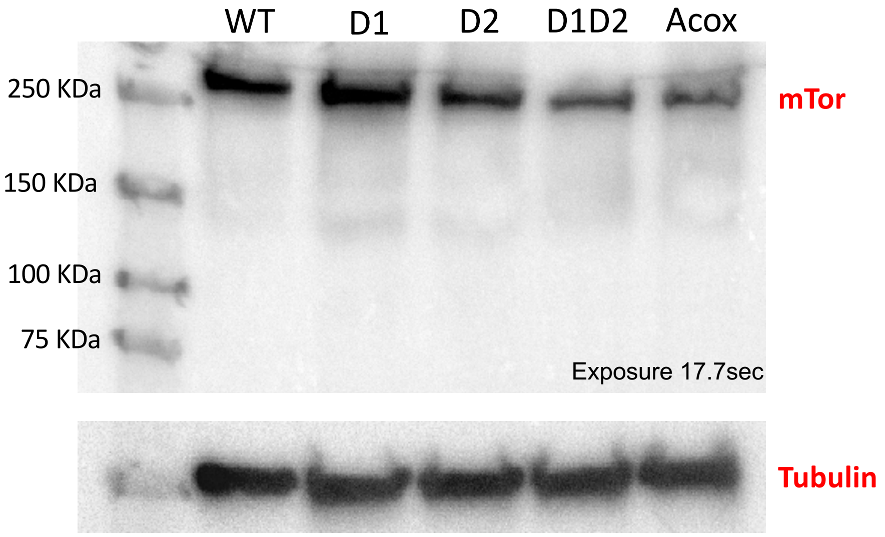


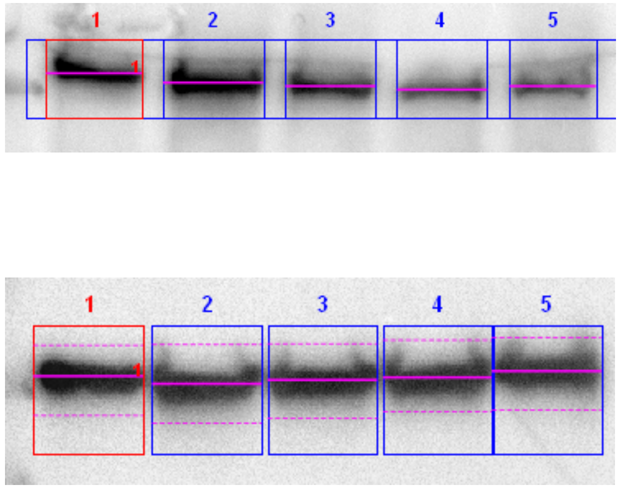


Gradient Gel 4-20%
PVDF Transfer
30 µg/load

p-mTor

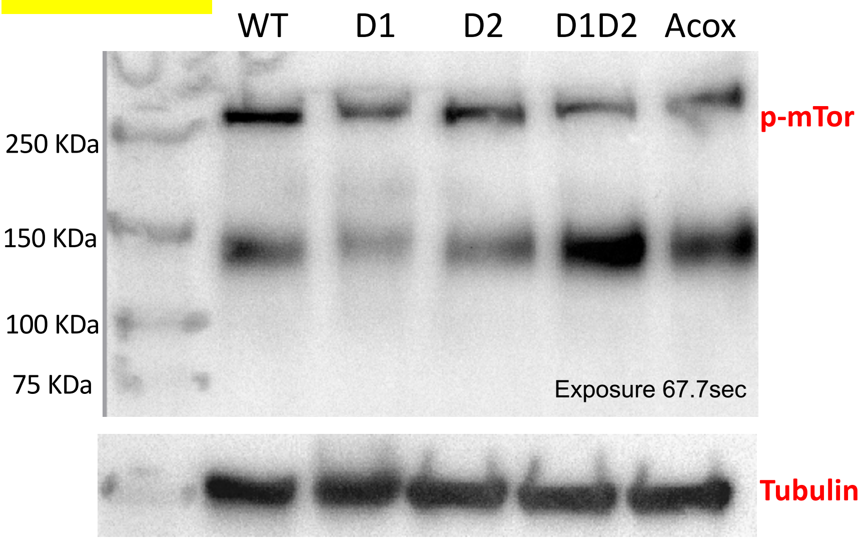


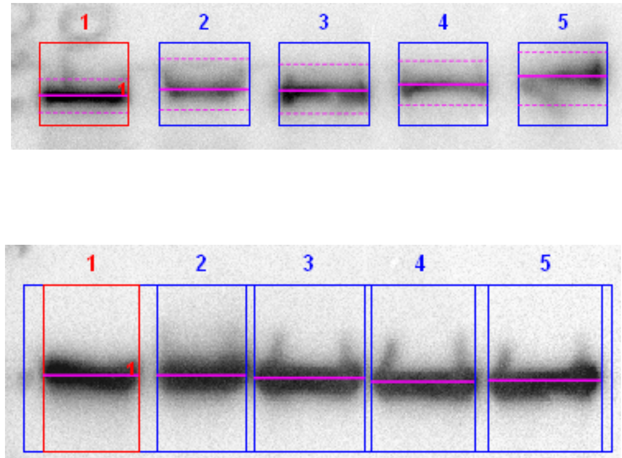


## Densitometric analysis

| S3 | n° bande | mTOR | Tubulin | Ratio mTOR/tub | Ratio PmTOR/mTOR |
|---|---|---|---|---|---|
| WT | 1 | 52 808 574 | 78 022 468 | 0.677 | |
| WT P-mTOR | 1 | 32 808 344 | 72 250 725 | 0.454 | 0.67 |
| KO D1 | 2 | 59 506 754 | 77 282 094 | 0.770 | |
| KO D1 P-mTOR | 2 | 30 206 800 | 75 394 340 | 0.401 | 0.52 |
| KO D2 | 3 | 40 888 540 | 78 039 324 | 0.524 | |
| KO D2 P-mTOR | 3 | 38 613 866 | 79 554 018 | 0.485 | 0.93 |
| KO D1/D2 | 4 | 35 917 959 | 76 111 634 | 0.472 | |
| KO D1/D2 P-mTOR | 4 | 26 603 074 | 70 439 544 | 0.378 | 0.80 |
| KO Acox | 5 | 26 849 864 | 69 539 170 | 0.386 | |
| KO Acox P-mTOR | 5 | 29 212 832 | 79 409 616 | 0.368 | 0.95 |

| | mTor Transcriptomic |
|---|---|
| | Ratio Mean |
| WT | 1.0 |
| KO D1 | 1.0 |
| KO D2 | 0.8 |
| KO D1/D2 | 1.0 |
| KO Acox | 0.8 |

| WB mTor (3 Mb) | | |
|---|---|---|
| | Moyenne Ratio | Ecart-type Ratio |
| WT | 1 | 0 |
| KO D1 | 1.62 | 0.53 |
| KO D2 | 1.40 | 0.64 |
| KO D1/D2 | 1.34 | 0.57 |
| KO Acox | 0.96 | 0.35 |

| WB p-mTor (3 Mb) | | |
|---|---|---|
| | Moyenne Ratio | Ecart-type Ratio |
| WT | 1 | 0 |
| KO D1 | 1.04 | 0.20 |
| KO D2 | 1.10 | 0.16 |
| KO D1/D2 | 1.04 | 0.21 |
| KO Acox | 0.78 | 0.17 |

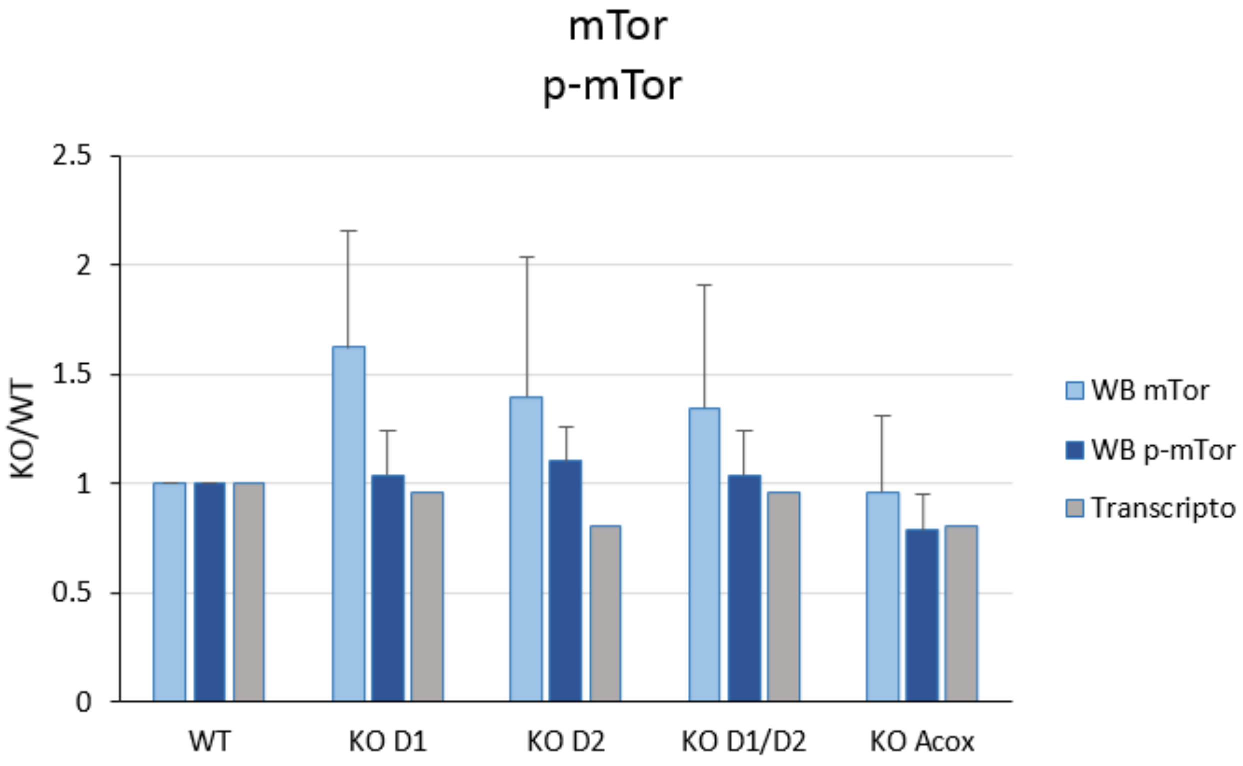


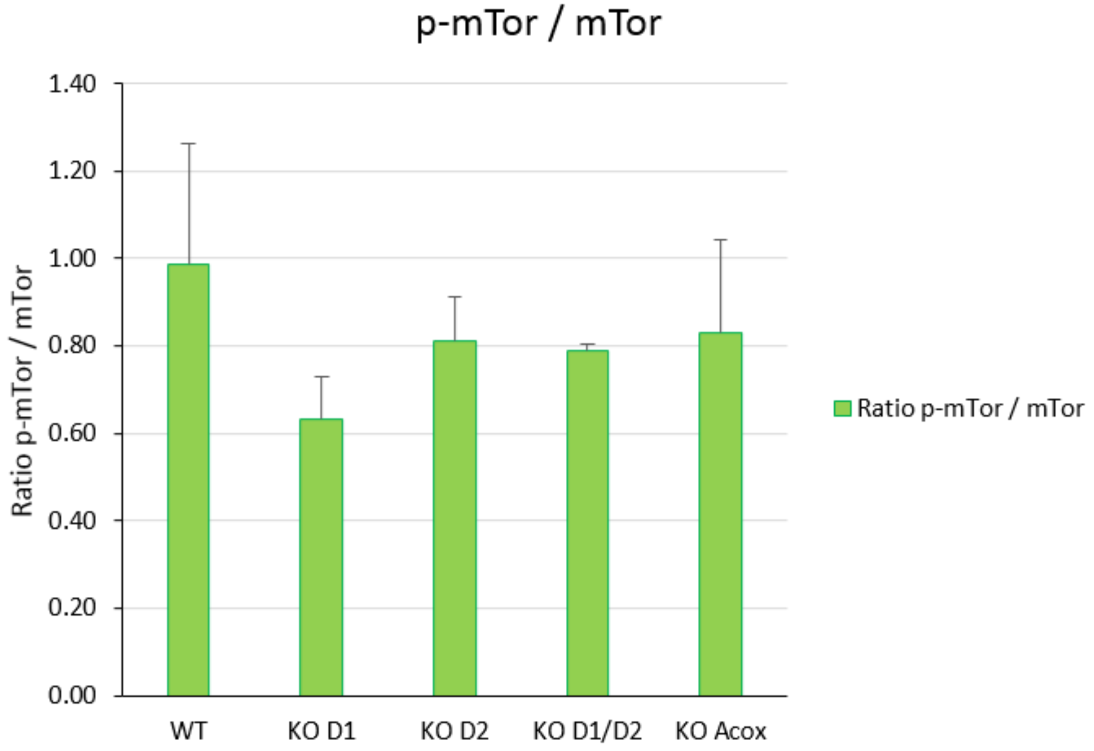

# Ulk1

# Samples serie S1 (21/10/2021)
# WB Ali

*Mbs selected for the publication*

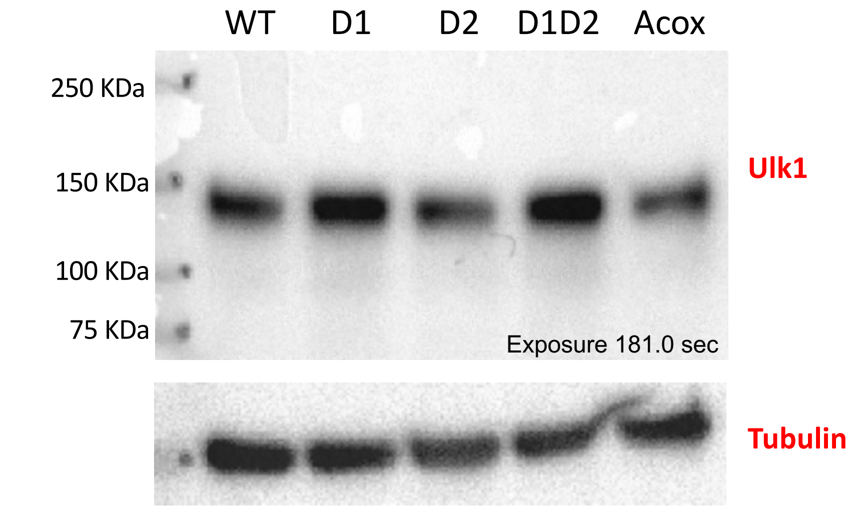


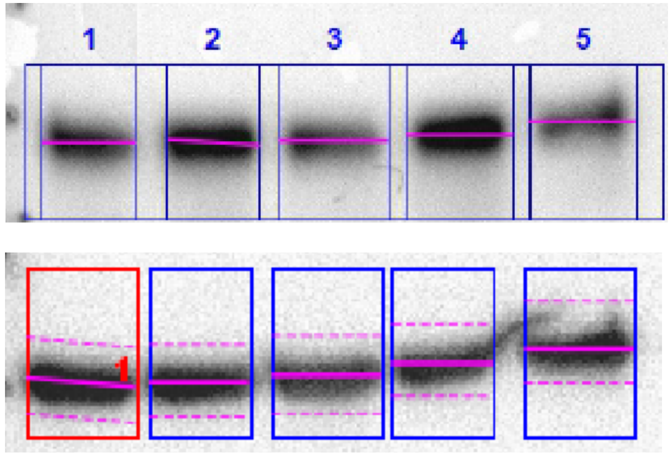


**Ulk1**

112 kDa (observed 150 kDa)

Antibodies :

**Ulk1** : Cell Signaling # 8054
**P-Ulk1(Ser757)**: Cell Signaling # 14202

Gradient Gel 4-20%
PVDF Transfer
30 µg/load

# p-Ulk1

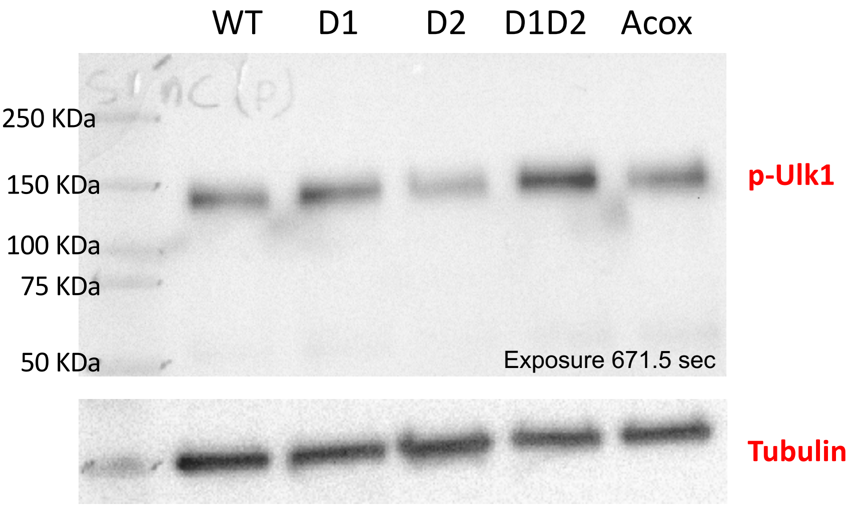


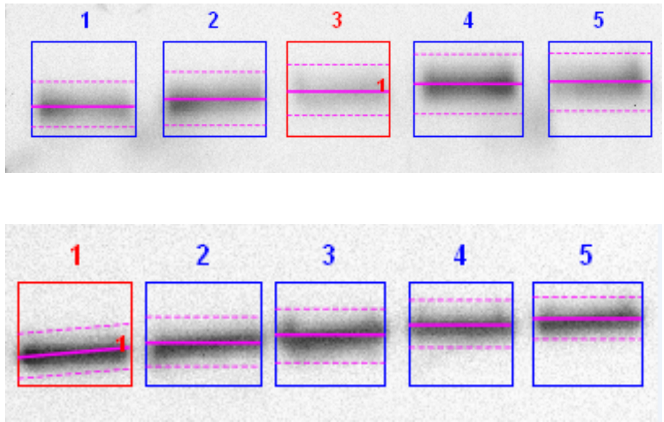


## Densitometric analysis

| S1 | n° bande | ULK1 | Tubulin | Ratio ULK1/tub | Ratio PULK1/ULK1 |
|---|---|---|---|---|---|
| WT | 1 | 45 153 054 | 26 321 016 | 1.715 | |
| WT P-ULK1 | 1 | 26 764 800 | 29 229 970 | 0.916 | 0.53 |
| KO D1 | 2 | 56 146 840 | 22 784 664 | 2.464 | |
| KO D1 P-ULK1 | 2 | 33 723 864 | 27 714 600 | 1.217 | 0.49 |
| KO D2 | 3 | 45 940 104 | 22 333 276 | 2.057 | |
| KO D2 P-ULK1 | 3 | 19 745 370 | 31 120 035 | 0.634 | 0.31 |
| KO D1/D2 | 4 | 60 619 729 | 21 060 816 | 2.878 | |
| KO D1/D2 P-ULK1 | 4 | 50 207 918 | 24 157 120 | 2.078 | 0.72 |
| KO Acox | 5 | 45 408 230 | 27 495 396 | 1.651 | |
| KO Acox P-ULK1 | 5 | 34 554 843 | 24 241 184 | 1.425 | 0.86 |

# Ulk1

# Samples serie S2 (27/10/2021)
# WB Ali

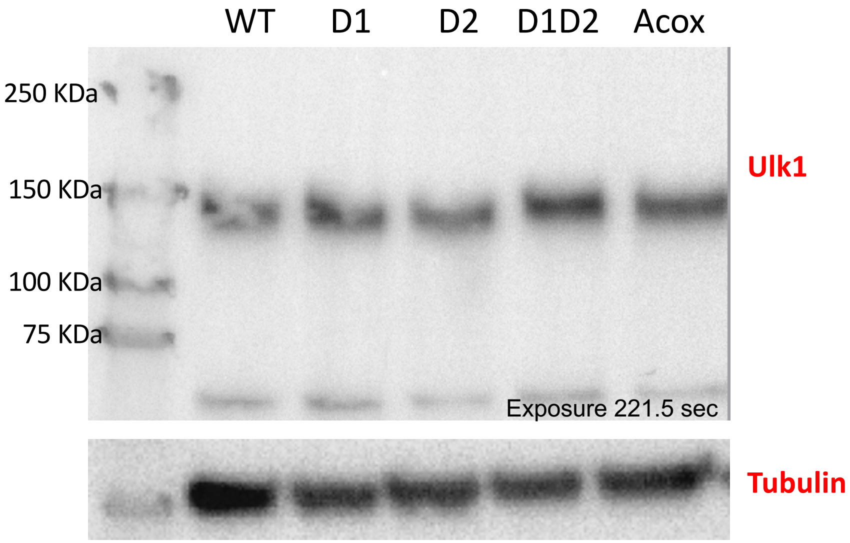


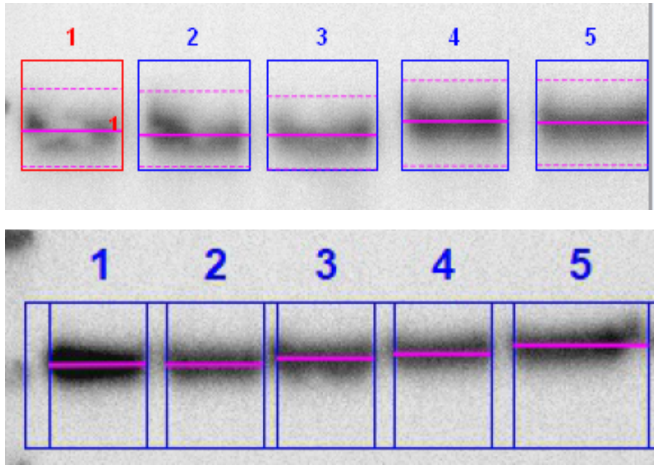


Gradient Gel 4-20%
PVDF Transfer
30 µg/load

# p-Ulk1

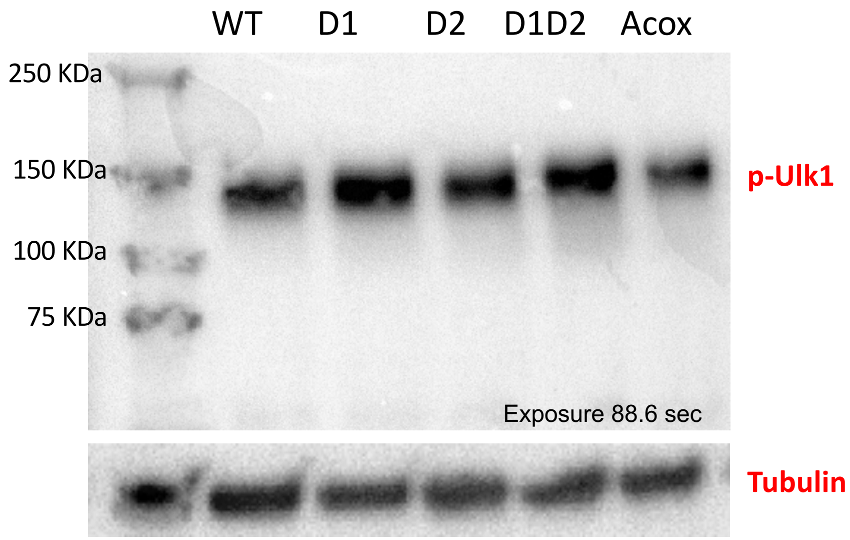


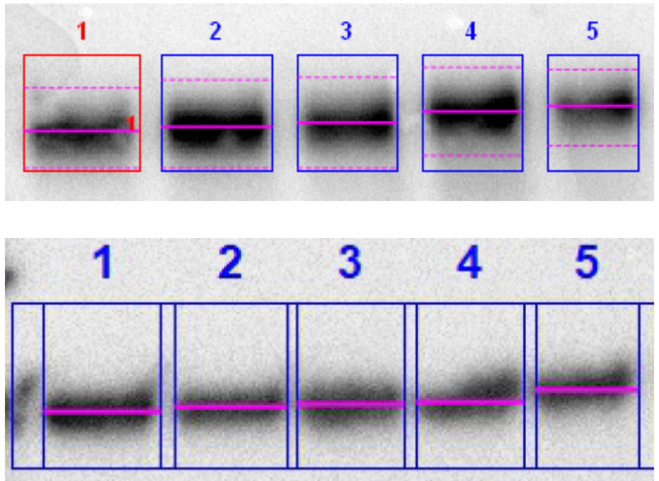


## Densitometric analysis

| S2 | n° bande | ULK1 | Tubulin | Ratio ULK1/tub | Ratio PULK1/ULK1 |
|---|---|---|---|---|---|
| WT | 1 | 33 594 600 | 26 145 196 | 1.285 | |
| WT P-ULK1 | 1 | 61 911 066 | 25 303 159 | 2.447 | 1.90 |
| KO D1 | 2 | 41 709 006 | 19 210 752 | 2.171 | |
| KO D1 P-ULK1 | 2 | 85 268 334 | 20 017 723 | 4.260 | 1.96 |
| KO D2 | 3 | 35 942 240 | 20 701 780 | 1.736 | |
| KO D2 P-ULK1 | 3 | 66 036 532 | 20 888 010 | 3.161 | 1.82 |
| KO D1/D2 | 4 | 47 651 527 | 17 638 280 | 2.702 | |
| KO D1/D2 P-ULK1 | 4 | 72 295 608 | 19 424 295 | 3.722 | 1.38 |
| KO Acox | 5 | 46 691 802 | 25 877 481 | 1.804 | |
| KO Acox P-ULK1 | 5 | 43 691 008 | 19 106 835 | 2.287 | 1.27 |

# Ulk1

# Samples serie S3 (28/10/2021)
# WB Ali

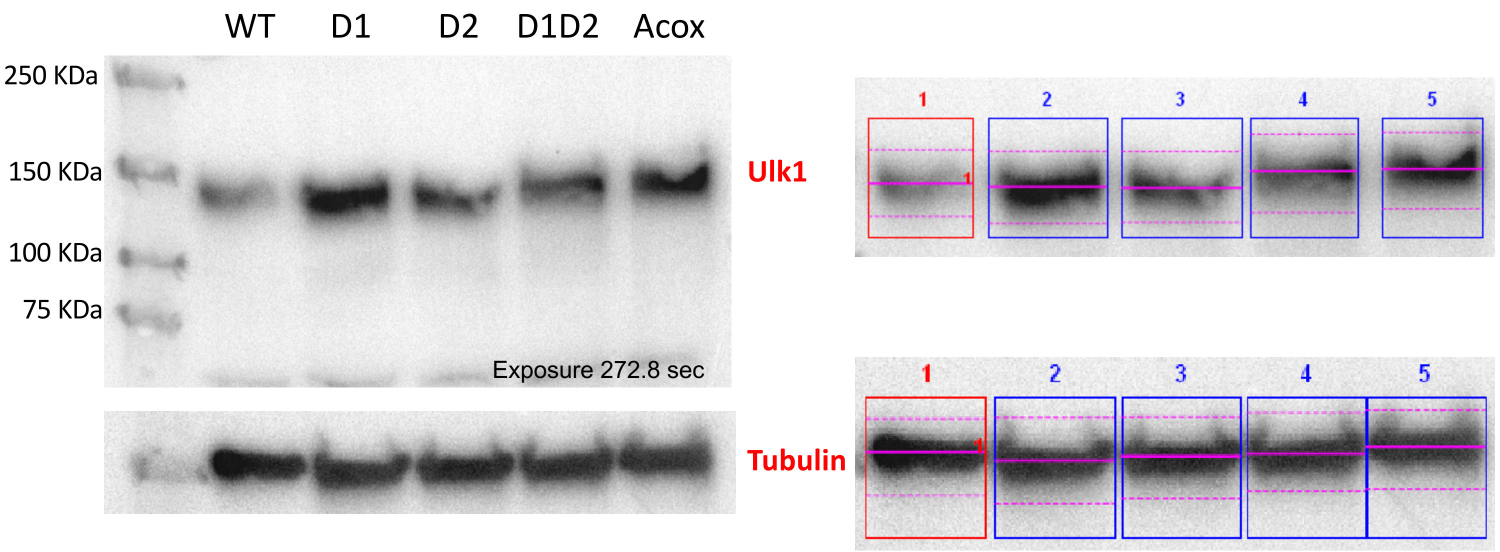


Gradient Gel 4-20%
PVDF Transfer
30 µg/load

# p-Ulk1

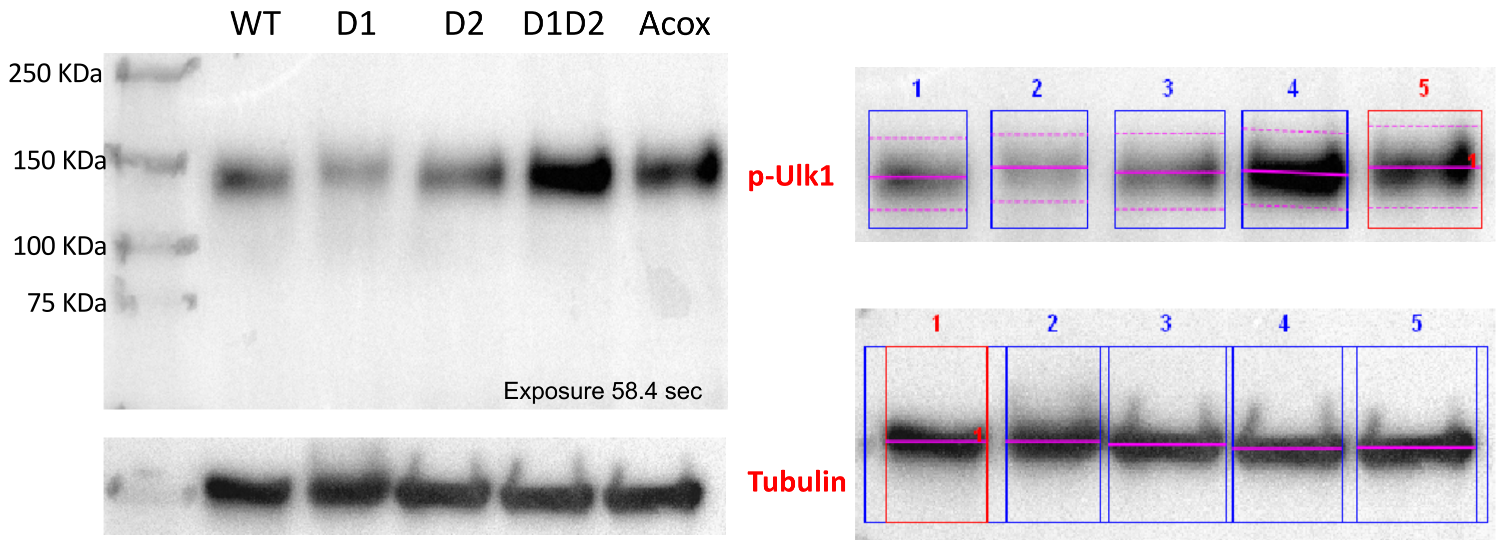


## Densitometric analysis

| S3 | n° bande | ULK1 | Tubulin | Ratio ULK1/tub | Ratio PULK1/ULK1 |
|---|---|---|---|---|---|
| WT | 1 | 48 629 114 | 78 022 468 | 0.623 | |
| WT P-ULK1 | 1 | 35 686 853 | 72 250 725 | 0.494 | 0.79 |
| KO D1 | 2 | 104 829 866 | 77 282 094 | 1.356 | |
| KO D1 P-ULK1 | 2 | 21 495 142 | 75 394 340 | 0.285 | 0.21 |
| KO D2 | 3 | 74 208 726 | 78 039 324 | 0.951 | |
| KO D2 P-ULK1 | 3 | 45 541 104 | 79 554 018 | 0.572 | 0.60 |
| KO D1/D2 | 4 | 80 173 920 | 76 111 634 | 1.053 | |
| KO D1/D2 P-ULK1 | 4 | 82 326 216 | 70 439 544 | 1.169 | 1.11 |
| KO Acox | 5 | 88 036 312 | 69 539 170 | 1.266 | |
| KO Acox P-ULK1 | 5 | 70 205 856 | 79 409 616 | 0.884 | 0.70 |

| | Ulk1 Transcriptomic |
|---|---|
| | Ratio Mean |
| WT | 1.0 |
| KO D1 | 0.9 |
| KO D2 | 1.0 |
| KO D1/D2 | 1.0 |
| KO Acox | 0.8 |

| WB Ulk1 (3 Mb) | | |
|---|---|---|
| | Moyenne Ratio | Ecart-type Ratio |
| WT | 1 | 0 |
| KO D1 | 1.77 | 0.38 |
| KO D2 | 1.36 | 0.16 |
| KO D1/D2 | 1.82 | 0.24 |
| KO Acox | 1.47 | 0.54 |

| WB p-Ulk1 (3 Mb) | | |
|---|---|---|
| | Moyenne Ratio | Ecart-type Ratio |
| WT | 1 | 0 |
| KO D1 | 1.22 | 0.59 |
| KO D2 | 1.05 | 0.31 |
| KO D1/D2 | 2.05 | 0.46 |
| KO Acox | 1.43 | 0.44 |

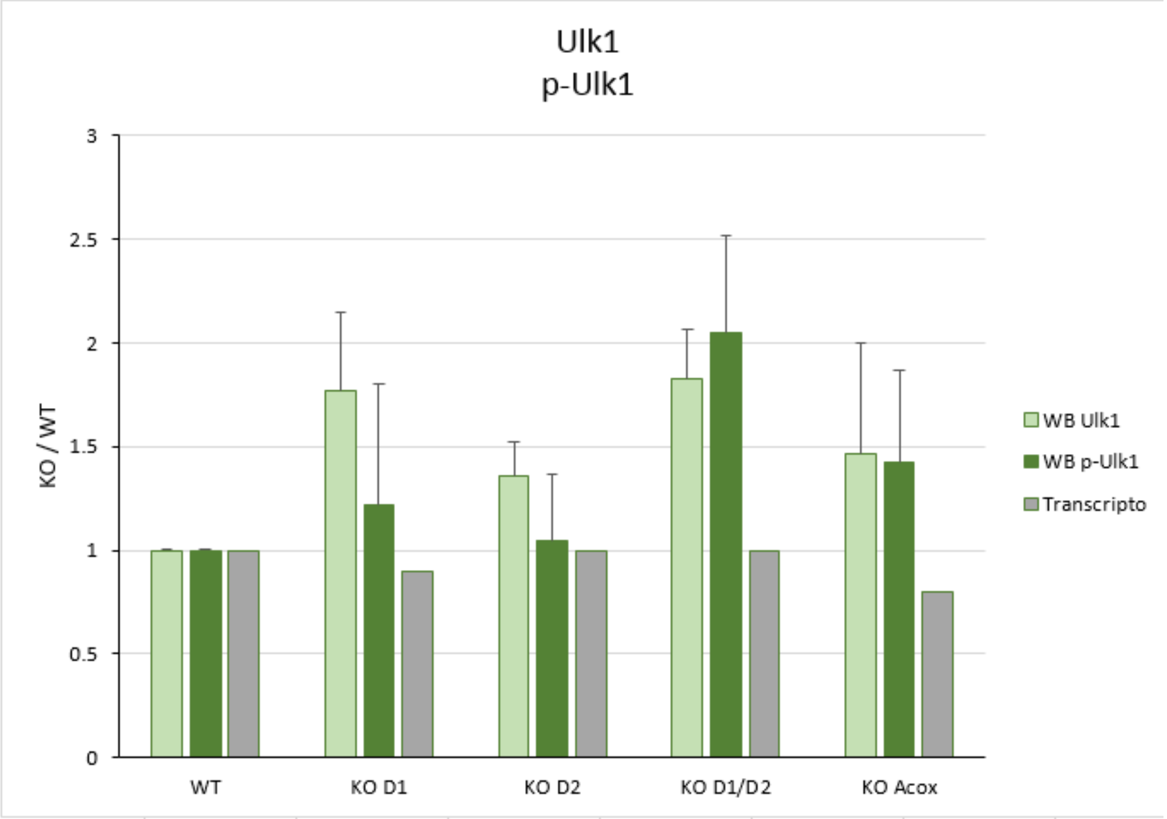


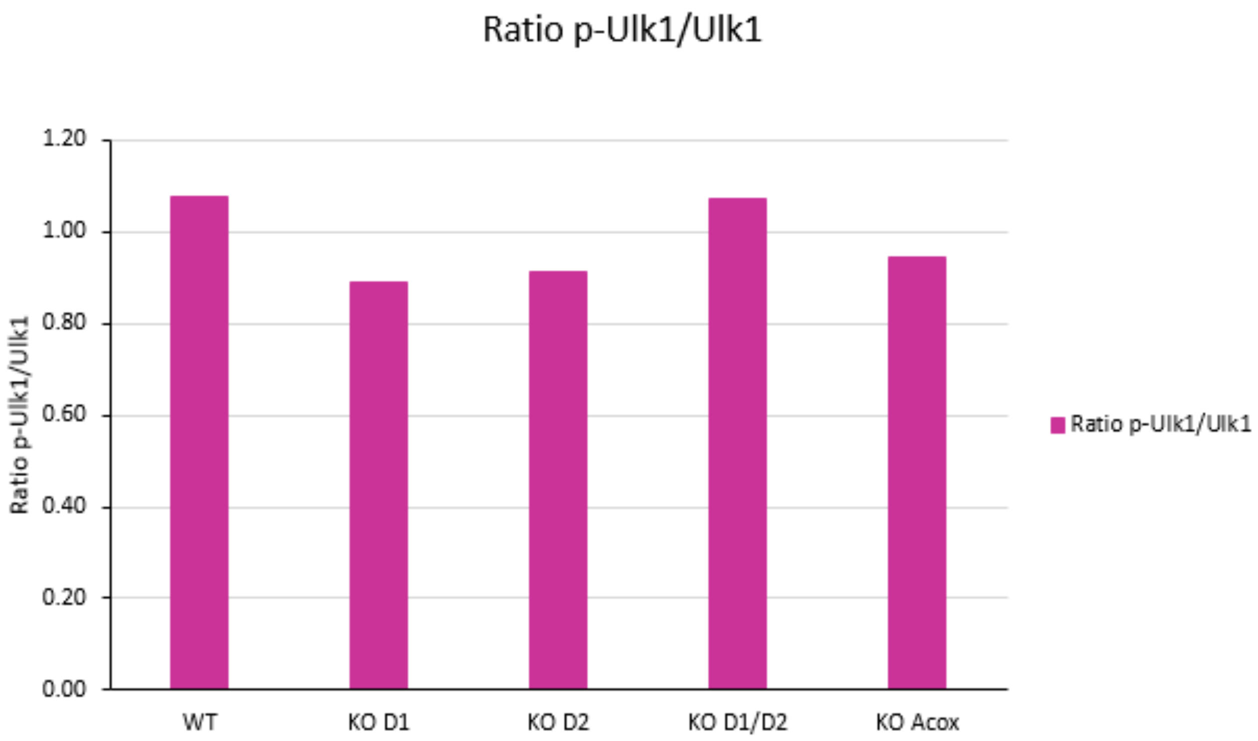

**ApoE**

# Samples serie NS 5
# WB Catherine 9/07/2021 – Mb 1

*Mb selected for the publication*

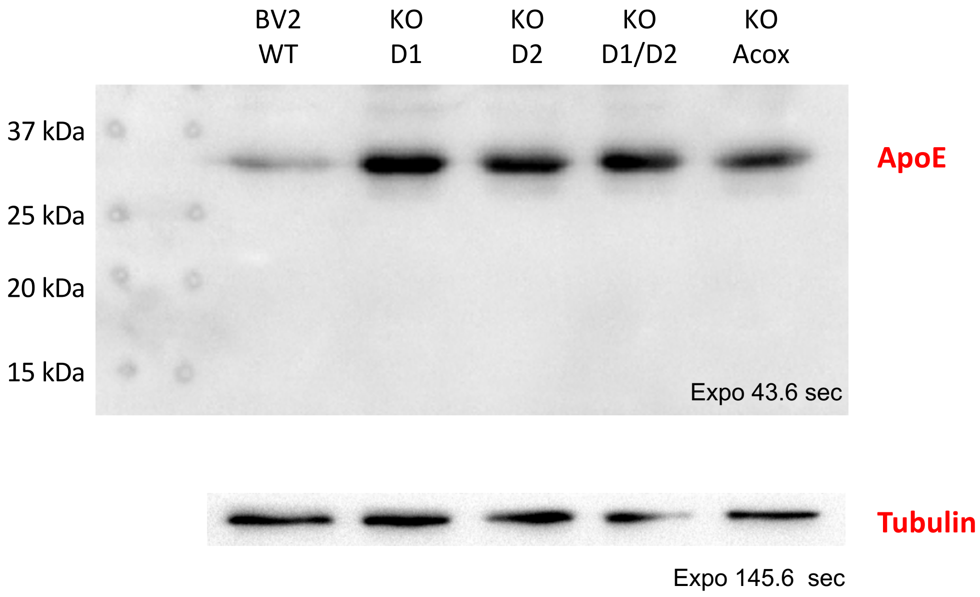


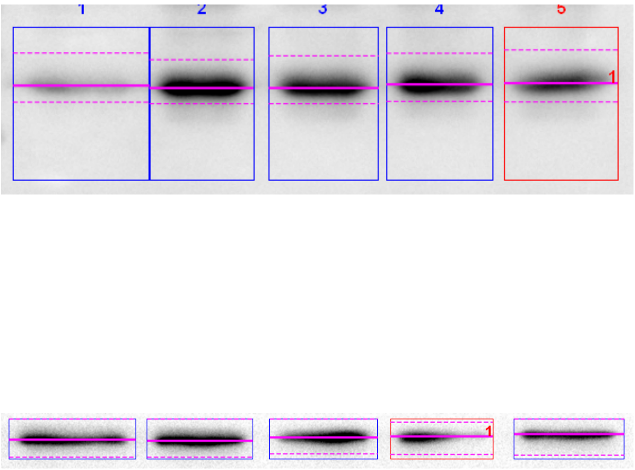

**ApoE**

MW : 35 kDa

Antibody : Abcam # ab183597

14% Gel
30 µg/load
PVDF Transfer

## Densitometric analysis

| Série NS 5 | Apo E | Tubuline | Ratio Apo E/tub | Ratio /WT |
|---|---|---|---|---|
| WT | 19 472 499 | 44 043 900 | 0.442 | 1.00 |
| KO D1 | 55 029 766 | 44 422 000 | 1.239 | 2.80 |
| KO D2 | 45 672 263 | 37 333 457 | 1.223 | 2.77 |
| KO D1/D2 | 43 554 280 | 21 764 992 | 2.001 | 4.53 |
| KO Acox | 36 256 646 | 27 966 510 | 1.296 | 2.93 |

# ApoE

## Samples serie NS 7
## WB Ali Mb 65

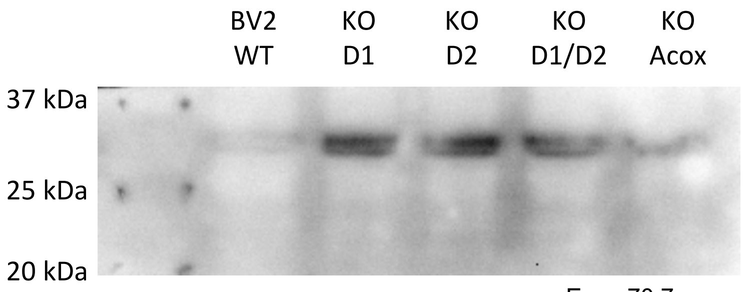


Expo 70.7 sec

ApoE

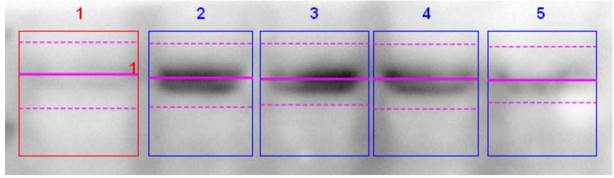


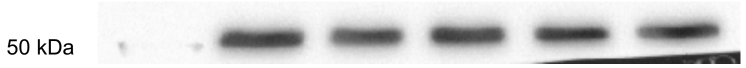


Expo 363.9 sec

Tubulin

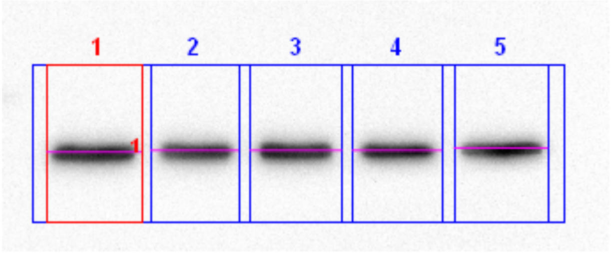


14% Gel
30 µg/load
PVDF Transfer

## Densitometric analysis

| série NS 7 | Apo E | Tubuline | Ratio Apo E/tub | Ratio KO/WT |
|---|---|---|---|---|
| **WT** | 16 476 774 | 97 497 884 | 0.169 | **1.00** |
| **KO D1** | 43 325 467 | 76 393 692 | 0.567 | **3.36** |
| **KO D2** | 45 684 669 | 86 879 033 | 0.526 | **3.11** |
| **KO D1/D2** | 39 935 520 | 79 983 860 | 0.499 | **2.95** |
| **KO Acox** | 20 205 180 | 81 275 560 | 0.249 | **1.47** |

**ApoE**

# Samples serie NS 8
# WB Ali Mb 66

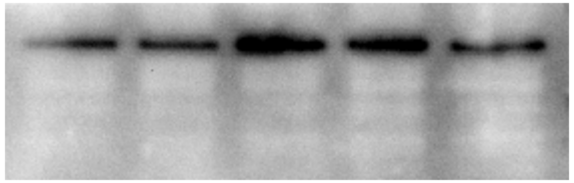


Expo 52.7 sec

**ApoE**

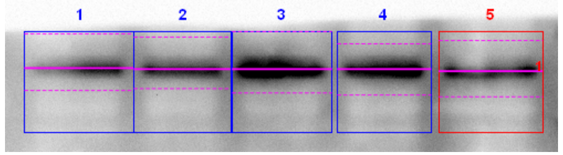


14% Gel
30 µg/load
PVDF Transfer

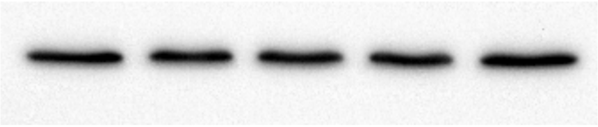

Expo 363.9 sec

**Tubulin**

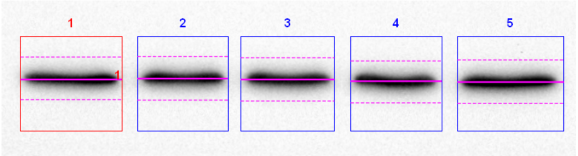


## Densitometric analysis

| série NS 8 - analyse densito bis | Apo E | Tubuline | Ratio Apo E/tub | Ratio KO/WT |
|---|---|---|---|---|
| **WT** | 31 132 780 | 83 521 816 | 0.373 | **1.00** |
| **KO D1** | 32 783 608 | 72 918 884 | 0.450 | **1.21** |
| **KO D2** | 58 154 278 | 75 441 960 | 0.771 | **2.07** |
| **KO D1/D2** | 46 151 529 | 74 028 592 | 0.623 | **1.67** |
| **KO Acox** | 39 728 552 | 95 235 250 | 0.417 | **1.12** |

# ApoE

| | Transcriptomique |
|---|---|
| | Moyenne Ratio |
| WT | 1,00 |
| KO D1 | 2,16 |
| KO D2 | 2,76 |
| KO D1/D2 | 1,68 |
| KO Acox | 1,39 |

| Mb 65, 66, Mb1 | | |
|---|---|---|
| | Moyenne Ratio | Ecart-type Ratio |
| WT | 1 | 0 |
| KO D1 | 2,45 | 1,12 |
| KO D2 | 2,65 | 0,53 |
| KO D1/D2 | 3,05 | 1,43 |
| KO Acox | 1,84 | 0,96 |

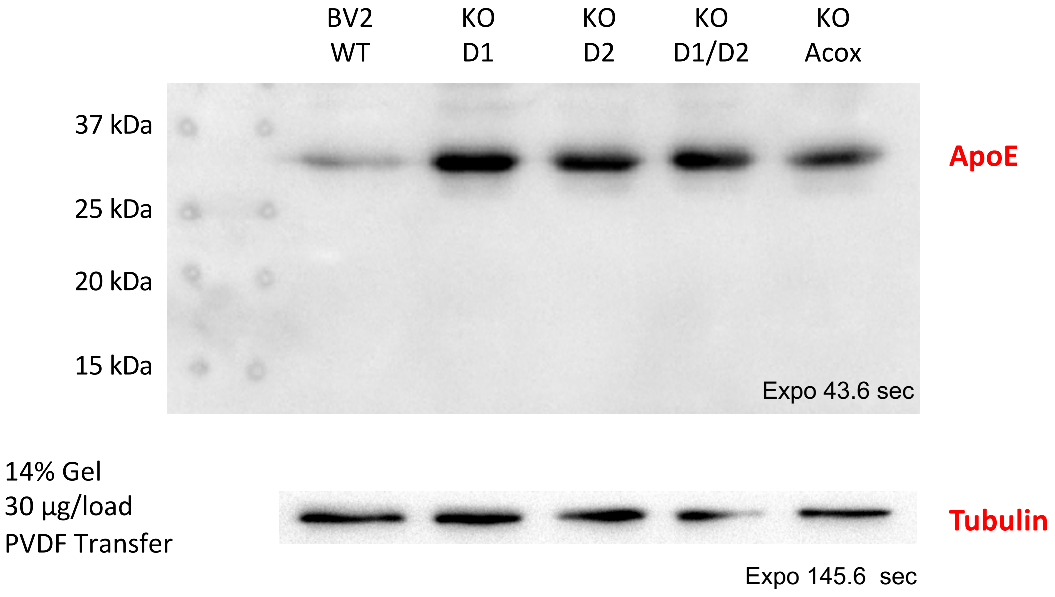


14% Gel
30 µg/load
PVDF Transfer

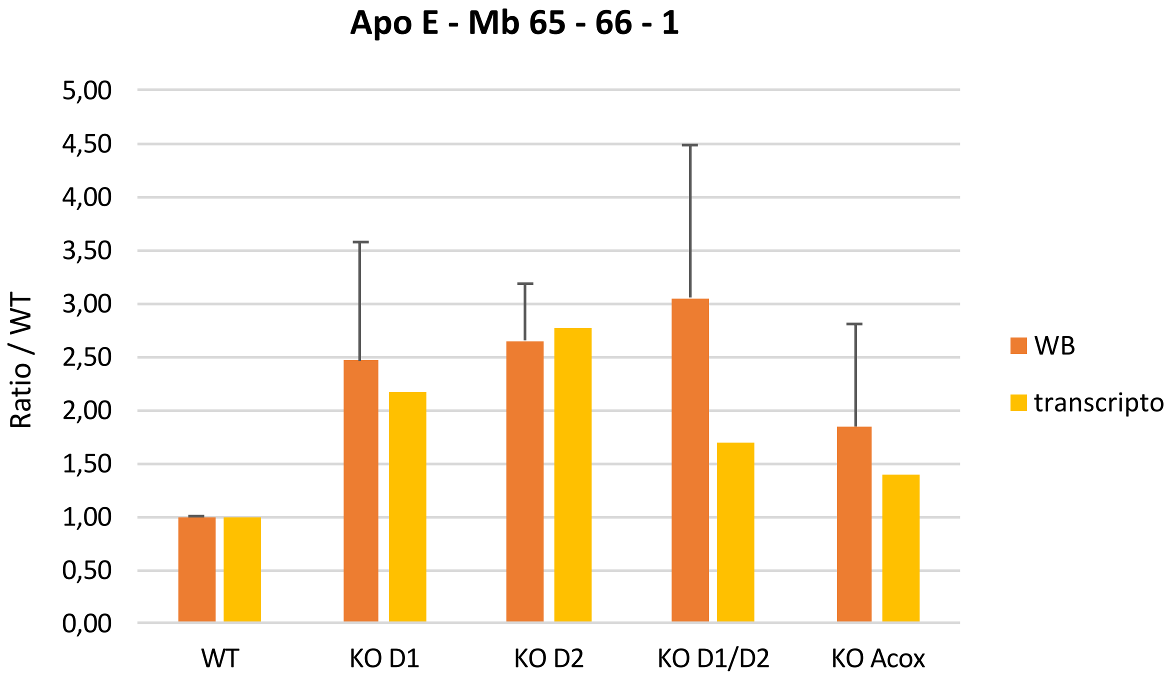

**CD36**

# Samples serie NS 2
# WB Ali – Mb 49

| BV2 WT | KO D1 | KO D2 | KO D1/D2 | KO Acox |
|---|---|---|---|---|

100 kDa

75 kDa

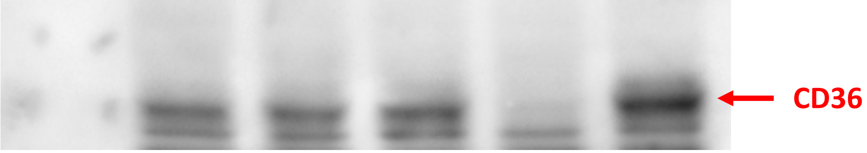

CD36

Expo 58.3 sec

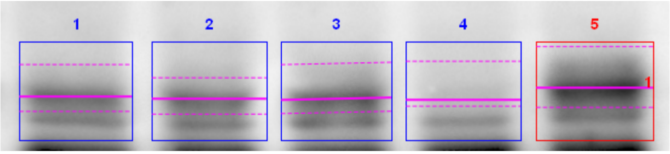


**CD36**

MW : 52 kDa
Observed : 75 kDa

Antibody : R&D Systems # AF2519

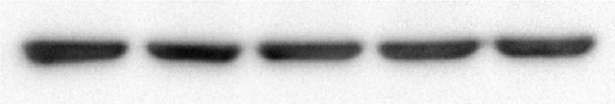

Tubulin

Expo 263.7 sec

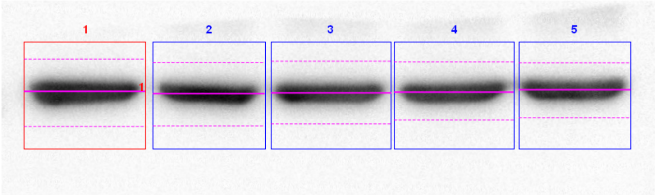


8% Gel
30 µg/load
PVDF Transfer

## Densitometric analysis

| Série NS 2 | CD36 | Tubuline | Ratio CD36/tub | Ratio /WT |
|---|---|---|---|---|
| WT | 35 735 088 | 164 335 548 | 0.217 | 1.00 |
| KO D1 | 26 149 760 | 140 699 952 | 0.186 | 0.85 |
| KO D2 | 41 838 200 | 139 700 880 | 0.299 | 1.38 |
| KO D1/D2 | 16 032 224 | 131 735 296 | 0.122 | 0.56 |
| KO Acox | 79 139 600 | 125 485 056 | 0.631 | 2.90 |

**CD36**

# Samples serie NS 4
# WB Ali – Mb 50

*Mb selected for the publication*

| BV2 WT | KO D1 | KO D2 | KO D1/D2 | KO Acox |
|---|---|---|---|---|

100 kDa
75 kDa

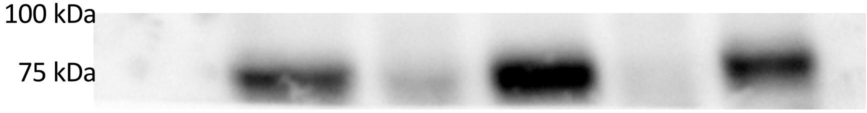

CD36

Expo 30.1 sec

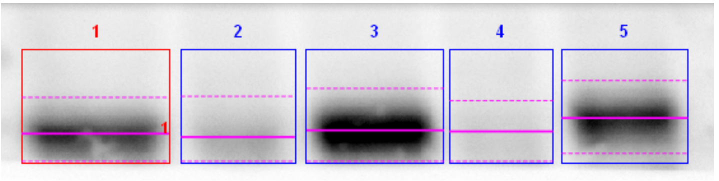


8% Gel
30 µg/load
PVDF Transfer

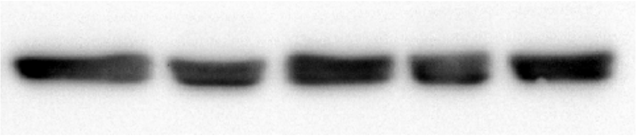

Tubulin

Expo 263.7 sec

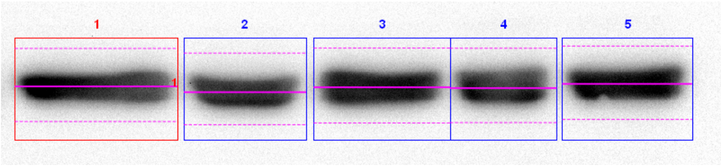


## Densitometric analysis

| série NS 4 | CD36 | Tubuline | Ratio CD36/tub | Ratio KO/WT |
|---|---|---|---|---|
| **WT** | 98 733 096 | 268 354 152 | 0.368 | **1.00** |
| **KO D1** | 19 145 706 | 179 114 688 | 0.107 | **0.29** |
| **KO D2** | 148 488 060 | 241 523 249 | 0.615 | **1.67** |
| **KO D1/D2** | 5 852 769 | 170 450 540 | 0.034 | **0.09** |
| **KO Acox** | 105 719 749 | 228 784 316 | 0.462 | **1.26** |

**CD36**

# Samples serie NS 5
# WB Ali – Mb 58

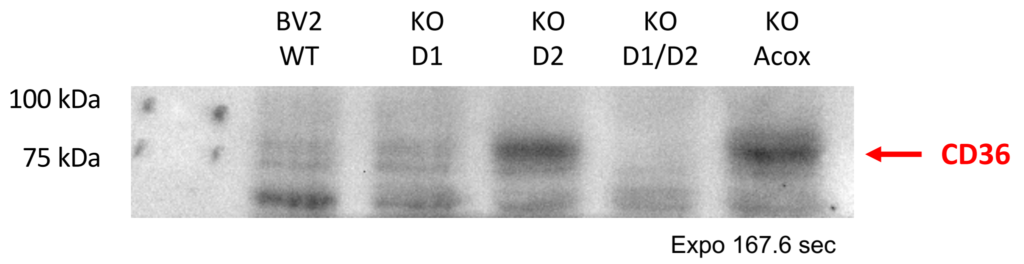


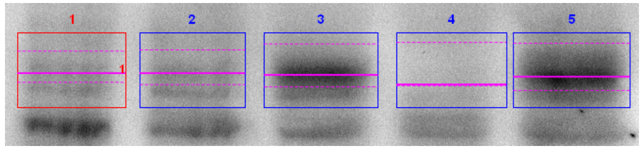


8% Gel
30 µg/load
PVDF Transfer

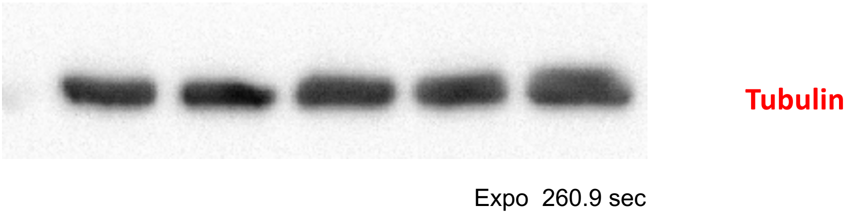


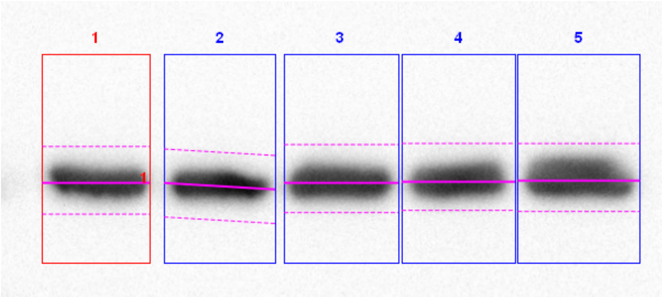


## Densitometric analysis

| série NS 5 | CD36 | Tubuline | Ratio CD36/tub | Ratio KO/WT |
|---|---|---|---|---|
| WT | 6 446 286 | 77 734 701 | 0.083 | 1.00 |
| KO D1 | 7 488 486 | 79 224 750 | 0.095 | 1.14 |
| KO D2 | 43 182 440 | 86 416 605 | 0.500 | 6.03 |
| KO D1/D2 | 3 320 704 | 80 797 464 | 0.041 | 0.50 |
| KO Acox | 48 836 104 | 95 471 820 | 0.512 | 6.17 |

# CD36

| | Transcriptomique |
|---|---|
| | Moyenne Ratio |
| WT | 1,00 |
| KO D1 | 0,66 |
| KO D2 | 2,88 |
| KO D1/D2 | 0,39 |
| KO Acox | 3,89 |

| Mb 49 - 50 - 58 | | |
|---|---|---|
| | Moyenne Ratio | Ecart-type Ratio |
| WT | 1 | 0 |
| KO D1 | 0,76 | 0,43 |
| KO D2 | 3,02 | 2,60 |
| KO D1/D2 | 0,38 | 0,25 |
| KO Acox | 3,44 | 2,50 |

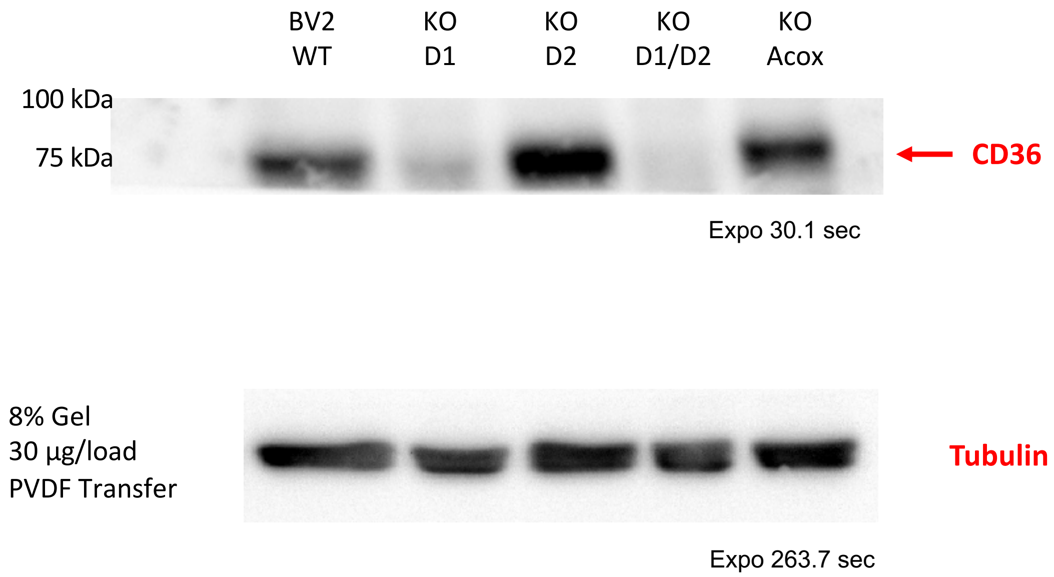


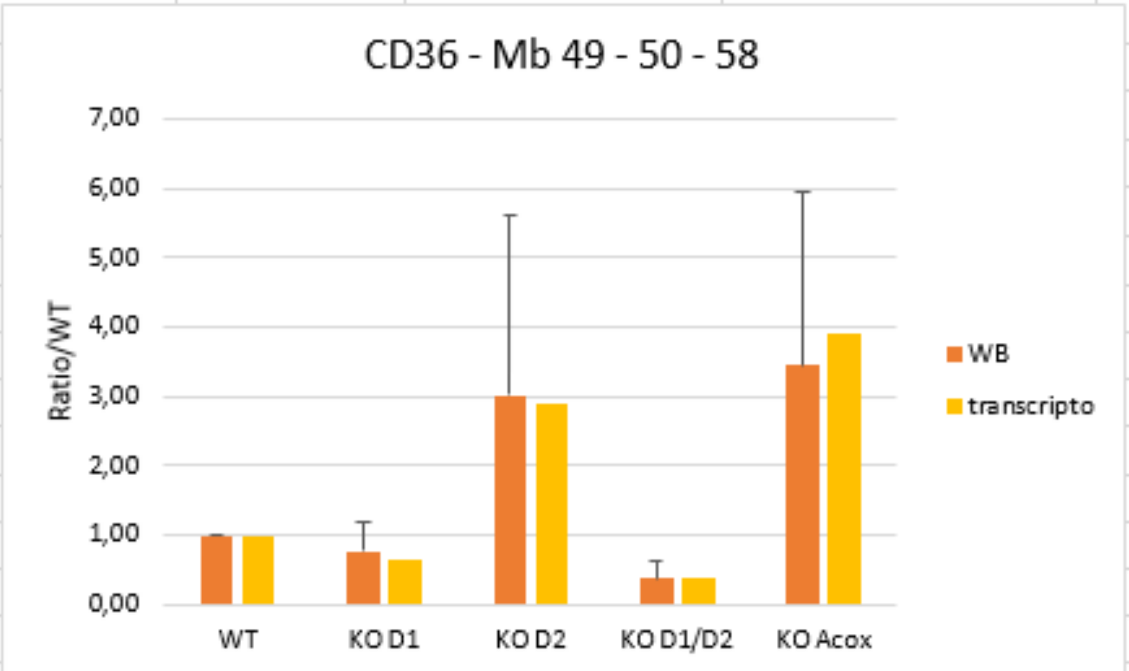

# Gpnmb

## 22/01/2021
## Samples serie 7

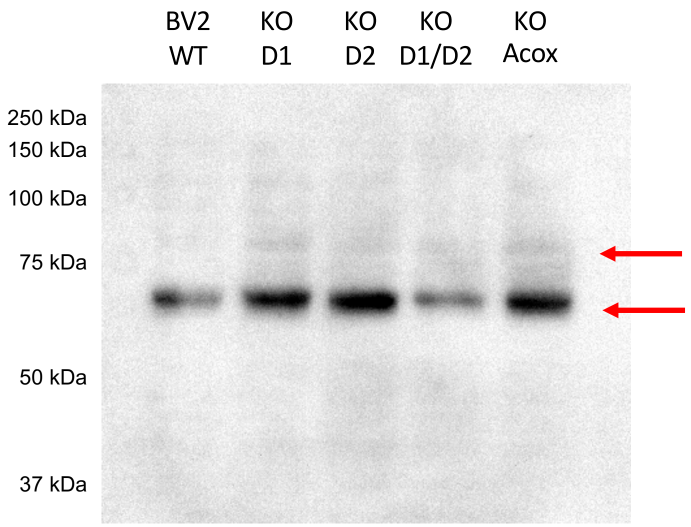


Gpnmb

Expo 347.7 sec

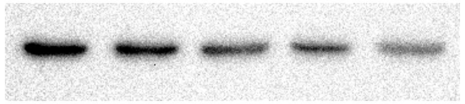

Tubulin

Expo 557.5 sec

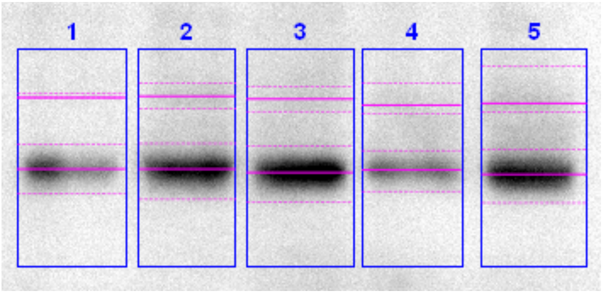


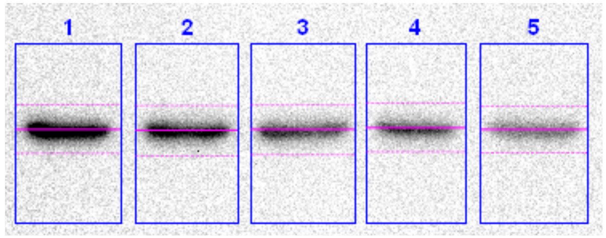


**GPNMB**

MW : 63 kDa

Antibody : Abcam # ab188222

8% Gel
30 µg/load
PVDF Transfer

## Densitometric analysis

| Série 7 - 22/01/2021 | GPNMB bande n°1 | GPNMB bande n°2 | Total GPNMB | Tubuline | Ratio GPNMB/tub | Ratio /WT |
|---|---|---|---|---|---|---|
| WT | 2 175 870 | 65 269 146 | 67 445 016 | 43 715 500 | 1.543 | 1.00 |
| KO D1 | 5 618 514 | 100 566 997 | 106 185 511 | 33 766 794 | 3.145 | 2.04 |
| KO D2 | 3 494 040 | 115 293 840 | 118 787 880 | 26 234 184 | 4.528 | 2.93 |
| KO D1/D2 | 8 282 960 | 53 467 120 | 61 750 080 | 21 120 348 | 2.924 | 1.90 |
| KO Acox | 16 451 631 | 106 300 082 | 122 751 713 | 17 712 563 | 6.930 | 4.49 |

# Gpnmb

## 11/02/2021
## Samples serie

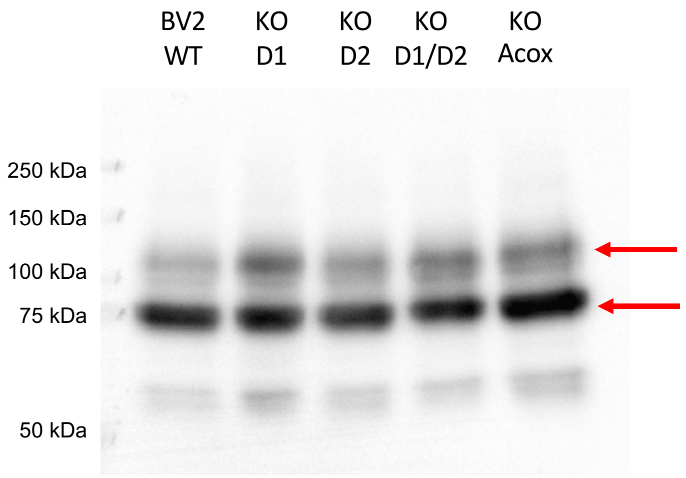


**Gpnmb**

Expo 16.9 sec

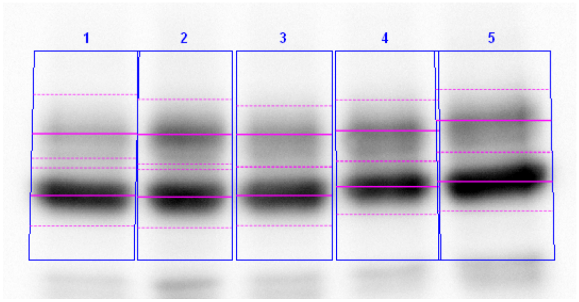


8% Gel
30 µg/load
PVDF Transfer

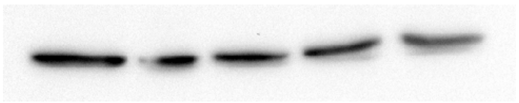

**Tubulin**

Expo 175.0 sec

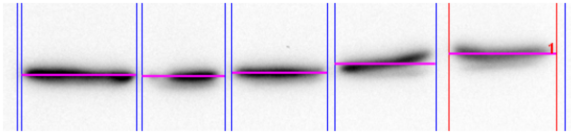

## Densitometric analysis

| Série - 11/02/2021 | GPNMB bande n°1 | GPNMB bande n°2 | Total GPNMB | Tubuline | Ratio GPNMB/tub | Ratio /WT |
|---|---|---|---|---|---|---|
| WT | 37 638 315 | 168 123 956 | 205 762 271 | 64 060 590 | 3.212 | 1.00 |
| KO D1 | 79 614 464 | 133 533 440 | 213 147 904 | 33 762 204 | 6.313 | 1.97 |
| KO D2 | 45 815 640 | 138 978 710 | 184 794 350 | 44 698 355 | 4.134 | 1.29 |
| KO D1/D2 | 58 854 951 | 133 166 886 | 192 021 837 | 45 095 040 | 4.258 | 1.33 |
| KO Acox | 73 379 916 | 213 424 701 | 286 804 617 | 39 646 080 | 7.234 | 2.25 |

**Gpnmb**

**25/02/2021**
**Samples serie**

*Mb selected for the publication*

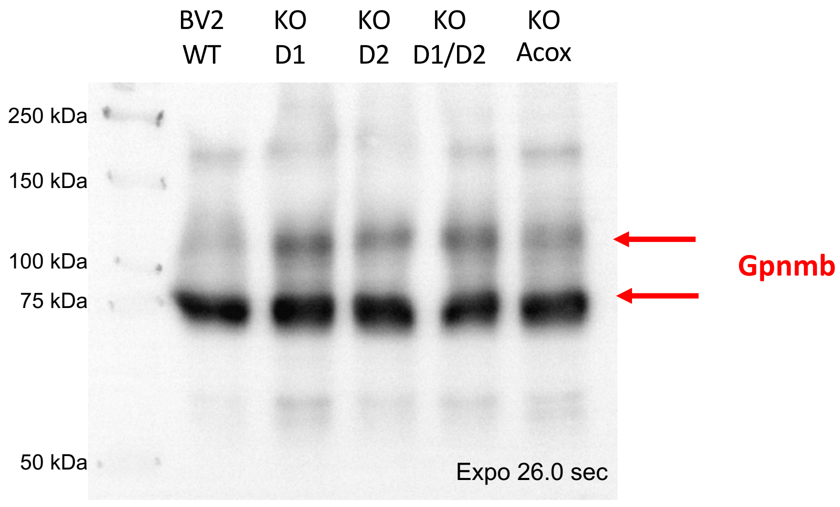


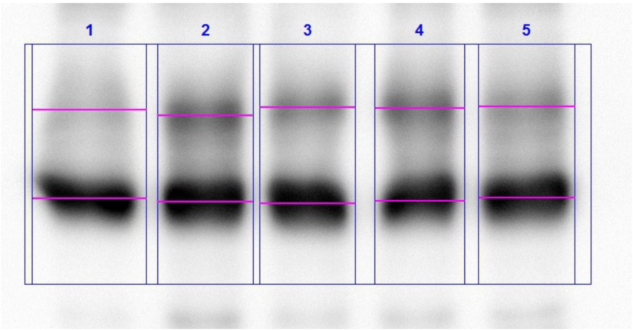


8% Gel
30 µg/load
PVDF Transfer

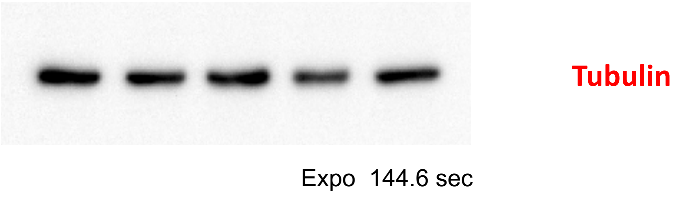


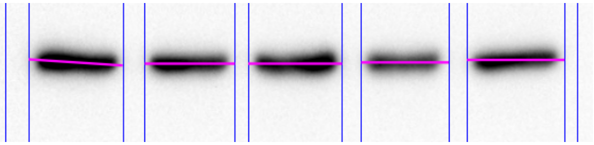

## Densitometric analysis

| Série - 25/02/2021 | GPNMB bande n°1 | GPNMB bande n°2 | Total GPNMB | Tubuline | Ratio GPNMB/tub | Ratio /WT |
|---|---|---|---|---|---|---|
| **WT** | 31 463 445 | 223 933 803 | 255 397 248 | 57 285 912 | 4.458 | 1.00 |
| **KO D1** | 75 774 054 | 193 900 196 | 269 674 250 | 43 064 525 | 6.262 | 1.40 |
| **KO D2** | 70 310 654 | 203 655 610 | 273 966 264 | 49 760 392 | 5.506 | 1.23 |
| **KO D1/D2** | 77 847 963 | 166 769 508 | 244 617 471 | 33 229 633 | 7.361 | 1.65 |
| **KO Acox** | 53 152 540 | 199 203 382 | 252 355 922 | 44 629 944 | 5.654 | 1.27 |

# Gpnmb

| | Transcriptomique Moyenne Ratio |
|---|---|
| **WT** | 1,0 |
| **KO D1** | 2,1 |
| **KO D2** | 2,4 |
| **KO D1/D2** | 2,0 |
| **KO Acox** | 2,4 |

| Mb 13, 22, 25 | | |
|---|---|---|
| | **Moyenne Ratio** | **Ecart-type Ratio** |
| **WT** | 1 | 0 |
| **KO D1** | 1.80 | 0.35 |
| **KO D2** | 1.82 | 0.97 |
| **KO D1/D2** | 1.62 | 0.29 |
| **KO Acox** | 2.67 | 1.65 |

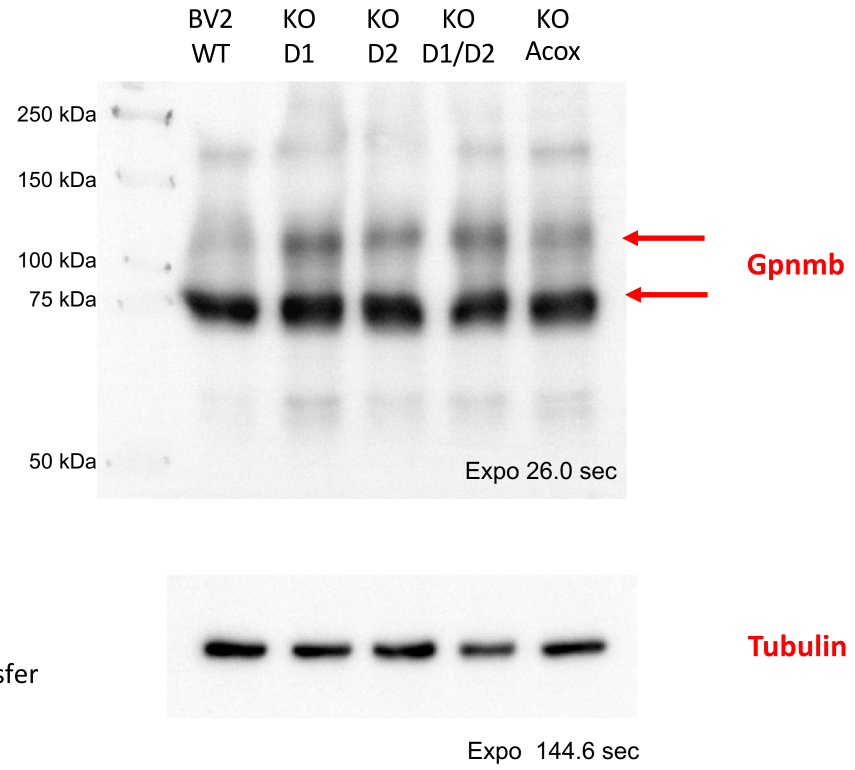


8% Gel
30 µg/load
PVDF Transfer

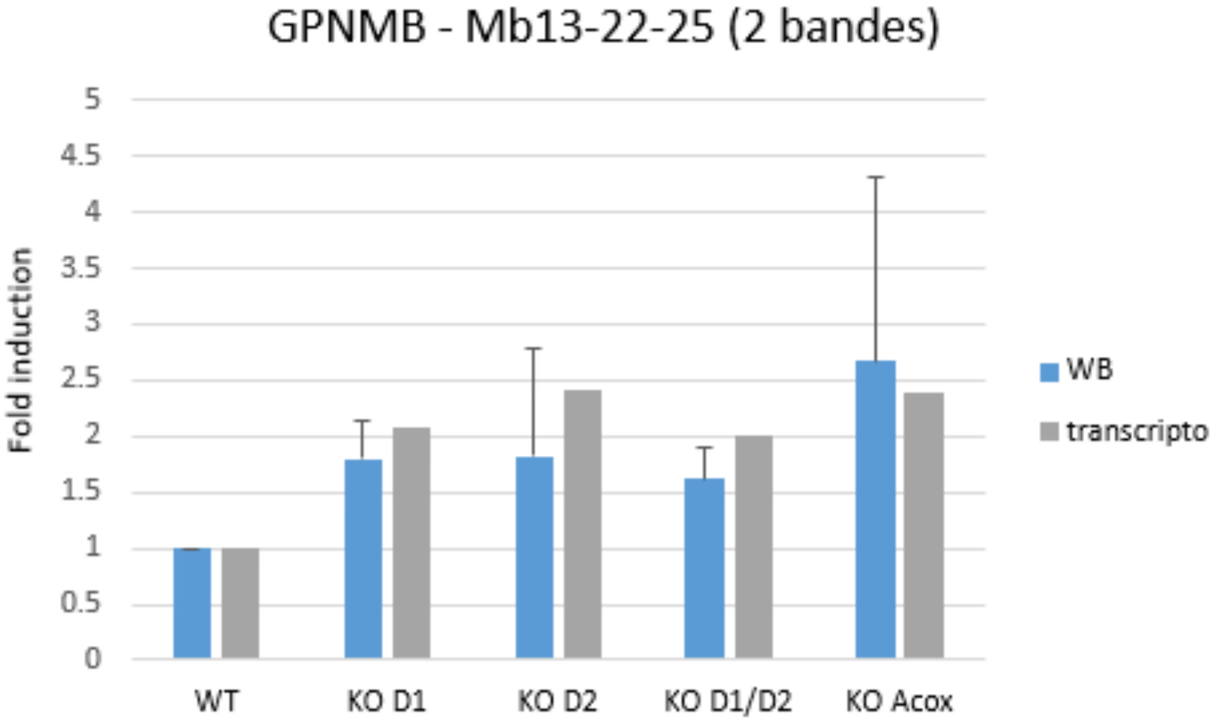

**Spp1**

# Samples serie S1 (21/10/21)
# WB Catherine 28/11/2022 – Mb « S1/2 »

*Mb selected for the publication*

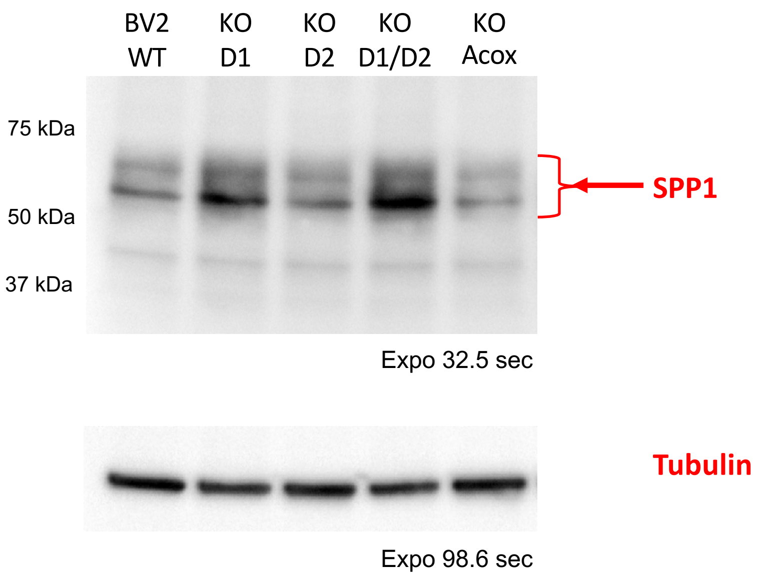


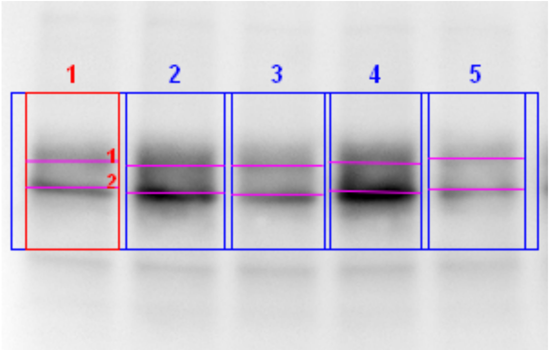


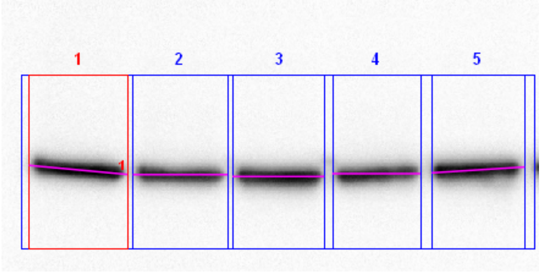


**SPP1**
MW : 32 kDa
Observed : 62-66 kDa

Antibody : R&D Systems # AF808

4-20% Gradient Gel
30 µg / load
PVDF transfer

## Densitometric analysis

| Série 1 - 21-10-2021 | SPP1 | | | Tubuline | Ratio SPP1/tub | Ratio /WT |
|---|---|---|---|---|---|---|
| | bande haute | bande basse | total | | | |
| WT | 39 140 309 | 42 336 090 | **81 476 399** | 73 256 085 | 1.112 | **1.00** |
| KO D1 | 63 128 658 | 79 092 035 | **142 220 693** | 59 328 612 | 2.397 | **2.16** |
| KO D2 | 40 476 252 | 43 635 090 | **84 111 342** | 70 202 104 | 1.198 | **1.08** |
| KO D1/D2 | 53 321 928 | 103 003 884 | **156 325 812** | 57 971 398 | 2.697 | **2.42** |
| KO Acox | 26 401 738 | 32 732 164 | **59 133 902** | 74 804 103 | 0.791 | **0.71** |

Spp1

# Samples serie S2 (27/10/21)
# WB Catherine 28/11/2022 – Mb «S1/2 »

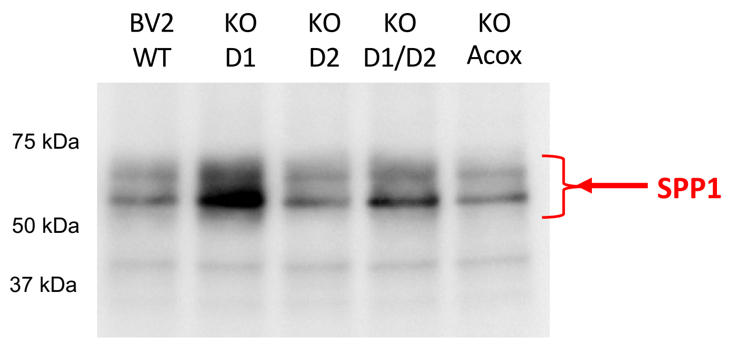


Expo 26.4 sec

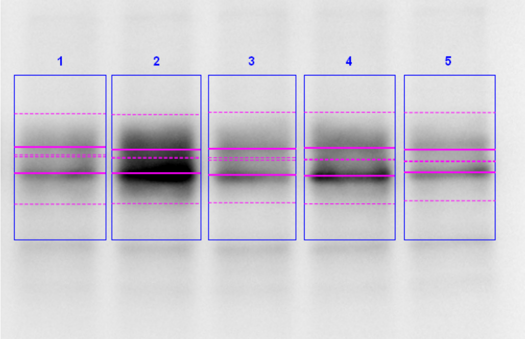


4-20% Gradient Gel
30 µg / load
PVDF transfer

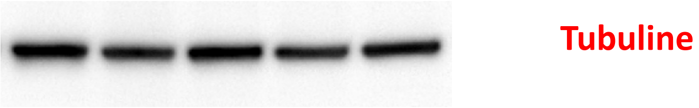


Expo 98.6 sec

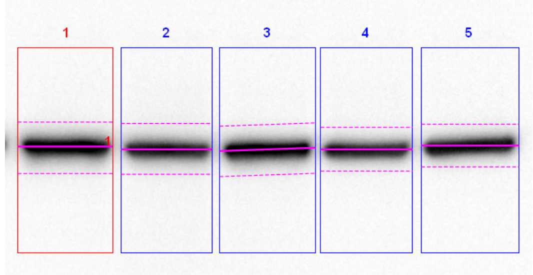


## Densitometric analysis

| Série 2 - 27-10-2021 | SPP1 | | | Tubuline | Ratio SPP1/tub | Ratio /WT |
|---|---|---|---|---|---|---|
| | bande haute | bande basse | total | | | |
| WT | 26 358 045 | 38 196 165 | 64 554 210 | 89 670 600 | 0.720 | 1.00 |
| KO D1 | 57 049 722 | 94 378 968 | 151 428 690 | 69 270 432 | 2.186 | 3.04 |
| KO D2 | 31 584 215 | 34 464 432 | 66 048 647 | 84 534 677 | 0.781 | 1.09 |
| KO D1/D2 | 41 504 632 | 55 726 736 | 97 231 368 | 63 023 519 | 1.543 | 2.14 |
| KO Acox | 26 682 915 | 26 310 060 | 52 992 975 | 72 146 041 | 0.735 | 1.02 |

**Spp1**

# Samples serie S2 (28/10/21)
# WB Catherine 22/11/2022 – Mb «S2/3 »

Gradient Gel 4-20% 15 puits
Turbo-blot Transfer
30 µg / load
PVDF Mb

| BV2 WT | KO D1 | KO D2 | KO D1/D2 | KO Acox |
|---|---|---|---|---|

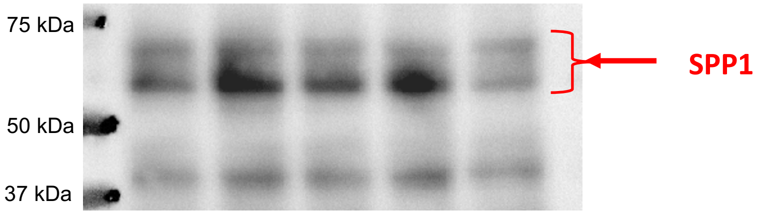


Expo 10.4 sec

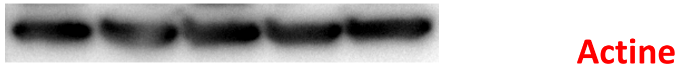


Expo 156.6 sec

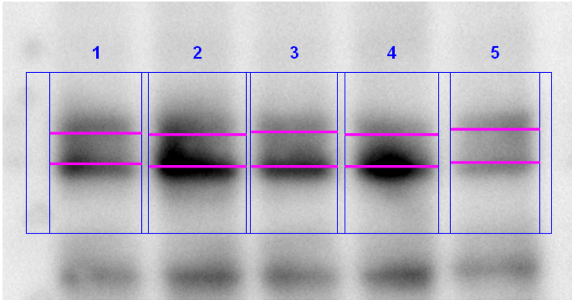


4-20% Gradient Gel
30 µg / load
PVDF transfer

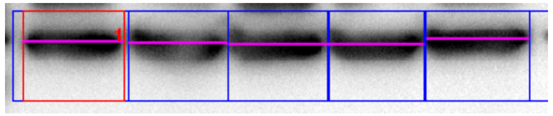

## Densitometric analysis

| Série 3 - 28-10-2021 | SPP1 | | | Actine | Ratio SPP1/tub | Ratio KO/WT |
|---|---|---|---|---|---|---|
| | bande haute | bande basse | total | | | |
| **WT** | 26 184 120 | 33 022 572 | **59 206 692** | 68 850 984 | 0.860 | **1.00** |
| **KO D1** | 30 543 127 | 56 772 538 | **87 315 665** | 66 069 675 | 1.322 | **1.54** |
| **KO D2** | 18 265 195 | 37 812 255 | **56 077 450** | 72 841 500 | 0.770 | **0.90** |
| **KO D1/D2** | 14 943 810 | 49 483 840 | **64 427 650** | 65 265 336 | 0.987 | **1.15** |
| **KO Acox** | 17 830 911 | 16 207 903 | **34 038 814** | 70 797 034 | 0.481 | **0.56** |

# Spp1

| | Transcriptomic |
|---|---|
| | Ratio Mean |
| WT | 1.0 |
| KO D1 | 3.6 |
| KO D2 | 2.0 |
| KO D1/D2 | 3.8 |
| KO Acox | 1.5 |

| Mb S1/2 S1 - S1/2 S2 - S2/3 S3 | | |
|---|---|---|
| | Ratio Mean | Ratio SD |
| WT | 1 | 0 |
| KO D1 | 2.24 | 0.75 |
| KO D2 | 1.02 | 0.11 |
| KO D1/D2 | 1.91 | 0.67 |
| KO Acox | 0.76 | 0.24 |

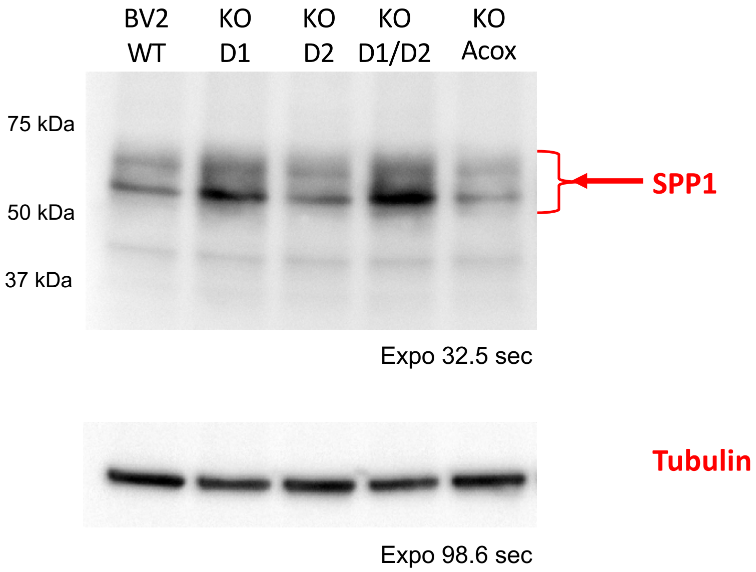


4-20% Gradient Gel
30 µg / load
PVDF transfer

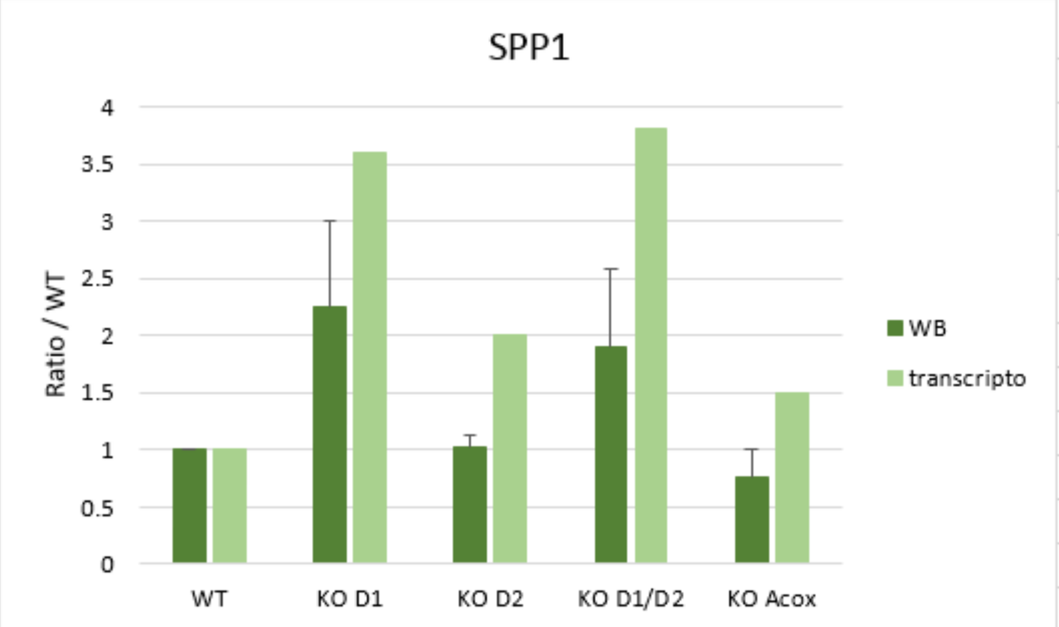

Supplement: Supplementary file 4 [file Data_Sheet_1.PDF]
